# Synthesis of Novel Benzofuran Spiro-2-Pyrrolidine Derivatives via [3+2] Azomethine Ylide Cycloadditions and Their Antitumor Activity

**DOI:** 10.3390/ijms252413580

**Published:** 2024-12-19

**Authors:** Bowen Pan, Tao Wang, Liangliang Zheng, Zhangchao Dong, Lijuan Liu, Xiongwei Liu, Tingting Feng, Ying Zhou, Yang Shi

**Affiliations:** 1 College of Pharmacy, Guizhou University of Traditional Chinese Medicine, Guiyang 550025, China; bwpan1105@163.com (B.P.); 18285244764@163.com (T.W.); zx15117508142@163.com (L.Z.); dongzhangchao0824@163.com (Z.D.); llj18385314855@163.com (L.L.); liuxiongwei058@gzy.edu.cn (X.L.); ftt0809@163.com (T.F.); 2 State Key Laboratory of Natural and Biomimetic Drugs, School of Pharmaceutical Sciences, Peking University, Beijing 100191, China

**Keywords:** spiro-pyrrolidine compound, benzofuran, antitumor activities, molecular docking

## Abstract

A synthetic strategy of a three-component spiro-pyrrolidine compound based on benzofuran via an [3+2] azomethine ylide cycloaddition reaction is reported herein. Under mild optimal conditions, this reaction can quickly produce potentially bioactive compounds with a wide range of substrates, high yield, and simple operation. The desired products were obtained with a yield of 74–99% and a diastereomeric ratio (dr) of >20:1. Subsequently, the inhibitory effects of the compounds on the cell viability of the human cancer cell line HeLa and mouse cancer cell line CT26 were evaluated. Compounds **4b** (IC_50_ = 15.14 ± 1.33 µM) and **4c** (IC_50_ = 10.26 ± 0.87 µM) showed higher antiproliferative activities against HeLa cells than cisplatin (IC_50_ = 15.91 ± 1.09 µM); compounds **4e** (IC_50_ = 8.31 ± 0.64 µM) and **4s** (IC_50_ = 5.28 ± 0.72 µM) exhibited better inhibitory activities against CT26 cells than cisplatin (IC_50_ = 10.27 ± 0.71 µM). The introduction of electron-donating substituents was beneficial to the inhibitory activities against cancer cells. Molecular docking simulations revealed that **4e** and **4s** may exert corresponding bioactivities by binding to antitumor targets through hydrogen bonds, providing a new approach for discovering spiro-heterocyclic antitumor drugs.

## 1. Introduction

Cancer is the second leading cause of death after heart disease [1]. With population growth and ageing, it is estimated that by 2030, the global cancer burden will increase to 21.7 million new cases and 13 million deaths [2]. Currently, the treatment of tumors mainly combines immunotherapy with chemotherapy [3]. However, the significant side effects of traditional antitumor chemical drugs limit their clinical application. During the treatment process, while killing cancer cells, they also kill normal tissue cells that are undergoing cell division, resulting in adverse reactions such as hair loss, nausea, and an impaired immune system, which have a particular impact on the physical and mental health of patients [4]. Traditional methods to reduce these side effects include reducing the drug dose, shortening the treatment duration, changing the administration method, and selecting drugs with lower toxicity. However, any of these methods may compromise the antitumor efficacy of the drug [4]. Therefore, it is urgent to find new antitumor drugs. In recent years, new anti-cancer drugs represented by molecular targeted therapies have emerged one after another, providing new options for cancer prevention and treatment. The apoptosis inhibitory gene [5] (Bcl-x1), vascular endothelial growth factor receptor 2 [6] (VEGFR2), tumor necrosis factor α [7] (TNFα), interleukin-2 [8] (IL-2), cyclin-dependent protein kinase 2 [9] (CDK-2), cyclooxygenase 2 [10] (Cox-2), farnesyl transferase [11] (FTase), and focal adhesion kinase [12] (FAK) are all proteins related to tumor occurrence and progression. They are essential in tumor metastasis, formation, cell migration, invasion, and angiogenesis. Inhibiting their expression can exert antitumor effects through many pathways and are some promising targets for targeted anti-cancer drugs. Although many active compounds and approved drugs target these proteins, problems such as inconsistent patient response rates, development of drug resistance, and side effects still exist. Based on their critical positions in oncology and their currently unmet treatment needs, the discovery of new compounds with novel structures and significantly enhanced activities is of great significance for broadening the treatment window, improving efficacy, and overcoming the limitations of existing drugs.

**Figure 1:**
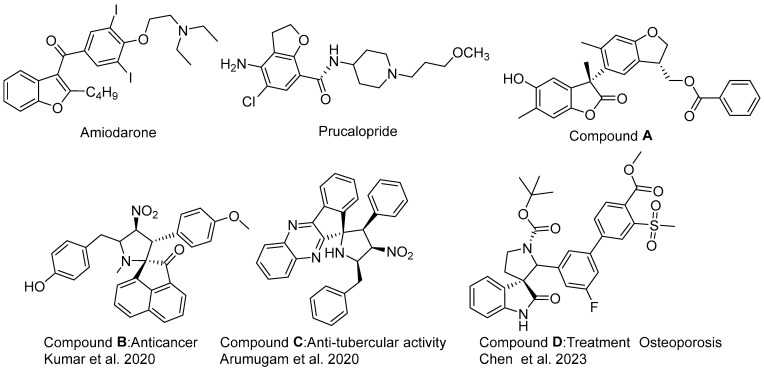
Benzofuran ring and spiro-pyrrolidine compounds [20,21,23].

The benzofuran skeleton is an essential heterocyclic compound widely distributed in nature, bioactive compounds, and clinical drugs [13]. For example, amiodarone is used for the treatment of ventricular arrhythmia, prucalopride is used for the treatment of chronic constipation [14], and compound **A** isolated from *Paeonia suffruticosa* has anti-proliferative activity against HL-60 leukemia cells [15] (IC_50_ = 6.8 μM) (Figure 1). In addition, nitrogen-containing heterocyclic compounds have perfect biological activities and have a particular impact on many physiological activities of the human body; for these reasons, medicinal chemists are highly concerned with them [16,17,18]. Spiro-pyrrolidine skeleton compounds in spiro heterocyclic systems have essential application value in the biomedical field and have antitumor [19,20], anti-tuberculosis [21], antibacterial [22], anti-osteoporotic [23], and anti-Alzheimer’s disease effects [24] (Figure 1). Therefore, efficient introduction of the spiro-pyrrolidine skeleton into the molecule is a way to discover novel pharmacologically active derivatives with more optimal properties.

Constructing target compounds with potential application value using simple and readily available raw materials without complex organic ligands or transition metal catalysis is one of the important development directions of contemporary organic synthesis research [25]. The [3+2] cycloaddition reaction refers to the cyclization addition between the three-atom component and derivatives such as alkynes. Among them, the cycloaddition reaction of azide and alkyne is widely used [26]. In addition, the [3+2] cycloaddition reaction is also a standard method for synthesizing five-membered heterocycles. The [3+2] cycloaddition reaction between dipoles and compounds possessing exo-cyclic double bonds, which serves as a crucial means for the synthesis of spiroheterocyclic compounds [27], is of particular interest due to its unique reactivity characteristics. Notably, this reaction has its processes involving azomethine ylides significantly accelerated when the alkene component is electrophilically activated due to the existence of EWG groups [28]. In the [3+2] cycloaddition reaction, the mechanism has a profound impact on stereochemistry. When the reaction is carried out in a stepwise mechanism, an unstable non-ring intermediate is formed, and rotation around the bond from the 2-π component to the target five-membered ring may be released, which may change the substituent configuration during the reaction, resulting in stereoselectivity restriction and the creation of additional stereoisomers [29]. This phenomenon cannot be ignored in synthetic practice, because it may affect the purity and yield of target products when constructing complex organic molecules.

We synthesized benzofuran spiro-pyrrolidine derivatives in this study through an efficient three-component [3+2] azomethine ylide cycloaddition reaction. The antiproliferative activities of the compounds against two cancer cells were evaluated using the MTT method, with the hope of discovering a series of new antitumor small molecule drugs.

## 2. Results and Discussion

### 2.1. Synthesis of Spiro-Pyrrolidine Compounds

The reaction conditions were optimized by varying the solvent, temperature, and substrate concentration, with the reaction between (Z)-3-benzylidenebenzofuran-2(3H)-one (**1a**), ninhydrin (**2**), and sarcosine (**3**) selected as the model reaction. Initially, the reaction was performed at 40 °C in acetonitrile, yielding the desired cycloadduct **4a** with a remarkable yield of 82% and a dr value of 9:1 after 16 h (Entry 1, Table 1). Encouraged by this result, various solvents were tested, including DCE, EtOAc, EtOH, toluene, H_2_O, and THF (Entry 2–7). Among these, THF was found to be the most effective, producing **4a** with a yield of 90% and a dr value of 15:1 in 21 h (Entry 7). Other solvents such as EtOAc, toluene, and EtOH also gave similar yields but lower dr values, while H_2_O was incompatible with the reaction conditions; even with the addition of TBAB, only trace amounts of the product were obtained (Entry 6).

Subsequent optimization focused on reaction temperature and substrate concentration in THF. Increasing the temperature improved the reaction efficiency, with a maximum yield of 99% achieved at 60 °C after 5 h (Entry 8). Further increases in temperature did not improve the yield (Entry 8–10). The substrate concentration was optimized to 0.1 M, ensuring the highest product yield under the identified optimal conditions: THF as the solvent, a reaction temperature of 60 °C, and a substrate concentration of 0.1 M, which together resulted in a yield of 99%.

**Scheme 1:**
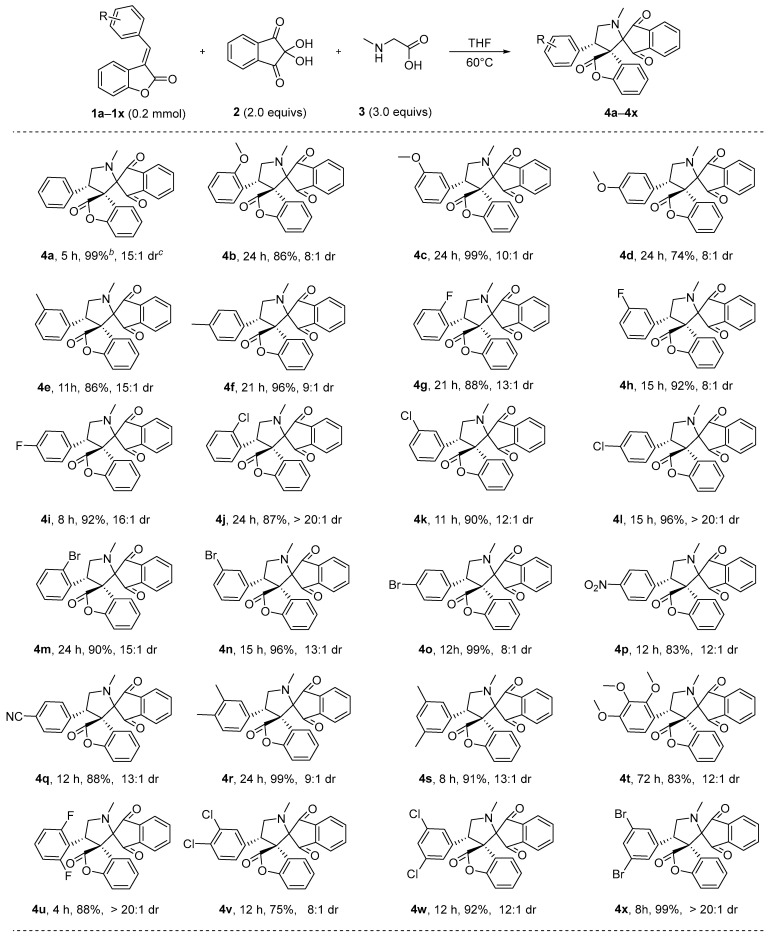
Substrate scope ^a^. ^a^ The reaction was conducted using **1** (0.3 mmol), **2** (0.6 mmol), and **3** (0.9 mmol) in THF (3.0 mL) at 60 °C.; ^b^ Yield of isolated product is given. ^c^ Determined based on the yield ratio of isomers separated by column chromatography.

Under optimized reaction conditions, the applicability of different 3-benzylbenzofurans **1a**–**x** to this (3+2) cycloaddition reaction was investigated to construct structurally complex spiro-pyrrolidine hybrids **4a**–**x**. As shown in Scheme 1, the introduction of electron-donating groups (**4b**–**4f** and **4r**–**4t**) and electron-withdrawing groups (**4g**–**4q** and **4u**–**4x**) on the benzene ring of 3-benzylbenzofuran had no significant effect on the reaction efficiency, and the desired products were obtained in 74–99% yields and with >20:1 dr. ^1^H NMR, ^13^C NMR, and HRMS confirmed the structures of all compounds.

### 2.2. In Vitro Effect on Viability of Cancer Cells

**Figure 2:**
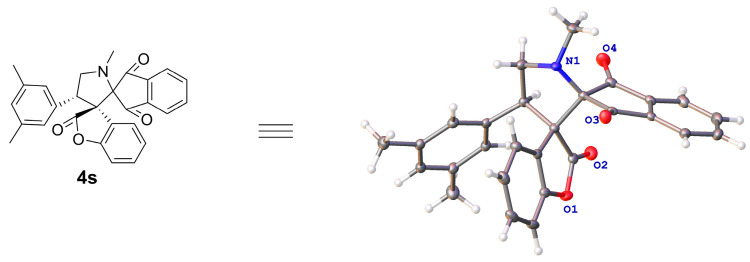
Structure of compound **4s** determined by X-ray crystallography.

We used cisplatin as a positive control to explore the antitumor activity of compounds **4a**–**4x**. We evaluated the effect of the compounds on the activity of the human cervical cancer cell line Hela and mouse colon cancer cell line CT26 by the MTT method. The IC_50_ values of the compounds are summarized in Table 2. In human cervical cancer, HeLa cells, compounds **4b** (IC_50_ = 15.14 ± 1.33 µM), **4c** (IC_50_ = 10.26 ± 0.87 µM), and **4s** (IC_50_ = 17.48 ± 1.36 µM) showed excellent antiproliferative activity, and the activities of **4b** and **4c** were higher than that of cisplatin (IC_50_ = 15.91 ± 1.09 µM). For mouse colon cancer CT26 cells, compounds **4e** (IC_50_ = 8.31 ± 0.64 µM), **4s** (IC_50_ = 5.28 ± 0.72 µM), and **4v** (IC_50_ = 14.34 ± 0.65 µM) showed significant cancer cell inhibitory activity. Among them, the antiproliferative activities of compounds **4e** and **4s** were better than those of cisplatin (IC_50_ = 10.27 ± 0.71 µM). The electronegativity, number, and position of substituents play an essential role in the activity of compounds. A preliminary analysis of the structure–activity relationship found that introducing electron-donating substituents benefits the inhibitory activity of cancer cells. However, the introduction of the electron-withdrawing substituents **-Cl** and **-Br** did not significantly improve the activity of the compounds. Only compounds **4g**–**4i**, **4u**, and compound **4q** with strong electronegativity **-F** substituents showed better activity than the parent compound **4a** in HeLa cells. The three-dimensional structure of compound **4s** with the best activity was further confirmed by X-ray crystallography (Figure 2 and Tables S1–S7 in the Supporting Information). The crystallographic data of compound **4s** have been deposited in the Cambridge Crystallographic Data Centre (CCDC 2403491).

### 2.3. Molecular Docking 

The method of computer-aided molecular docking was used to study the binding situation of compounds **4b**, **4c**, **4e**, and **4s** with different antitumor targets to explore the possible mechanism of action [30]. As shown in Table 3, the larger the absolute value of the score, the stronger the binding affinity. The docking scores of compounds **4e** and **4s** with eight target proteins were better than those of compounds **4b** and **4c**. In addition, the docking effects of the proteins TNFα and FTase with the four compounds were better than the other six proteins. The docking score ranges were −8.793 to −4.919, and −8.226 to −4.457, respectively, and they had a high affinity with the corresponding target proteins. In order to clarify the interaction between compounds **4e** and **4s** and the proteins TNFα and FTase, using Discovery Studio 2019 Client software, the possible binding mode of the ligand–receptor complex at the binding site was analyzed.

**Figure 3:**
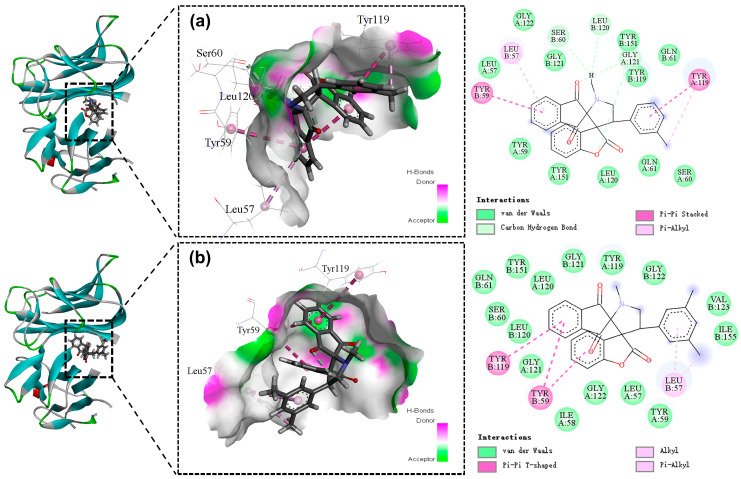
Mode of action of the protein TNFα with **4b** (**a**) and **4c** (**b**).

**Figure 4:**
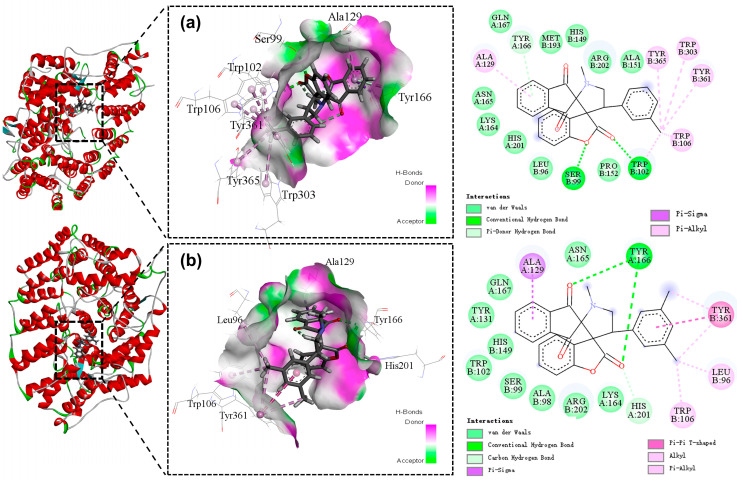
Mode of action of the protein FTase with **4e** (**a**) and **4s** (**b**).

The 3D diagram shows that compounds **4e** and **4s** are bound in the middle active pocket of TNFα, and both generate π-π conjugation with amino acid residues of the protein to maintain binding. The 2D diagram shows compound **4e** forms two π-π conjugation interactions with TNFα protein residues Tyr 59(B) and Tyr 119(A). Compound **4s** forms three π-π conjugations with Tyr 119(B) and Tyr 59(B) (Figure 3). In the interaction mode of protein FTase with **4e** and **4s**, the 3D diagram shows that the active pocket of FTase almost wraps small molecules **4e** and **4s** and has hydrogen bond binding. In the 2D diagram, the oxygen atom and carbonyl group on the benzofuran ring of compound **4e** form hydrogen bonds with protein residues Ser 99(B) and Trp 102(B), respectively. Compound **4s** forms two hydrogen bonds with Tyr 166(A), forms π-π conjugation with Tyr 361(B), and forms π-σ interaction with Ala 129(A) (Figure 4). In addition, some weak interactions, such as π-alkyl bonds and carbon–hydrogen bonds, may have little effect on activity. However, they should not be ignored because the interaction between ligands and drug targets may be underestimated. For example, compound **4e** forms four carbon–hydrogen bonds with the protein TNFα and five π-Alkyl bonds with the protein FTase. These results provide an essential basis for further understanding the protein–compound interaction mechanism.

## 3. Materials and Methods

### 3.1. General Information

Progress of the chemical reactions was monitored by thin-layer chromatography (TLC) under UV light. Compound purification was carried out using flash chromatography on silica gel. Chemical yields refer to pure isolated substances. HRMS spectra of the compounds were measured on a Waters Xevo G2-S QTOF mass spectrometer using the electron spray ionization (ESI) method. ^1^H, ^13^C and ^19^F NMR spectra were obtained using a Bruker DPX-400 MHz spectrometer. Chemical shifts were reported in ppm from CDCl_3_ or TMS with the solvent resonance as an internal standard. The following abbreviations were used to designate chemical shift multiplicities: s = singlet, d = doublet, t = triplet, q = quartet, h = heptet, m = multiplet, and br = broad.

### 3.2. Synthesis of the Compounds 4a-4x and 1a-1x

Referring to the scheme reported by Ramesh D et al. [31], we added benzofuranone (1 equiv), corresponding substituted benzaldehyde (1.2 equiv), anhydrous ethanol, and piperidine (0.2 equiv) to the round-bottom flask successively. Then, the reaction process (V_PE_:V_EA_ = 3:1) was monitored by TLC at 80 °C. After the reaction was completed, the solvent was recovered by rotary evaporation and purified using a silica gel column (V_PE_:V_EA_ = 30:1) or recrystallization (DCM and EtOH) to obtain compound **1a**–**1x**, which were both yellow solids.

Compound (*Z*)-3-benzylidenebenzofuran-2(3H)-one (**1a**) (1 equiv, 0.2 mmol), sarcosine (3 equiv, 0.6 mmol), and ninhydrin (2 equiv, 0.4 mmol) were sequentially added into a round-bottom flask. Then, 2 mL of tetrahydrofuran was added to dissolve them. The reaction was stirred at 60 °C and monitored by TLC. After that, the solvent was evaporated. The yellow solid compound 4a was purified through silica gel column chromatography. Compounds 4b–4x were prepared by a similar method.

### 3.3. Characterization Data

Product **4a** was a yellow solid in 99% yield, Mp: 179.0–180.5 °C, mobile phase: PE/EA = 5:1; ^1^H NMR (400 MHz, CDCl_3_) δ 8.06–8.04 (m, 1H), 7.92–7.86 (m, 2H), 7.78–7.74 (m, 1H), 7.71 (dd, *J* = 7.7, 1.1 Hz, 1H), 7.22 (td, *J* = 7.6, 1.2 Hz, 1H), 7.18–7.13 (m, 1H), 7.06–6.98 (m, 5H), 6.67–6.65 (m, 1H), 4.89 (t, *J* = 10.1 Hz, 1H), 4.02 (t, *J* = 9.2 Hz, 1H), 3.81 (t, *J* = 9.7 Hz, 1H), 2.52 (s, 3H); ^13^C NMR (100 MHz, CDCl_3_) δ 199.8, 196.0, 174.1, 152.4, 141.0, 140.8, 137.1, 136.0, 134.2, 129.8, 128.9, 128.5, 128.2, 127.8, 123.8, 123.7, 123.4, 123.3, 110.3, 80.4, 65.4, 55.9, 53.5, 35.8; IR (ATR): 3058, 3029, 2967, 2940, 2876, 2787, 1964, 1794, 1739, 1704, 1616, 1592, 1461, 1328, 1286, 1245, 1137, 1085, 874, 757, 704, 637, 581, 514, 463, 450 cm^−1^; HRMS (ESI): Exact mass calcd for C_26_H_20_NO_4_ [M+H]^+^: 410.1392, Found: 410.1387.

Product **4b** was a yellow solid in 86% yield, Mp: 171.5–173.0 °C, mobile phase: PE/EA = 4:1; ^1^H NMR (400 MHz, CDCl_3_) δ 7.94 (dt, *J* = 7.7, 0.9 Hz, 1H), 7.76 (td, *J* = 7.5, 1.1 Hz, 1H), 7.66 (td, *J* = 7.5, 1.1 Hz, 1H), 7.54 (dt, *J* = 7.7, 0.9 Hz, 1H), 7.37 (dt, *J* = 7.8, 1.1 Hz, 1H), 7.05 (td, *J* = 7.8, 1.6 Hz, 1H), 6.93 (td, *J* = 7.8, 1.5 Hz, 1H), 6.86–6.80 (m, 2H), 6.71 (td, *J* = 7.6, 1.1 Hz, 1H), 6.63 (dd, *J* = 8.0, 1.0 Hz, 1H), 6.45 (dd, *J* = 8.2, 1.1 Hz, 1H), 4.84 (t, *J* = 8.8 Hz, 1H), 4.30 (t, *J* = 8.9 Hz, 1H), 3.83 (t, *J* = 8.9 Hz, 1H), 3.34 (s, 3H), 2.52 (s, 3H); ^13^C NMR (100 MHz, CDCl_3_) δ 198.1, 198.0, 175.0, 157.2, 152.9, 141.4, 141.0, 136.5, 136.1, 129.0, 128.4, 128.2, 127.7, 125.0, 124.2, 123.2, 122.9, 122.8, 120.0, 109.6, 109.4, 81.2, 63.1, 55.7, 54.2, 46.3, 36.0; IR (ATR): 3092, 3070, 2941, 2874, 2832, 2805, 1817, 1735, 1698, 1590, 1462, 1238, 1129, 1073, 1029, 923, 760, 745, 580, 508, 469 cm^−1^; HRMS (ESI): Exact mass calcd for C_27_H_21_NNaO_5_ [M+Na]^+^: 462.1317, Found: 462.1312.

Product **4c** was a yellow solid in 99% yield, Mp: 154.0–155.5 °C, mobile phase: PE/EA = 4:1; ^1^H NMR (400 MHz, CDCl_3_) δ 8.04 (d, *J* = 7.7 Hz, 1H), 7.92–7.86 (m, 2H), 7.76 (td, *J* = 7.5, 1.1 Hz, 1H), 7.70 (d, *J* = 7.6 Hz, 1H), 7.23 (td, *J* = 7.6, 1.3 Hz, 1H), 7.17 (td, *J* = 7.8, 1.7 Hz, 1H), 6.95 (t, *J* = 7.9 Hz, 1H), 6.70 (dd, *J* = 7.9, 1.2 Hz, 1H), 6.61–6,58 (m, 2H), 6.50 (t, *J* = 2.1 Hz, 1H), 4.86 (t, *J* = 9.4 Hz, 1H), 3.98 (t, *J* = 9.2 Hz, 1H), 3.80 (t, *J* = 9.7 Hz, 1H), 3.59 (s, 3H), 2.51 (s, 3H); ^13^C NMR (100 MHz, CDCl_3_) δ 199.8, 196.0, 174.0, 159.2, 152.4, 141.0, 140.8, 137.1, 136.1, 135.7, 129.8, 129.2, 128.8, 123.7, 123.6, 123.4, 123.3, 121.0, 113.8, 113.7, 110.4, 80.4, 65.2, 56.0, 55.2, 53.4, 35.8; IR (ATR): 2953, 2920, 2864, 2800, 1793, 1746, 1706, 1607, 1594, 1579, 1461, 1443, 1290, 1238, 1160, 1138, 1082, 1053, 774, 754, 693, 634, 605, 486, 457 cm^−1^; HRMS (ESI): Exact mass calcd for C_27_H_21_NNaO_5_ [M+Na]^+^: 440.1498, Found: 440.1492.

Product **4d** was a yellow solid in 74% yield, Mp: 134.6–136.0 °C, mobile phase: PE/EA = 4:1; ^1^H NMR (400 MHz, CDCl_3_) δ 8.04 (dt, *J* = 7.7, 1.0 Hz, 1H), 7.92–7.86 (m, 2H), 7.76 (td, *J* = 7.4, 1.1 Hz, 1H), 7.70 (dt, *J* = 7.7, 1.1 Hz, 1H), 7.23 (td, *J* = 7.6, 1.3 Hz, 1H), 7.17 (td, *J* = 7.8, 1.7 Hz, 1H), 6.90–6.89 (m, 2H), 6.69 (dd, *J* = 7.7, 1.3 Hz, 1H), 6.58–6.55 (m, 2H), 4.84 (t, *J* = 9.5 Hz, 1H), 3.94 (t, *J* = 9.3 Hz, 1H), 3.77 (t, *J* = 9.7 Hz, 1H), 3.64 (s, 3H), 2.50 (s, 3H); ^13^C NMR (100 MHz, CDCl_3_) δ 199.8, 196.1, 174.1, 159.1, 152.4, 141.0, 140.8, 137.1, 136.0, 129.8, 129.7, 128.8, 126.0, 123.8, 123.7, 123.4, 123.3, 113.6, 110.4, 80.4, 65.4, 56.2, 55.2, 53.1, 35.8; IR (ATR): 3068, 3003, 2938, 2864, 2838, 2805, 2257, 1793, 1742, 1705, 1595, 1514, 1463, 1250, 1180, 1072, 1035, 769, 729, 691, 613, 530, 473, 446 cm^−1^; HRMS (ESI): Exact mass calcd for C_27_H_21_NNaO_5_ [M+Na]^+^: 462.1317, Found: 462.1312.

Product **4e** was a yellow solid in 86% yield, Mp: 172.0–173.0 °C, mobile phase: PE/EA = 5:1; ^1^H NMR (400 MHz, CDCl_3_) δ 8.05 (dt, *J* = 7.8, 1.0 Hz, 1H), 7.93–7.87 (m, 2H), 7.76 (td, *J* = 7.4, 1.1 Hz, 1H), 7.71 (dt, *J* = 7.8, 1.0 Hz, 1H), 7.22 (td, *J* = 7.6, 1.2 Hz, 1H), 7.16 (td, *J* = 7.8, 1.6 Hz, 1H), 6.92 (t, *J* = 7.5 Hz, 1H), 6.86 (d, *J* = 7.6 Hz, 1H), 6.80–6.75 (m, 2H), 6.68 (dd, *J* = 7.9, 1.2 Hz, 1H), 4.84 (t, *J* = 9.4 Hz, 1H), 3.99 (t, *J* = 9.2 Hz, 1H), 3.79 (t, *J* = 9.7 Hz, 1H), 2.51 (s, 3H), 2.12 (s, 3H); ^13^C NMR (100 MHz, CDCl_3_) δ 199.8, 196.1, 174.1, 152.4, 141.0, 140.9, 137.8, 137.1, 136.0, 134.1, 129.7, 129.5, 129.0, 128.6, 128.0, 125.5, 123.8, 123.6, 123.4, 123.3, 110.3, 80.5, 65.4, 56.1, 53.5, 35.9, 21.3; IR (ATR): 3034, 2966, 2938, 2912, 2870, 2797, 1792, 1741, 1706, 1593, 1461, 1328, 1265, 1239, 1137, 1081, 1065, 1037, 1016, 977, 939, 870, 752, 712, 693, 615, 589, 472, 445 cm^−1^; HRMS (ESI): Exact mass calcd for C_27_H_21_NNaO_4_ [M+Na]^+^: 446.1368, Found: 446.1363.

Product **4f** was a yellow solid in 96% yield, Mp: 164.3–165.5 °C, mobile phase: PE/EA = 5:1; ^1^H NMR (400 MHz, CDCl_3_) δ 7.91–7.88 (m, 1H), 7.80–7.76 (m, 2H), 7.73–7.69 (m, 1H), 7.18 (dd, *J* = 7.6, 1.3 Hz, 1H), 7.02 (td, *J* = 7.8, 1.4 Hz, 1H), 6.97–6.95 (m, 2H), 6.92–6.87 (m, 3H), 6.70–6.68 (m, 1H), 4.73 (t, *J* = 9.2 Hz, 1H), 4.37 (t, *J* = 9.1 Hz, 1H), 3.63 (t, *J* = 9.5 Hz, 1H), 2.48 (s, 3H), 2.18 (s, 3H); ^13^C NMR (100 MHz, CDCl_3_) δ 201.6, 197.9, 170.5, 153.2, 141.4, 140.4, 137.5, 136.8, 136.6, 131.2, 130.0, 129.2, 128.7, 124.5, 123.9, 123.4, 122.8, 122.5, 111.0, 80.4, 63.9, 56.9, 50.5, 36.8, 21.0; IR (ATR): 3062, 3041, 2939, 2853, 2793, 1811, 1739, 1703, 1593, 1478, 1464, 1266, 1250, 1230, 1130, 1029, 979, 917, 776, 757, 678, 598, 565, 546, 514, 470 cm^−1^; HRMS (ESI): Exact mass calcd for C_27_H_22_NO_4_ [M+H]^+^: 424.1549, Found: 424.1543.

Product **4g** was a yellow solid in 88% yield, Mp: 176.9–178.2 °C, mobile phase: PE/EA = 4:1; ^1^H NMR (400 MHz, CDCl_3_) δ 8.02 (dt, *J* = 7.6, 0.9 Hz, 1H), 7.86 (td, *J* = 7.5, 1.2 Hz, 1H), 7.74 (td, *J* = 7.5, 1.1 Hz, 1H), 7.67 (dt, *J* = 7.7, 0.9 Hz, 1H), 7.61 (dd, *J* = 7.4, 1.7 Hz, 1H), 7.14–7.02 (m, 4H), 6.88–6.78 (m, 2H), 6.70 (dd, *J* = 7.6, 1.3 Hz, 1H), 5.15 (t, *J* = 9.6 Hz, 1H), 4.07 (t, *J* = 8.8 Hz, 1H), 3.86 (t, *J* = 9.6 Hz, 1H), 2.51 (s, 3H); ^13^C NMR (100 MHz, CDCl_3_) δ 199.2, 196.5, 173.9, 161.1 (d, *J* = 247.7 Hz), 152.7, 141.1, 141.0, 137.0, 136.1, 129.8, 129.6 (d, *J* = 3.7 Hz), 129.2 (d, *J* = 8.5 Hz), 128.8, 123.8, 123.7, 123.6, 123.4 (d, *J* = 4.5 Hz), 123.2, 122.3 (d, *J* = 13.8 Hz), 115.5 (d, *J* = 22.6 Hz), 110.2, 80.3, 63.8, 55.7, 45.7 (d, *J* = 2.1 Hz), 35.8; ^19^F NMR (376 MHz, CDCl_3_) δ-113.41; IR (ATR): 3098, 3075, 2972, 2945, 2918, 2872, 2800, 1799, 1743, 1708, 1618, 1594, 1493, 1460, 1327, 1284, 1238, 1134, 1077, 1065, 1005, 932, 767, 750, 691, 632, 617, 584, 488, 449 cm^−1^; HRMS (ESI): Exact mass calcd for C_26_H_18_FNNaO_4_ [M+Na]^+^: 450.1118, Found: 450.1112.

Product **4h** was a yellow solid in 92% yield, Mp: 197.6–199.0 °C, mobile phase: PE/EA = 5:1; ^1^H NMR (400 MHz, CDCl_3_) δ 7.90 (d, *J* = 7.5 Hz, 1H), 7.82–7.70 (m, 3H), 7.17 (d, *J* = 7.6 Hz, 1H), 7.10–7.03 (m, 2H), 6.90 (t, *J* = 7.6 Hz, 1H), 6.85–6.76 (m, 3H), 6.72 (d, *J* = 8.0 Hz, 1H), 4.77 (t, *J* = 9.2 Hz, 1H), 4.34 (t, *J* = 9.0 Hz, 1H), 3.65 (t, *J* = 9.5 Hz, 1H), 2.47 (s, 3H); ^13^C NMR (100 MHz, CDCl_3_) δ 201.6, 197.7, 170.4, 162.5 (d, *J* = 245.7 Hz), 153.2, 141.4, 140.4, 137.0 (d, *J* = 7.4 Hz), 136.8, 136.7, 130.4, 129.9 (d, *J* = 8.1 Hz), 124.5 (d, *J* = 2.9 Hz), 124.4, 124.1, 123.5, 122.8, 122.1, 115.9 (d, *J* = 22.0 Hz), 114.9 (d, *J* = 20.9 Hz), 111.2, 80.2, 63.5, 56.7, 50.1, 36.8; ^19^F NMR (376 MHz, CDCl_3_) δ-112.57; IR (ATR): 3089, 3069, 2951, 2864, 2783, 1807, 1742, 1705, 1614, 1588, 1487, 1459, 1293, 1278, 1226, 1125, 1062, 1039, 985, 876, 764, 680, 596, 522, 478, 431 cm^−1^; HRMS (ESI): Exact mass calcd for C_26_H_19_FNO_4_ [M+H]^+^: 428.1298, Found: 428.1293.

Product **4i** was a yellow solid in 92% yield, Mp: 191.5–192.3 °C, mobile phase: PE/EA = 5:1; ^1^H NMR (400 MHz, CDCl_3_) δ 7.90 (dt, *J* = 7.4, 1.1 Hz, 1H), 7.82–7.77 (m, 2H), 7.74–7.70 (m, 1H), 7.17 (dd, *J* = 7.6, 1.3 Hz, 1H), 7.07–7.02 (m, 3H), 6.90 (td, *J* = 7.6, 1.1 Hz, 1H), 6.82–6.77 (m, 2H), 6.71 (dd, *J* = 8.1, 1.0 Hz, 1H), 4.75 (t, *J* = 9.2 Hz, 1H), 4.33 (t, *J* = 9.1 Hz, 1H), 3.64 (t, *J* = 9.5 Hz, 1H), 2.47 (s, 3H); ^13^C NMR (100 MHz, CDCl_3_) δ 201.6, 197.8, 170.5, 162.4 (d, *J* = 246.9 Hz), 153.2, 141.4, 140.4, 136.8 (d, *J* = 24.0 Hz) 130.5 (d, *J* = 8.0 Hz), 130.3, 130.0 (d, *J* = 3.2 Hz), 124.4, 124.0, 123.5, 122.8, 122.2, 115.5, 115.3, 111.1, 80.2, 63.8, 57.0, 50.1, 36.8; ^19^F NMR (376 MHz, CDCl_3_) δ-114.36; IR (ATR): 3063, 3038, 2948, 2850, 2785, 1811, 1739, 1705, 1592, 1511, 1478, 1478, 1354, 1328, 1267, 1231, 1207, 1163, 1125, 1062, 1039, 983, 926, 843, 828, 769, 679, 602, 566, 543, 518, 476 cm^−1^; HRMS (ESI): Exact mass calcd for C_26_H_18_FNNaO_4_ [M+Na]^+^: 450.1118, Found: 450.1112.

Product **4j** was a yellow solid in 87% yield, Mp: 176.0–177.0 °C, mobile phase: PE/EA = 4:1; ^1^H NMR (400 MHz, CDCl_3_) δ 7.95 (dt, *J* = 7.7, 1.0 Hz, 1H), 7.79 (td, *J* = 7.4, 1.2 Hz, 1H), 7.71 (td, *J* = 7.5, 1.1 Hz, 1H), 7.63 (dt, *J* = 7.6, 1.0 Hz, 1H), 7.48 (dd, *J* = 7.9, 1.7 Hz, 1H), 7.14–7.12 (m, 1H), 7.10–7.08 (m, 2H), 7.04 (td, *J* = 7.7, 1.6 Hz, 2H), 6.86 (td, *J* = 7.6, 1.1 Hz, 1H), 6.69 (dd, *J* = 8.1, 1.0 Hz, 1H), 5.24 (t, *J* = 8.7 Hz, 1H), 4.06 (t, *J* = 8.1 Hz, 1H), 3.92 (t, *J* = 9.3 Hz, 1H), 2.50 (s, 3H); ^13^C NMR (100 MHz, CDCl_3_) δ 198.2, 198.0, 174.4, 153.1, 141.3, 141.0, 136.8, 136.2, 135.8, 133.6, 129.9, 129.7, 129.6, 128.7, 128.6, 126.3, 123.4, 123.2, 123.1, 123.0, 110.3, 80.6, 63.2, 57.4, 48.2, 35.9; IR (ATR): 3068, 2979, 2933, 2879, 2798, 1796, 1742, 1708, 1593, 1461, 1349, 1280, 1237, 1237, 1131, 1077, 1045, 991, 991, 968, 937, 859, 767, 735, 689, 634, 578, 530, 488, 423 cm^−1^; HRMS (ESI): Exact mass calcd for C_26_H_18_ClNNaO_4_ [M+Na]^+^: 466.0816, Found: 466.0818.

Product **4k** was a yellow solid in 90% yield, Mp: 183.1–184.0 °C, mobile phase: PE/EA = 5:1; ^1^H NMR (400 MHz, CDCl_3_) δ 7.90 (dt, *J* = 7.6, 1.0 Hz, 1H), 7.81–7.76 (m, 2H), 7.74–7.70 (m, 1H), 7.17 (dd, *J* = 7.8, 1.3 Hz, 1H), 7.10–7.02 (m, 4H), 6.96 (dt, *J* = 7.6, 1.6 Hz, 1H), 6.91 (td, *J* = 7.7, 1.1 Hz, 1H), 6.73–6.71 (m, 1H), 4.74 (t, *J* = 9.2 Hz, 1H), 4.32 (t, *J* = 9.1 Hz, 1H), 3.64 (t, *J* = 9.5 Hz, 1H), 2.46 (s, 3H); ^13^C NMR (100 MHz, CDCl_3_) δ 201.6, 197.6, 170.4, 153.2, 141.4, 140.4, 137.0, 136.7, 136.6, 134.2, 130.4, 129.7, 129.1, 128.1, 126.9, 124.5, 124.1, 123.5, 122.8, 122.1, 111.2, 80.2, 63.5, 56.7, 50.0, 36.8; IR (ATR): 3094, 2945, 2865, 2798, 1809, 1734, 1701, 1572, 1480, 1464, 1430, 1342, 1297, 1261, 1228, 1183, 1129, 1031, 984, 877, 779, 748, 686, 594, 582, 528, 470, 440 cm^−1^; HRMS (ESI): Exact mass calcd for C_26_H_18_ClNNaO_4_ [M+Na]^+^: 466.0822, Found: 466.0816.

Product **4l** was a yellow solid in 96% yield, Mp: 171.5–172.0 °C, mobile phase: PE/EA = 5:1; ^1^H NMR (400 MHz, CDCl_3_) δ 8.05 (dt, *J* = 7.7, 1.0 Hz, 1H), 7.92–7.87 (m, 2H), 7.77 (td, *J* = 7.4, 1.1 Hz, 1H), 7.71 (dt, *J* = 7.6, 1.0 Hz, 1H), 7.25–7.18 (m, 2H), 7.03–7.00 (m, 2H), 6.93–6.91 (m, 2H), 6.73–6.70 (m, 1H), 4.85 (t, *J* = 9.4 Hz, 1H), 3.94 (t, *J* = 9.3 Hz, 1H), 3.80 (t, *J* = 9.7 Hz, 1H), 2.50 (s, 3H); ^13^C NMR (100 MHz, CDCl_3_) δ 199.7, 195.8, 173.9, 152.4, 141.0, 140.8, 137.2, 136.2, 133.8, 132.8, 130.0, 129.9, 128.7, 128.4, 123.8, 123.6, 123.5, 123.4, 110.5, 80.3, 65.2, 55.9, 52.8, 35.8; IR (ATR): 3099, 3074, 2974, 2942, 2868, 2803, 1790, 1709, 1740, 1594, 1476, 1463, 1496, 1411, 1327, 1284, 1266, 1237, 1141, 1085, 1014, 972, 872, 832, 756, 690, 613, 583, 520, 468, 435 cm^−1^; HRMS (ESI): Exact mass calcd for C_26_H_18_ClNNaO_4_ [M+Na]^+^: 466.0822, Found: 466.0811.

Product **4m** was a yellow solid in 90% yield, Mp: 157.9–159.7 °C, mobile phase: PE/EA = 4:1; ^1^H NMR (400 MHz, CDCl_3_) δ 7.94 (dt, *J* = 7.7, 0.9 Hz, 1H), 7.77 (td, *J* = 7.4, 1.2 Hz, 1H), 7.70 (td, *J* = 7.4, 1.1 Hz, 1H), 7.62 (dt, *J* = 7.6, 1.0 Hz, 1H), 7.54 (dd, *J* = 8.0, 1.6 Hz, 1H), 7.33 (dd, *J* = 8.0, 1.4 Hz, 1H), 7.16 (td, *J* = 7.7, 1.3 Hz, 1H), 7.06–6.95 (m, 3H), 6.83 (td, *J* = 7.7, 1.1 Hz, 1H), 6.70 (d, *J* = 8.0 Hz, 1H), 5.21 (t, *J* = 8.8 Hz, 1H), 4.02 (t, *J* = 9.6 Hz, 1H), 3.94 (t, *J* = 9.2 Hz, 1H), 2.50 (s, 3H); ^13^C NMR (100 MHz, CDCl_3_) δ 198.2, 198.0, 174.4, 153.2, 141.3, 141.0, 136.7, 136.2, 135.4, 133.0, 130.3, 129.8, 129.0, 128.5, 127.1, 126.9, 123.4, 123.2, 123.1, 122.9, 110.4, 80.7, 63.1, 57.9, 50.5, 35.9; IR (ATR): 3087, 2917, 2878, 2802, 1810, 1736, 1702, 1633, 1619, 1590, 1464, 1355, 1330, 1262, 1235, 1183, 1160, 1131, 1072, 1026, 972, 916, 874, 762, 574, 530, 474 cm^−1^; HRMS (ESI): Exact mass calcd for C_26_H_18_BrNNaO_4_ [M+Na]^+^: 510.0311, Found: 510.0304.

Product **4n** was a yellow solid in 96% yield, Mp: 164.5–166.0 °C, mobile phase: PE/EA = 5:1; ^1^H NMR (400 MHz, CDCl_3_) δ 8.06–8.04 (m, 1H), 7.92–7.88 (m, 2H), 7.78 (td, *J* = 7.4, 1.1 Hz, 1H), 7.72 (dt, *J* = 7.7, 1.0 Hz, 1H), 7.28–7.13 (m, 4H), 6.95–6.92 (m, 2H), 6.73 (dd, *J* = 7.8, 1.2 Hz, 1H), 4.84 (t, *J* = 9.4 Hz, 1H), 3.94 (t, *J* = 9.2 Hz, 1H), 3.82 (t, *J* = 9.7 Hz, 1H), 2.51 (s, 3H); ^13^C NMR (100 MHz, CDCl_3_) δ 199.7, 195.8, 173.8, 152.4, 141.0, 140.8, 137.2, 136.7, 136.2, 131.6, 131.0, 130.1, 129.8, 128.8, 127.4, 123.8, 123.5, 123.4, 123.3, 122.3, 110.5, 80.2, 65.1, 55.9, 53.0, 35.8; IR (ATR): 3068, 2967, 2908, 2866, 2796, 1792, 1742, 1709, 1618, 1593, 1567, 1474, 1462, 1263, 1239, 1138, 1081, 979, 863, 792, 771, 750, 633, 615, 590, 472, 440 cm^−1^; HRMS (ESI): Exact mass calcd for C_26_H_18_BrNNaO_4_ [M+Na]^+^: 510.0317, Found: 510.0311.

Product **4o** was a yellow solid in 99% yield, Mp: 177.6–179.4 °C, mobile phase: PE/EA = 4:1; ^1^H NMR (400 MHz, CDCl_3_) δ 8.04 (dt, *J* = 7.7, 1.0 Hz, 1H), 7.91–7.87 (m, 2H), 7.77 (td, *J* = 7.4, 1.1 Hz, 1H), 7.71 (dt, *J* = 7.7, 1.0 Hz, 1H), 7.26–7.19 (m, 2H), 7.18–7.15 (m, 2H), 6.87–6.84 (m, 2H), 6.73–6.70 (m, 1H), 4.83 (t, *J* = 9.4 Hz, 1H), 3.94 (t, *J* = 9.2 Hz, 1H), 3.80 (t, *J* = 9.7 Hz, 1H), 2.50 (s, 3H); ^13^C NMR (100 MHz, CDCl_3_) δ 199.7, 195.8, 173.8, 152.4, 141.0, 140.7, 137.2, 136.1, 133.3, 131.4, 130.2, 130.1, 128.7, 123.8, 123.4, 123.3, 122.0, 110.6, 80.3, 65.0, 55.9, 52.8, 35.8; IR (ATR): 3099, 3070, 2974, 2941, 2869, 2802, 1790, 1739, 1709, 1594, 1492, 1463, 1303, 1266, 1237, 1132, 1085, 1043, 1009, 970, 934, 872, 816, 757, 690, 612, 582, 514, 466, 421 cm^−1^; HRMS (ESI): Exact mass calcd for C_26_H_18_BrNNaO_4_ [M+Na]^+^: 510.0317, Found: 510.0311.

Product **4p** was a yellow solid in 83% yield, Mp: 178.0–179.0 °C, mobile phase: PE/EA = 3:1; ^1^H NMR (400 MHz, CDCl_3_) δ 8.06 (dt, *J* = 7.7, 1.0 Hz, 1H), 7.94–7.90 (m, 4H), 7.80 (td, *J* = 7.5, 1.1 Hz, 1H), 7.73 (dt, *J* = 7.7, 1.0 Hz, 1H), 7.28–7.18 (m, 4H), 6.73–6.70 (m, 1H), 4.99 (t, *J* = 9.3 Hz, 1H), 4.03 (t, *J* = 9.1 Hz, 1H), 3.89 (t, *J* = 9.7 Hz, 1H), 2.53 (s, 3H); ^13^C NMR (100 MHz, CDCl_3_) δ 199.6, 195.4, 173.6, 152.2, 147.4, 142.0, 141.0, 140.6, 137.3, 136.3, 130.4, 129.5, 128.7, 124.0, 123.6, 123.5, 123.4, 122.9, 110.6, 80.0, 64.9, 55.6, 52.8, 35.8; IR (ATR): 3438, 3072, 2966, 2937, 2867, 2800, 1794, 1741, 1706, 1598, 1522, 1465, 1349, 1241, 1136, 1078, 1010, 974, 937, 861, 766, 698, 579, 519, 464, 436 cm^−1^; HRMS (ESI): Exact mass calcd for C_26_H_18_N_2_NaO_6_ [M+Na]^+^: 477.1063, Found: 477.1057.

Product **4q** was a yellow solid in 88% yield, Mp: 188.3–189.5 °C, mobile phase: PE/EA = 2:1; ^1^H NMR (400 MHz, CDCl_3_) δ 12.80 (dt, *J* = 7.8, 1.0 Hz, 1H), 12.68–12.62 (m, 2H), 12.54 (td, *J* = 7.4, 1.1 Hz, 1H), 12.47 (dt, *J* = 7.8, 1.0 Hz, 1H), 12.11–12.08 (m, 2H), 12.00–11.93 (m, 2H), 11.87–11.85 (m, 2H), 11.47–11.45 (m, 1H), 9.67 (t, *J* = 9.3 Hz, 1H), 8.74 (t, *J* = 9.1 Hz, 1H), 8.60 (t, *J* = 9.7 Hz, 1H), 7.26 (s, 3H); ^13^C NMR (100 MHz, CDCl_3_) δ 199.6, 195.5, 173.6, 152.2, 140.9, 140.6, 140.0, 137.3, 136.3, 132.0, 130.3, 129.3, 128.7, 124.0, 123.5, 123.4, 123.0, 118.4, 111.8, 110.6, 80.0, 64.9, 55.5, 53.1, 35.7; IR (ATR): 3074, 2927, 2864, 2227, 1798, 1742, 1704, 1594, 1505, 1462, 1328, 1267, 1238, 1140, 1082, 1037, 1017, 977, 934, 873, 850, 771, 633, 614, 586, 554, 440 cm^−1^; HRMS (ESI): Exact mass calcd for C_27_H_18_N_2_NaO_4_ [M+Na]^+^: 457.1164, Found: 457.1159.

Product **4r** was a yellow solid in 99% yield, Mp: 175.0–176.5 °C, mobile phase: PE/EA = 4:1; ^1^H NMR (400 MHz, CDCl_3_) δ 7.91–7.87 (m, 1H), 7.80–7.75 (m, 2H), 7.72–7.68 (m, 1H), 7.19 (dd, *J* = 7.7, 1.3 Hz, 1H), 7.02 (td, *J* = 7.8, 1.4 Hz, 1H), 6.91–6.84 (m, 3H), 6.76 (dd, *J* = 7.9, 1.9 Hz, 1H), 6.69 (dd, *J* = 8.1, 1.1 Hz, 1H), 4.71 (t, *J* = 9.2 Hz, 1H), 4.36 (t, *J* = 9.1 Hz, 1H), 3.63 (t, *J* = 9.4 Hz, 1H), 2.49 (s, 3H), 2.09 (s, 3H), 2.05 (s, 3H). ^13^C NMR (100 MHz, CDCl_3_) δ 201.6, 197.8, 170.5, 153.3, 141.4, 140.5, 136.8, 136.6, 136.5, 136.2, 131.7, 130.1, 130.0, 129.6, 126.2, 124.6, 123.8, 123.4, 122.8, 122.7, 110.9, 80.5, 63.8, 57.0, 50.4, 36.9, 19.8, 19.4; IR (ATR): 3067, 2965, 2921, 2851, 2794, 1813, 1737, 1702, 1616, 1590, 1506, 1477, 1460, 1354, 1329, 1289, 1231, 1131, 1071, 1035, 982, 921, 879, 817, 758, 659, 635, 585, 491, 478, 432 cm^−1^; HRMS (ESI): Exact mass calcd for C_28_H_24_NO_4_ [M+H]^+^: 438.1699, Found: 438.1698.

Product **4s** was a yellow solid in 91% yield, Mp: 187.5–189.0 °C, mobile phase: PE/EA = 6:1; ^1^H NMR (400 MHz, CDCl_3_) δ 8.05 (dt, *J* = 7.7, 0.9 Hz, 1H), 7.93 (dd, *J* = 7.5, 1.5 Hz, 1H), 7.89 (td, *J* = 7.4, 1.3 Hz, 1H), 7.76 (td, *J* = 7.4, 1.1 Hz, 1H), 7.71 (dt, *J* = 7.7, 1.0 Hz, 1H), 7.23 (td, *J* = 7.6, 1.2 Hz, 1H), 7.17 (td, *J* = 7.8, 1.6 Hz, 1H), 6.70–6.68 (m, 2H), 6.58 (s, 2H), 4.79 (t, *J* = 9.4 Hz, 1H), 3.97 (t, *J* = 9.2 Hz, 1H), 3.77 (t, *J* = 9.7 Hz, 1H), 2.50 (s, 3H), 2.07 (s, 6H); ^13^C NMR (100 MHz, CDCl_3_) δ 199.8, 196.2, 174.1, 152.5, 141.0, 140.8, 137.6, 137.1, 136.0, 134.0, 129.7, 129.4, 129.0, 126.5, 123.9, 123.5, 123.4, 123.3, 110.3, 80.4, 65.4, 56.2, 53.5, 35.8, 21.2; IR (ATR): 3088, 2966, 2910, 2869, 2795, 1791, 1741, 1705, 1593, 1461, 1402, 1378, 1327, 1268, 1237, 1139, 1079, 1038, 976, 936, 902, 856, 774, 751, 708, 688, 618, 593, 472, 437 cm^−1^; HRMS (ESI): Exact mass calcd for C_28_H_23_NNaO_4_ [M+Na]^+^: 460.1525, Found: 460.1519.

Product **4t** was a yellow solid in 83% yield, Mp: 157.6–159.0 °C, mobile phase: PE/EA = 2:1; ^1^H NMR (400 MHz, CDCl_3_) δ 7.94 (dt, *J* = 7.7, 0.9 Hz, 1H), 7.76 (td, *J* = 7.5, 1.1 Hz, 1H), 7.66 (td, *J* = 7.4, 1.1 Hz, 1H), 7.54 (dt, *J* = 7.7, 1.0 Hz, 1H), 6.99–6.94 (m, 2H), 6.87 (dd, *J* = 7.7, 1.4 Hz, 1H), 6.78 (td, *J* = 7.6, 1.1 Hz, 1H), 6.64 (dd, *J* = 8.0, 0.9 Hz, 1H), 6.49 (d, *J* = 8.8 Hz, 1H), 4.79 (m, 1H), 4.17 (t, *J* = 9.2 Hz, 1H), 3.81 (t, *J* = 8.9 Hz, 1H), 3.76 (s, 3H), 3.61 (s, 3H), 3.36 (s, 3H), 2.51 (s, 3H); ^13^C NMR (100 MHz, CDCl_3_) δ 197.9, 197.8, 175.0, 153.2, 153.0, 152.0, 141.6, 141.3, 140.9, 136.4, 136.0, 129.0, 128.4, 124.1, 123.2, 122.8, 122.7, 122.2, 122.1, 109.7, 106.1, 81.4, 63.4, 60.4, 59.7, 56.2, 55.9, 46.1, 35.9; IR (ATR): 3064, 3000, 2930, 2857, 2799, 1801, 1744, 1708, 1618, 1595, 1498, 1463, 1416, 1299, 1101, 1077, 978, 941, 896, 862, 805, 754, 689, 645, 601, 471, 451 cm^−1^; HRMS (ESI): Exact mass calcd for C_29_H_25_NNaO_7_ [M+Na]^+^: 522.1529, Found: 522.1523.

Product **4u** was a yellow solid in 88% yield, Mp: 173.9–175.5 °C, mobile phase: PE/EA = 6:1; ^1^H NMR (400 MHz, CDCl_3_) δ 8.03 (dd, *J* = 7.8, 1.0 Hz, 1H), 7.95–7.92 (m, 1H), 7.86 (td, *J* = 7.5, 1.2 Hz, 1H), 7.74 (td, *J* = 7.5, 1.1 Hz, 1H), 7.68 (dt, *J* = 7.8, 1.0 Hz, 1H), 7.16–7.10 (m, 2H), 7.05–6.98 (m, 1H), 6.69–6.60 (m, 3H), 5.20 (dd, *J* = 10.5, 7.3 Hz, 1H), 4.35 (dd, *J* = 8.8, 7.3 Hz, 1H), 3.88–3.83 (m 1H), 2.49 (s, 3H); ^13^C NMR (100 MHz, CDCl_3_) δ 199.7, 195.8, 173.8, 161.6 (dd, J = 250.2, 7.7 Hz), 152.6, 141.3, 141.2, 137.0, 136.0, 129.9, 129.7 (t, J = 10.2 Hz), 129.6, 129.5, 123.5, 123.4, 123.3, 111.8 (dd, J = 25.3, 2.5 Hz), 111.7 (t, J = 20.2 Hz), 109.6, 79.2, 64.2, 53.7, 44.4, 35.7; ^19^F NMR (377 MHz, CDCl_3_) δ-106.62; IR (ATR): 3103, 3067, 2971, 2939, 2888, 2864, 2848, 2796, 1793, 1741, 1706, 1623, 1594, 1463, 1402, 1349, 1283, 1237, 1135, 1086, 971, 792, 773, 759, 727, 689, 634, 546, 518, 493, 464 cm^−1^; HRMS (ESI): Exact mass calcd for C_26_H_18_F_2_NO_4_ [M+H]^+^: 446.1198, Found: 446.1192.

Product **4v** was a yellow solid in 75% yield, Mp: 188.5–189.5 °C, mobile phase: PE/EA = 5:1; ^1^H NMR (400 MHz, CDCl_3_) δ 7.90 (dt, *J* = 7.6, 1.1 Hz, 1H), 7.82–7.77 (m, 2H), 7.75–7.71 (m, 1H), 7.19–7.14 (m, 3H), 7.07 (td, *J* = 7.9, 1.4 Hz, 1H), 6.94–6.90 (m, 2H), 6.76–6.74 (m, 1H), 4.72 (t, *J* = 9.2 Hz, 1H), 4.28 (t, *J* = 9.0 Hz, 1H), 3.65 (t, *J* = 9.6 Hz, 1H), 2.46 (s, 3H); ^13^C NMR (100 MHz, CDCl_3_) δ 201.5, 197.5, 170.4, 153.2, 141.4, 140.4, 137.0, 136.8, 134.9, 132.4, 132.1, 130.9, 130.5, 130.4, 128.1, 124.4, 124.2, 123.6, 122.8, 121.8, 111.3, 80.1, 63.4, 56.7, 49.5, 36.8; IR (ATR): 3076, 2932, 2869, 2798, 1812, 1738, 1701, 1591, 1478, 1463, 1358, 1328, 1232, 1132, 1031, 985, 921, 880, 846, 821, 782, 764, 696, 592, 494, 478, 437 cm^−1^; HRMS (ESI): Exact mass calcd for C_26_H_18_Cl_2_NO_4_ [M+H]^+^: 478.0613, Found: 478.0601.

Product **4w** was a yellow solid in 92% yield, Mp: 196.5–198.0 °C, mobile phase: PE/EA = 5:1; ^1^H NMR (400 MHz, CDCl_3_) δ 8.05 (dt, *J* = 7.7, 1.0 Hz, 1H), 7.93–7.89 (m, 2H), 7.79 (td, *J* = 7.5, 1.1 Hz, 1H), 7.73 (dt, *J* = 7.7, 1.0 Hz, 1H), 7.30–7.22 (m, 2H), 7.06 (t, *J* = 1.9 Hz, 1H), 6.88 (d, *J* = 1.9 Hz, 2H), 6.79–6.76 (m, 1H), 4.80 (t, *J* = 9.3 Hz, 1H), 3.90 (t, *J* = 9.2 Hz, 1H), 3.82 (t, *J* = 9.7 Hz, 1H), 2.50 (s, 3H); ^13^C NMR (100 MHz, CDCl_3_) δ 199.6, 195.6, 173.6, 152.3, 141.0, 140.7, 137.9, 137.3, 136.2, 134.7, 130.3, 128.8, 128.1, 127.2, 124.0, 123.5, 123.4, 123.0, 110.7, 80.0, 64.8, 55.8, 52.6, 35.8; IR (ATR): 3093, 2968, 2941, 2904, 2869, 2801, 1794, 1743, 1708, 1618, 1589, 1567, 1475, 1462, 1434, 1326, 1326, 1283, 1268, 1239, 1211, 1138, 1081, 1038, 1018, 983, 940, 862, 806, 773, 752, 690, 617, 595, 473, 454, 429 cm^−1^; HRMS (ESI): Exact mass calcd for C_26_H_17_Cl_2_NNaO_4_ [M+Na]^+^: 500.0432, Found: 500.0429.

Product **4x** was a yellow solid in 99% yield, Mp: 204.5–205.5 °C, mobile phase: PE/EA = 5:1; ^1^H NMR (400 MHz, CDCl_3_) δ 8.04 (dt, *J* = 7.7, 1.0 Hz, 1H), 7.90 (td, *J* = 7.5, 1.4 Hz, 2H), 7.78 (td, *J* = 7.5, 1.1 Hz, 1H), 7.72 (dt, *J* = 7.8, 1.1 Hz, 1H), 7.36 (t, *J* = 1.7 Hz, 1H), 7.30–7.21 (m, 1H), 7.25–7.22 (m, 1H), 7.06 (d, *J* = 1.7 Hz, 2H), 6.78–6.76 (m, 1H), 4.77 (t, *J* = 9.3 Hz, 1H), 3.90–3.79 (m, 2H), 2.49 (s, 3H). ^13^C NMR (100 MHz, CDCl_3_) δ 199.6, 195.5, 173.5, 152.4, 141.0, 140.7, 138.4, 137.2, 136.2, 133.6, 130.5, 130.3, 128.8, 123.9, 123.5, 123.4, 123.0, 122.7, 110.7, 79.9, 64.9, 56.0, 52.6, 35.7; IR (ATR): 3068, 2969, 2942, 2903, 2869, 2800, 1796, 1743, 1708, 1592, 1555, 1461, 1429, 1325, 1269, 1237, 1209, 1138, 1080, 1066, 1015, 981, 936, 859, 774, 750, 716, 689, 638, 616, 592, 470, 452, 416 cm^−1^; HRMS (ESI): Exact mass calcd for C_26_H_17_Br_2_NNaO_4_ [M+Na]+: 587.9416, Found: 587.9412.

### 3.4. Cell Culture

Human cancer cell lines K562 and Hela and mouse cancer cell line CT26 cells were cultured aseptically in RPMI-1640 media with 10% (*v*/*v*) FBS and 1% (*v*/*v*) penicillin–streptomycin at 37 °C with 5% CO_2_. All cells used in the experiment were in the logarithmic growth stage.

### 3.5. MTT Assay

A total of 5 × 10^3^ logarithmically growing cells were inoculated into each well of a 96-well plate and incubated for 24 h at 37 °C with 5% CO_2_. Subsequently, various concentrations of compounds were added to each well and incubated for an additional 48 h. Next, 10 μL of MTT reagent (5 mg/mL) was added to each well to react with the mitochondria of viable cells for approximately 4 h. Finally, the medium was discarded, and the blue crystals were completely dissolved in 150 μL of DMSO. Absorbance at a wavelength of 490 nm was measured using a microplate reader. The concentration of compounds that resulted in a 50% inhibition of cell growth (IC_50_) was calculated using IBM SPSS Statistics (version 19). The IC_50_ value for each compound was determined in a minimum of three independent experiments.

### 3.6. Docking Studies

Molecular docking studies were conducted using Schrödinger’s Maestro software to further elucidate the interactions between the synthesized compounds and antitumor tar-get proteins [30]. The detailed workflow is as follows:

Ligand Preparation: The synthesized compounds were prepared using the LigPrep module in Schrödinger Maestro software (version 12.8.117). The compounds were energy-minimized using the OPLS3 force field, and ionization states were generated under pH = 7.0 ± 2.0 conditions while retaining their stereochemistry to ensure the preparation of suitable ligand structures.

Protein Preparation: Crystal structures of eight antitumor target proteins were ob-tained from the Protein Data Bank (https://www.rcsb.org, accessed on 20 September 2024), including Bcl-xL (anti-apoptotic gene, PDB ID: 1YSI), VEGFR2 (vascular endothelial growth factor receptor 2, PDB ID: 1Y6B), TNFα (tumor necrosis factor alpha, PDB ID: 2AZ5), IL-2 (interleukin-2, PDB ID: 1M48), CDK-2 (cyclin-dependent kinase 2, PDB ID: 2BHE), Cox-2 (cyclooxygenase-2, PDB ID: 6COX), FTase (farnesyltransferase, PDB ID: 4GTM), and FAK (focal adhesion kinase, PDB ID: 4D4S). These protein structures were imported into Schrödinger Maestro and optimized using the Protein Preparation Wizard module. The active site was defined as the docking location, with the grid box size set to an inner box of 10 Å and an outer box of 20 Å to ensure the inclusion of the protein’s active pocket.

Molecular Docking: Protein–ligand docking was performed using the Ligand Docking module in Schrödinger Maestro. Initial docking was followed by re-docking with extra precision (XP), and the results were scored using the software’s built-in scoring function. A docking score with an absolute value greater than 5 was considered indicative of potential activity. The final docking results were visualized and analyzed in the Project Table module.

Binding Mode Analysis: After docking, the binding modes of the small molecules with the target proteins were visualized and analyzed using Discovery Studio Client software (version v19.1.0.18287). Detailed analysis of the binding modes revealed the interaction mechanisms between the compounds and the antitumor target proteins. This workflow systematically evaluates the activity of the compounds and elucidates their mechanisms of action, providing guidance for subsequent drug optimization and development.

## 4. Conclusions

In conclusion, we successfully synthesized 24 benzofuran spiro-pyrrolidine derivatives. Notably, under mild optimized conditions, this reaction has a broad substrate scope, high yield, and is easy to operate. It can quickly generate derivatives with potential biological activities. The desired products are obtained with a yield of 74–99% and a diastereomeric ratio of >20:1. ^1^H NMR, ^13^C NMR, and HRMS characterize the structures of all compounds. Activity tests found that compounds **4b** (IC_50_ = 15.14 ± 1.33 µM) and **4c** (IC_50_ = 10.26 ± 0.87 µM) have higher antiproliferative activities against human cervical cancer HeLa cells than cisplatin (IC_50_ = 15.91 ± 1.09 µM). Compounds **4e** (IC_50_ = 8.31 ± 0.64 µM) and **4s** (IC_50_ = 5.28 ± 0.72 µM) have better antiproliferative activities against mouse colon cancer CT26 cells than cisplatin (IC_50_ = 10.27 ± 0.71 µM). Molecular docking simulations found that compounds **4e** and **4s** may bind to some amino acid residues of antitumor target proteins through interactions such as hydrogen bonds and π-π conjugation, producing antitumor effects. This study will help find new bioactive molecules and provide a new strategy for discovering spiro-heterocyclic antitumor drugs based on targets.

## Figures and Tables

**Table 1:** Optimization of conditions ^a^.

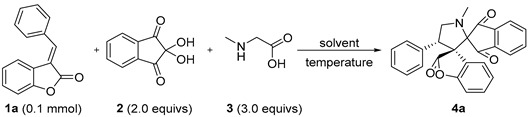					
**Entry**	**Solvent**	**t (°C)**	**Time (h)**	**Yield (%) ^b^**	**dr ^c^**
1	Acetonitrile	40	16	82	9:1
2	DCE	40	45	84	8:1
3	EtOAc	40	24	86	8:1
4	EtOH	40	16	88	6:1
5	Toluene	40	46	82	10:1
6 ^d^	H_2_O	40	24	trace	/
7	THF	40	21	90	15:1
8	THF	60	5	99	15:1
9	THF	80	5	88	15:1
10 ^e^	THF	60	5	84	15:1

^a^ The reaction was conducted using **1a** (0.1 mmol), **2** (0.2 mmol), and **3** (0.3 mmol) in solvent (1.0 mL). ^b^ Isolated yield. ^c^ Determined by ^1^H NMR of crude reaction mixture. ^d^ The reaction was carried out in the presence of 10 mol% additive tetra-nbutylammonium bromide (TBAB) at the specified temperature. ^e^ **1a** with 0.05 M.

**Table 2:** In vitro effect of compounds on the viability of cancer cells.

Compound	IC_50_ (μM) ^a^		Compound	IC_50_ (μM)	
	Hela	CT26		Hela	CT26
**4a**	65.55 ± 3.45	34.42 ± 1.82	**4n**	77.04 ± 3.16	89.72 ± 4.84
**4b**	15.14 ± 1.33	23.58 ± 1.34	**4o**	>100	>100
**4c**	10.26 ± 0.87	18.39 ±1.01	**4p**	55.18 ± 3.29	>100
**4d**	45.49 ± 2.42	21.96 ± 2.08	**4q**	37.15 ± 2.36	23.32 ± 1.36
**4e**	26.75 ± 1.53	8.31 ± 0.64	**4r**	24.18 ± 1.81	19.48 ± 1.35
**4f**	33.62 ± 1.68	25.67 ± 1.38	**4s**	17.48 ± 1.36	5.28 ± 0.72
**4g**	41.36 ± 2.48	36.42 ± 2.47	**4t**	46.53 ± 2.49	16.15 ± 1.06
**4h**	35.77 ± 2.34	58.66 ± 3.64	**4u**	30.46 ± 1.71	46.62 ± 3.63
**4i**	22.06 ± 1.22	49.17 ± 2.73	**4v**	>100	14.34 ± 0.65
**4j**	>100	26.54 ± 1.39	**4w**	71.39 ± 3.18	15.44 ± 1.12
**4k**	>100	53.41 ± 3.25	**4x**	>100	39.02± 2.02
**4l**	60.93 ± 3.48	>100	Cisplatin ^b^	15.91 ± 1.09	10.27 ± 0.71
**4m**	>100	>100			

^a^ IC_50_ concentration represents the concentration of a compound that produces a 50% reduction in cancer cell growth after 48 h of incubation. The given values are mean values of three experiments. ^b^ Cisplatin was used as a positive control.

**Table 3:** Molecular docking scoring results.

Compound	Docking Score							
	1YSI	1Y6B	2AZ5	1M48	2BHE	6COX	4GTM	4D4S
	(Bcl-x1)	(VEGFR2)	(TNFα)	(IL-2)	(CDK-2)	(Cox-2)	(FTase)	(FAK)
**4b**	−5.537	−3.168	−4.919	−1.833	−2.016	−2.782	−4.457	−2.067
**4c**	−6.842	−2.382	−7.053	−1.394	−1.053	−2.245	−5.760	−2.397
**4e**	−6.130	−6.596	−8.793	−3.203	−4.031	−4.806	−7.858	−2.711
**4s**	−5.807	−7.403	−8.331	−2.652	−3.263	−7.685	−8.226	−5.711

## Data Availability

Data are contained within the article and Supplementary Materials.

## Supplementary Materials

The following supporting information can be downloaded at: https://www.mdpi.com/article/10.3390/ijms252413580/s1.
