# Supplementary material for: Synthesis of Novel Benzofuran Spiro-2-Pyrrolidine Derivatives via [3+2] Azomethine Ylide Cycloadditions and Their Antitumor Activity
Source: Int J Mol Sci. 2024 Dec 19;25(24):13580. doi: 10.3390/ijms252413580 (PMC11676841; doi:10.3390/ijms252413580)
Supplement: Supplementary file 1 [file ijms-25-13580-s001.zip › ijms-3359594-supplementary.pdf]

Synthesis of Novel Benzofuran Spiro-2-Pyrrolidine Derivatives via [3+2] Azomethine Ylide Cycloadditions and Their Antitumor Activity

Bowen Pan [1,2,†], Tao Wang [1,†], Liangliang Zheng [1], Zhangchao Dong [1], Lijuan Liu [1], Xiongwei Liu [1], Tingting Feng [1], Ying Zhou [1,*] and Yang Shi [1,2,*]

[1] College of Pharmacy, Guizhou University of Traditional Chinese Medicine, Guiyang 550025, China; bwpan1105@163.com (B.P.); 18285244764@163.com (T.W.); zx15117508142@163.com (L.Z.); dongzhangchao0824@163.com (Z.D.); llj18385314855@163.com (L.L.); liuxiongwei058@gzy.edu.cn (X.L.); ftt0809@163.com (T.F.)

[2] State Key Laboratory of Natural and Biomimetic Drugs, School of Pharmaceutical Sciences, Peking University, Beijing 100191, China

* Correspondence: zhouying@gzy.edu.cn (Y.Z.); shiyang184@gzy.edu.cn (Y.S.)

† These authors contributed equally to this work.

Contents	Page
1. X-ray crystallographic data of compound **4s**	2–8
2. Cell Culture and MTT Assay	9
3. NMR spectra	10-61

1. X-ray crystallographic data of 4s

Data intensity of **4s**[1] was collected using a 'XtaLAB AFC12 (RINC)' diffractometer at 170.00(10) K. Data collection and reduction were done by using Olex2 and the structure was solved with the ShelXS structure solution program using Intrinsic Phasing and refined with the ShelXL refinement package using Least Squares minimization. Crystal data for **4s**: $C_{28}H_{23}NO_4$, T = 170.00(10) K, triclinic, P-1, a = 8.2152(8) Å, b = 11.7609(11) Å, c = 12.5248(12) Å, α = 74.799(8)°, β = 84.526(8)°, γ = 70.966(8)°, V = 1103.87(19) Å^3. Z = 2, ρ_{calc} = 1.316 g/cm^3. 7771 reflections collected, 3888 [R_{int} = 0.0293, R_{sigma} = 0.0492] independent reflections, R_1 = 0.0451, wR_2 = 0.0991 ($I > 2\sigma(I)$, final), R_1 = 0.0592, wR_2 = 0.1087 (all data), GOF = 1.052, and 301 parameters.

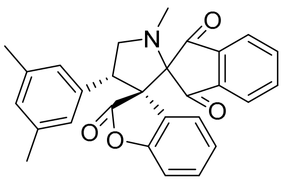

4s

≡

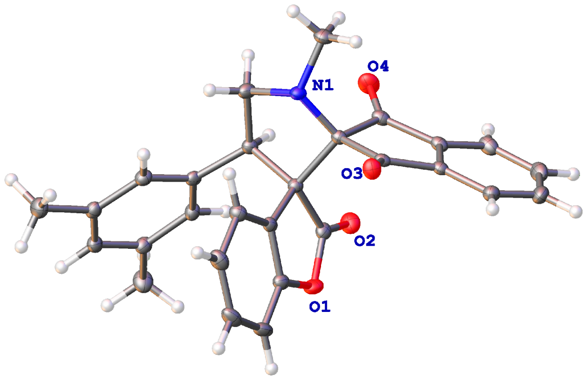

Table S1. Crystal data and structure refinement for 4s.

Identification code	4s
Empirical formula	$C_{28}H_{23}NO_4$
Formula weight	437.47
Temperature/K	170.00(10)
Crystal system	triclinic
Space group	P-1
a/Å	8.2152(8)
b/Å	11.7609(11)
c/Å	12.5248(12)
α/°	74.799(8)
β/°	84.526(8)
γ/°	70.966(8)
Volume/Å^3	1103.87(19)
Z	2
ρ_{calc}g/cm^3	1.316

[1] Supplementary crystallographic data have been deposited at Cambridge Crystallographic Data Center (CCDC number: 2403491).

μ/mm^{-1}	0.088
F(000)	460.0
Crystal size/mm^3	0.14 × 0.12 × 0.1
Radiation	Mo Kα (λ = 0.71073)
2Θ range for data collection/°	4.402 to 49.998
Index ranges	$-9 \leq h \leq 9$, $-13 \leq k \leq 12$, $-13 \leq l \leq 14$
Reflections collected	7771
Independent reflections	3888 [R_{int} = 0.0293, R_{sigma} = 0.0492]
Data/restraints/parameters	3888/0/301
Goodness-of-fit on F^2	1.052
Final R indexes [I>=2σ (I)]	R_1 = 0.0451, wR_2 = 0.0991
Final R indexes [all data]	R_1 = 0.0592, wR_2 = 0.1087
Largest diff. peak/hole / e Å^{-3}	0.28/-0.24

Table S2. Fractional Atomic Coordinates (×10^4) and Equivalent Isotropic Displacement Parameters (Å^2×10^3) for 4s. U_{eq} is defined as 1/3 of the trace of the orthogonalised U_{IJ} tensor.

Atom	*x*	*y*	*z*	U(eq)
O1	4382.5(15)	7823.0(12)	933.7(10)	36.0(3)
O2	2960.4(15)	6911.4(13)	2333.7(11)	41.3(4)
O3	5131.2(15)	10020.4(11)	3069.8(10)	35.2(3)
O4	3412.4(16)	6576.3(12)	4952.8(11)	43.5(4)
N1	6541.5(17)	7402.4(13)	4372.8(11)	27.2(3)
C1	7746(2)	5127.3(15)	2617.9(13)	25.9(4)
C2	9484(2)	5011.0(16)	2419.1(14)	29.9(4)
C3	10455(2)	4330.4(17)	1686.7(14)	30.8(4)
C4	9640(2)	3778.5(17)	1141.3(15)	33.0(4)
C5	7913(2)	3863.3(17)	1328.9(15)	32.6(4)
C6	6990(2)	4533.0(16)	2080.9(14)	29.4(4)
C7	6610(2)	5908.5(16)	3360.9(13)	27.2(4)
C8	7433(2)	6105.0(17)	4320.1(15)	34.8(4)
C9	5614.8(19)	7269.7(16)	2719.1(13)	24.4(4)
C10	4153(2)	7289.6(17)	2030.4(15)	29.4(4)
C11	5884(2)	8171.1(16)	835.1(14)	29.7(4)
C12	6650(2)	7900.9(15)	1841.1(13)	25.3(4)
C13	8150(2)	8188.7(17)	1869.9(15)	31.5(4)
C14	8814(2)	8752.5(19)	884.0(16)	41.2(5)
C15	7999(3)	9018.6(18)	-109.9(16)	43.6(5)
C16	6504(3)	8725.7(18)	-160.9(15)	39.3(5)
C17	4984.9(19)	7885.5(16)	3717.8(13)	24.6(4)
C18	4278(2)	9315.2(16)	3351.6(13)	24.2(4)

C19	2371(2)	9682.7(16)	3430.0(13)	26.0(4)
C20	1850(2)	8654.8(17)	3965.3(13)	28.0(4)
C21	3379(2)	7545.0(17)	4292.9(14)	29.0(4)
C22	112(2)	8763(2)	4144.9(15)	38.2(5)
C23	-1078(2)	9925(2)	3781.0(16)	43.5(5)
C24	-555(2)	10955(2)	3265.5(16)	43.1(5)
C25	1170(2)	10856.7(18)	3077.8(14)	34.9(5)
C26	6339(2)	7615(2)	5484.0(14)	39.4(5)
C27	7078(2)	3221(2)	750.3(18)	47.9(5)
C28	12346(2)	4181(2)	1497.5(17)	44.0(5)

Table S3. Anisotropic Displacement Parameters (Å^2×10^3) for 4s. The Anisotropic displacement factor exponent takes the form: -2π^2[h^{2}a*^{2}U$_{11}$+2hka*b*U$_{12}$+…].

Atom	U_{11}	U_{22}	U_{33}	U_{23}	U_{13}	U_{12}
O1	42.3(7)	38.5(8)	28.5(7)	-9.7(6)	-10.8(5)	-10.4(6)
O2	36.2(7)	47.2(9)	50.2(9)	-19.9(7)	-1.2(6)	-19.4(6)
O3	37.0(7)	26.5(7)	46.1(8)	-9.9(6)	4.6(6)	-16.1(6)
O4	55.3(8)	30.3(8)	42.6(8)	-0.9(7)	10.0(6)	-19.7(6)
N1	33.3(8)	28.0(9)	21.3(8)	-8.0(6)	-3.7(6)	-8.2(6)
C1	31.6(9)	18.4(9)	25.2(9)	-3.2(7)	-4.1(7)	-5.3(7)
C2	32.5(10)	27.5(11)	31.1(10)	-7.4(8)	-5.8(7)	-9.6(8)
C3	28.9(9)	27.8(11)	32.0(10)	-3.2(8)	-2.0(7)	-6.8(8)
C4	34.1(10)	29.2(11)	32.4(10)	-11.2(8)	-1.3(8)	-2.5(8)
C5	34.5(10)	27.6(11)	35.8(11)	-11.4(8)	-6.4(8)	-5.4(8)
C6	26.1(9)	25.8(10)	35.6(10)	-8.9(8)	-2.4(7)	-5.6(7)
C7	32.2(9)	22.5(10)	25.9(9)	-5.9(8)	-1.1(7)	-7.2(7)
C8	44.2(10)	28.1(11)	28.0(10)	-7.8(8)	-7.1(8)	-3.5(8)
C9	27.4(9)	23.8(10)	23.2(9)	-7.4(7)	-1.4(7)	-8.2(7)
C10	31.8(10)	24.9(10)	33.8(10)	-14.0(8)	-2.3(7)	-5.8(8)
C11	36.5(10)	24.2(10)	27.2(10)	-8.7(8)	-2.4(7)	-5.2(8)
C12	29.9(9)	20.1(9)	24.2(9)	-7.3(7)	1.2(7)	-4.3(7)
C13	36.1(10)	30.2(11)	30.5(10)	-9.1(8)	0.9(8)	-12.5(8)
C14	46.1(11)	38.7(12)	42.4(12)	-13.7(10)	13.7(9)	-19.2(9)
C15	61.5(13)	33.4(12)	32.0(11)	-7.4(9)	15.3(9)	-14.6(10)
C16	59.0(13)	30.3(11)	20.8(10)	-5.2(8)	-1.0(8)	-4.3(9)
C17	27.6(9)	26.1(10)	22.0(9)	-7.3(7)	0.6(7)	-10.2(7)
C18	31.7(9)	25.6(10)	18.3(9)	-8.6(7)	1.0(7)	-11.0(8)
C19	30.6(9)	29.7(11)	20.5(9)	-10.7(8)	1.2(7)	-10.2(8)
C20	32.7(9)	33.8(11)	24.3(9)	-13.4(8)	3.6(7)	-15.4(8)
C21	39.6(10)	29.1(11)	24.4(10)	-10.8(8)	3.6(7)	-16.4(8)

C22	37.0(11)	52.1(14)	36.8(11)	-20.7(10)	6.7(8)	-23.4(10)
C23	27.6(10)	65.7(16)	42.9(12)	-24.2(11)	-0.7(8)	-13.5(10)
C24	34.7(11)	49.0(14)	37.6(11)	-12.6(10)	-7.7(8)	1.1(9)
C25	36.4(10)	36.5(12)	28.4(10)	-8.8(8)	-2.0(8)	-5.7(8)
C26	47.7(11)	45.6(13)	26.5(10)	-15.2(9)	-2.9(8)	-11.1(9)
C27	44.5(11)	50.8(14)	58.6(14)	-29.9(11)	-3.0(10)	-14.8(10)
C28	33.8(11)	49.5(14)	47.7(12)	-13.2(10)	1.7(9)	-11.0(9)

Table S4. Bond Lengths for 4s.

Atom	Atom	Length/Å	Atom	Atom	Length/Å
O1	C10	1.375(2)	C9	C10	1.535(2)
O1	C11	1.407(2)	C9	C12	1.513(2)
O2	C10	1.195(2)	C9	C17	1.570(2)
O3	C18	1.218(2)	C11	C12	1.377(2)
O4	C21	1.213(2)	C11	C16	1.382(2)
N1	C8	1.478(2)	C12	C13	1.386(2)
N1	C17	1.454(2)	C13	C14	1.393(2)
N1	C26	1.462(2)	C14	C15	1.384(3)
C1	C2	1.394(2)	C15	C16	1.391(3)
C1	C6	1.391(2)	C17	C18	1.544(2)
C1	C7	1.518(2)	C17	C21	1.559(2)
C2	C3	1.395(2)	C18	C19	1.483(2)
C3	C4	1.392(3)	C19	C20	1.397(2)
C3	C28	1.508(2)	C19	C25	1.396(2)
C4	C5	1.391(2)	C20	C21	1.482(2)
C5	C6	1.395(2)	C20	C22	1.393(2)
C5	C27	1.512(3)	C22	C23	1.385(3)
C7	C8	1.535(2)	C23	C24	1.394(3)
C7	C9	1.573(2)	C24	C25	1.386(3)

Table S5. Bond Angles for 4s.

Atom	Atom	Atom	Angle/°	Atom	Atom	Atom	Angle/°
C10	O1	C11	108.21(13)	C16	C11	O1	123.45(16)
C17	N1	C8	109.25(13)	C11	C12	C9	108.15(14)
C17	N1	C26	115.81(13)	C11	C12	C13	118.63(15)
C26	N1	C8	115.06(14)	C13	C12	C9	133.17(15)
C2	C1	C7	123.40(15)	C12	C13	C14	118.87(17)
C6	C1	C2	118.60(15)	C15	C14	C13	120.78(18)
C6	C1	C7	117.95(14)	C14	C15	C16	121.37(17)

C1	C2	C3	121.25(16)	C11	C16	C15	116.05(18)
C2	C3	C28	120.97(16)	N1	C17	C9	100.93(12)
C4	C3	C2	118.42(15)	N1	C17	C18	114.67(14)
C4	C3	C28	120.60(16)	N1	C17	C21	116.18(14)
C5	C4	C3	121.87(16)	C18	C17	C9	112.85(13)
C4	C5	C6	118.15(16)	C18	C17	C21	102.02(12)
C4	C5	C27	120.65(16)	C21	C17	C9	110.62(13)
C6	C5	C27	121.19(16)	O3	C18	C17	126.18(15)
C1	C6	C5	121.66(15)	O3	C18	C19	125.87(16)
C1	C7	C8	119.01(14)	C19	C18	C17	107.93(14)
C1	C7	C9	113.43(13)	C20	C19	C18	110.08(15)
C8	C7	C9	103.09(14)	C25	C19	C18	128.77(16)
N1	C8	C7	105.93(13)	C25	C19	C20	121.13(16)
C10	C9	C7	111.16(14)	C19	C20	C21	109.95(14)
C10	C9	C17	114.14(13)	C22	C20	C19	121.04(17)
C12	C9	C7	115.54(13)	C22	C20	C21	128.99(17)
C12	C9	C10	101.38(13)	O4	C21	C17	125.47(16)
C12	C9	C17	115.22(14)	O4	C21	C20	126.79(15)
C17	C9	C7	99.99(12)	C20	C21	C17	107.52(14)
O1	C10	C9	109.97(14)	C23	C22	C20	117.70(19)
O2	C10	O1	121.36(16)	C22	C23	C24	121.21(17)
O2	C10	C9	128.66(16)	C25	C24	C23	121.56(18)
C12	C11	O1	112.25(14)	C24	C25	C19	117.34(19)
C12	C11	C16	124.30(17)				

Table S6. Torsion Angles for 4s.

A	B	C	D	Angle/°	A	B	C	D	Angle/°
O1	C11	C12	C9	1.86(19)	C10	C9	C17	N1	163.55(14)
O1	C11	C12	C13	179.72(14)	C10	C9	C17	C18	-73.59(17)
O1	C11	C16	C15	179.53(16)	C10	C9	C17	C21	39.99(19)
O3	C18	C19	C20	-169.35(16)	C11	O1	C10	O2	-179.91(16)
O3	C18	C19	C25	8.9(3)	C11	O1	C10	C9	-0.39(18)
N1	C17	C18	O3	37.4(2)	C11	C12	C13	C14	0.7(2)
N1	C17	C18	C19	-141.17(13)	C12	C9	C10	O1	1.40(17)
N1	C17	C21	O4	-34.2(2)	C12	C9	C10	O2	-179.14(18)
N1	C17	C21	C20	140.71(15)	C12	C9	C17	N1	-79.70(16)
C1	C2	C3	C4	0.9(3)	C12	C9	C17	C18	43.16(18)
C1	C2	C3	C28	-178.42(16)	C12	C9	C17	C21	156.74(14)
C1	C7	C8	N1	141.77(15)	C12	C11	C16	C15	-0.1(3)
C1	C7	C9	C10	72.66(17)	C12	C13	C14	C15	-0.2(3)

C1	C7	C9	C12	-42.1(2)	C13	C14	C15	C16	-0.5(3)
C1	C7	C9	C17	-166.45(13)	C14	C15	C16	C11	0.7(3)
C2	C1	C6	C5	-2.1(3)	C16	C11	C12	C9	-178.43(16)
C2	C1	C7	C8	-28.4(2)	C16	C11	C12	C13	-0.6(3)
C2	C1	C7	C9	93.07(19)	C17	N1	C8	C7	14.35(18)
C2	C3	C4	C5	-1.6(3)	C17	C9	C10	O1	125.91(15)
C3	C4	C5	C6	0.5(3)	C17	C9	C10	O2	-54.6(2)
C3	C4	C5	C27	-178.12(18)	C17	C9	C12	C11	-125.68(15)
C4	C5	C6	C1	1.3(3)	C17	C9	C12	C13	56.9(2)
C6	C1	C2	C3	0.9(3)	C17	C18	C19	C20	9.26(17)
C6	C1	C7	C8	153.80(16)	C17	C18	C19	C25	-172.51(16)
C6	C1	C7	C9	-84.69(19)	C18	C17	C21	O4	-159.63(17)
C7	C1	C2	C3	-176.82(16)	C18	C17	C21	C20	15.25(16)
C7	C1	C6	C5	175.81(16)	C18	C19	C20	C21	0.96(18)
C7	C9	C10	O1	-121.93(14)	C18	C19	C20	C22	179.72(15)
C7	C9	C10	O2	57.5(2)	C18	C19	C25	C24	-179.03(16)
C7	C9	C12	C11	118.37(15)	C19	C20	C21	O4	164.11(17)
C7	C9	C12	C13	-59.0(2)	C19	C20	C21	C17	-10.68(18)
C7	C9	C17	N1	44.82(14)	C19	C20	C22	C23	-0.4(3)
C7	C9	C17	C18	167.69(13)	C20	C19	C25	C24	-1.0(3)
C7	C9	C17	C21	-78.73(15)	C20	C22	C23	C24	-0.8(3)
C8	N1	C17	C9	-37.61(16)	C21	C17	C18	O3	163.88(16)
C8	N1	C17	C18	-159.20(13)	C21	C17	C18	C19	-14.72(16)
C8	N1	C17	C21	82.04(17)	C21	C20	C22	C23	178.08(17)
C8	C7	C9	C10	-157.28(13)	C22	C20	C21	O4	-14.5(3)
C8	C7	C9	C12	87.92(16)	C22	C20	C21	C17	170.69(16)
C8	C7	C9	C17	-36.39(15)	C22	C23	C24	C25	1.2(3)
C9	C7	C8	N1	15.19(17)	C23	C24	C25	C19	-0.3(3)
C9	C12	C13	C14	177.96(17)	C25	C19	C20	C21	-177.43(15)
C9	C17	C18	O3	-77.4(2)	C25	C19	C20	C22	1.3(2)
C9	C17	C18	C19	104.00(14)	C26	N1	C8	C7	146.60(14)
C9	C17	C21	O4	80.1(2)	C26	N1	C17	C9	-169.46(14)
C9	C17	C21	C20	-105.04(15)	C26	N1	C17	C18	68.94(19)
C10	O1	C11	C12	-0.93(19)	C26	N1	C17	C21	-49.8(2)
C10	O1	C11	C16	179.35(16)	C27	C5	C6	C1	179.99(18)
C10	C9	C12	C11	-1.90(17)	C28	C3	C4	C5	177.67(17)
C10	C9	C12	C13	-179.33(18)					

Table S7. Hydrogen Atom Coordinates (Å×10[4]) and Isotropic Displacement Parameters (Å[2]×10[3]) for 4s.

Atom	*x*	*y*	*z*	U(eq)
H2	10018.91	5402.46	2789.39	36
H4	10282.05	3331.35	626.52	40
H6	5815.74	4584.54	2230.03	35
H7	5720.16	5503.26	3693.84	33
H8A	7277.29	5523.49	5023.6	42
H8B	8681.06	5968.02	4180.92	42
H13	8715.97	8004.49	2550.47	38
H14	9839.44	8956.79	894.11	49
H15	8470.14	9409.47	-771.24	52
H16	5941.42	8896.74	-840.3	47
H22	-244.41	8064	4504.38	46
H23	-2271.99	10022.78	3884.68	52
H24	-1401.94	11742.98	3037.52	52
H25	1520.99	11559.76	2723.59	42
H26A	5804.31	8502.85	5436.02	59
H26B	7471.2	7329.47	5828.09	59
H26C	5606.03	7154.21	5932.71	59
H27A	6789.41	2545.11	1294.06	72
H27B	7878.24	2882.55	195.54	72
H27C	6026.02	3819.13	386.17	72
H28A	13025.62	3383.48	1958.14	66
H28B	12604.17	4856.54	1695.29	66
H28C	12637.7	4206.11	716.32	66

2. Cell Culture and MTT Assay

Human cancer cell lines (K562 and HeLa) and the mouse cancer cell line CT26 were cultured in an incubator at 37°C with 5% CO_2. The culture medium used was RPMI-1640 supplemented with 10% (v/v) fetal bovine serum (FBS) and 1% (v/v) penicillin-streptomycin solution. All cells used in the experiment were in the logarithmic growth phase.

For the MTT assay, 5×10^3 logarithmic-phase cells were seeded into each well of a 96-well plate. After 24 hours of incubation at 37°C and 5% CO_2, different concentrations of the compounds (100, 50, 25, 12.5, 6.25 μM) were added to each well (five replicates for each concentration) and further incubated for 48 hours. Then, 10 μL of freshly prepared MTT solution (5 mg/mL, stored in the dark) was added to each well and allowed to react with the mitochondria of viable cells for about 4 hours. Subsequently, the culture medium in the 96-well plate was removed, and 150 μL of DMSO was added to dissolve the blue formazan crystals (with shaking for approximately 10 minutes) until completely dissolved. The absorbance was measured at 490 nm using a microplate reader.

The concentration of the compound that inhibited 50% of cell growth (IC_{50}) was calculated and analyzed using IBM SPSS Statistics (version 19). The IC_{50} values for each compound were determined based on at least three independent experiments.

Cell seeding density and incubation time: Different cell lines have varying attachment areas. A preliminary experiment is required to determine the optimal cell seeding density and incubation time to ensure a linear relationship between the amount of MTT crystals formed and the number of cells.

Preparation and storage of MTT: MTT is best prepared freshly and stored in the dark at 4°C for up to two weeks after filtration, or prepared as a 5 mg/mL stock solution and stored at -20°C for long-term use. Avoid repeated freeze-thaw cycles.

Carcinogenicity of MTT: MTT is carcinogenic. Handle with care and wear transparent film gloves and a mask during use.

Avoiding serum interference: High serum concentrations may affect the absorbance of experimental wells. Therefore, use a culture medium with less than 10% FBS for the experiment.

Contamination detection: If individual wells turn blue-black immediately after adding MTT, contamination is likely. Microscopic observation before adding MTT can help detect contamination.

Steps before adding DMSO: Carefully remove the liquid from the wells before adding DMSO to avoid interfering with the experimental results. When adding DMSO, avoid creating bubbles, which may affect the results.

Experimental environment: Ensure the experiment is conducted in a sterile, dust-free, temperature-controlled (37°C), and humidity-controlled environment to maintain optimal cell growth conditions.

Dissolving formazan crystals: Ensure thorough shaking when dissolving formazan crystals to achieve complete dissolution.

Edge effects: Be cautious of edge effects, as the faster evaporation of liquid in edge wells may lead to higher drug concentrations and more complex cell conditions. Typically, only the central 60 wells are used for the experiment, and the outer 36 wells are filled with 100 μL of sterile PBS.

Drug concentration: To fit the IC_{50}, set five concentrations of the compound with five replicates for each. Ensure that the highest concentration suppresses at least 90% of cell growth.

Light protection: MTT should be stored in the dark at 4°C for up to two weeks or prepared as a 5 mg/mL stock solution and stored at -20°C for long-term use. Avoid repeated freeze-thaw cycles.

3. NMR spectra

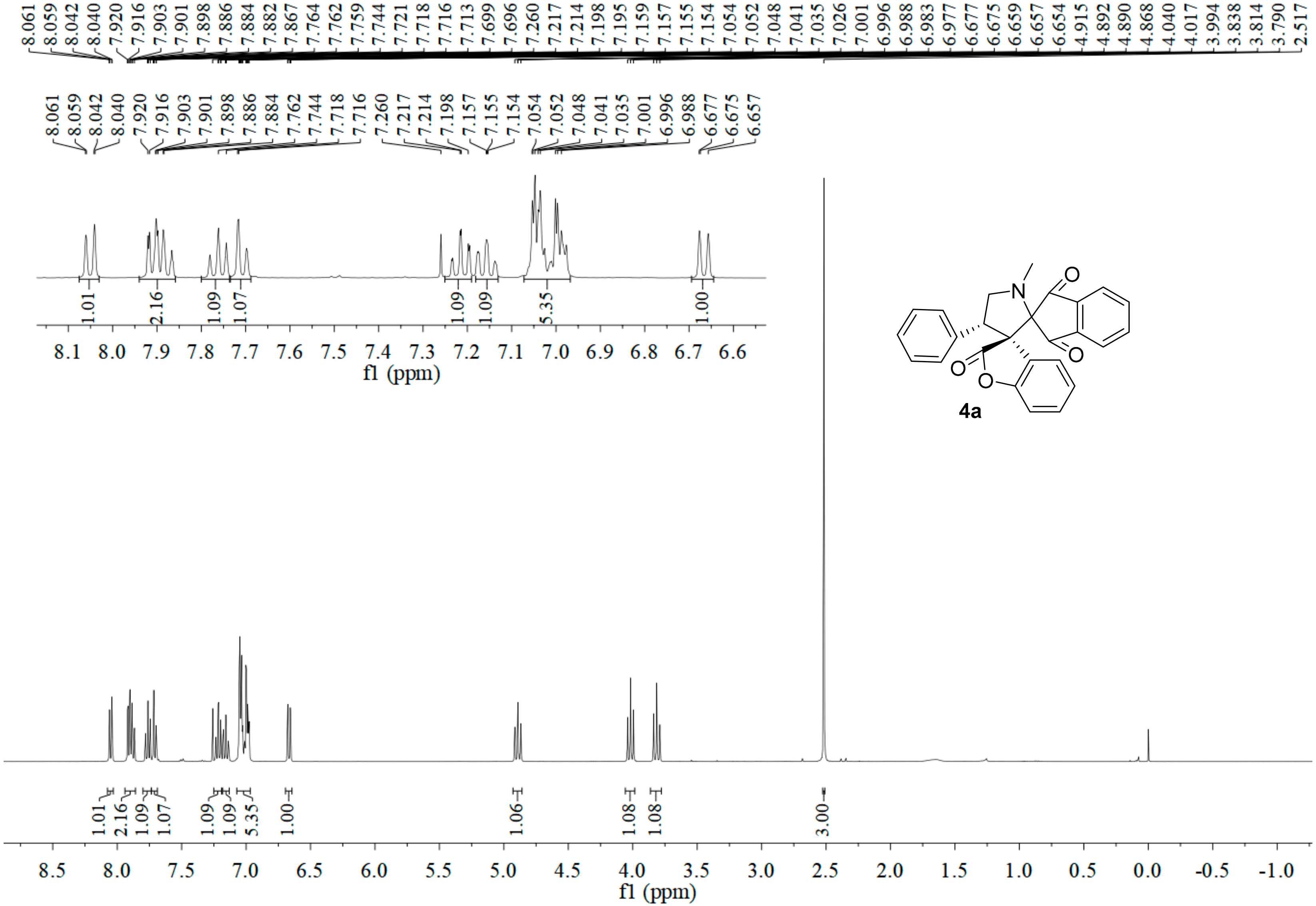

^{1}H NMR of Compound **4a** (400 MHz, $CDCl_3$)

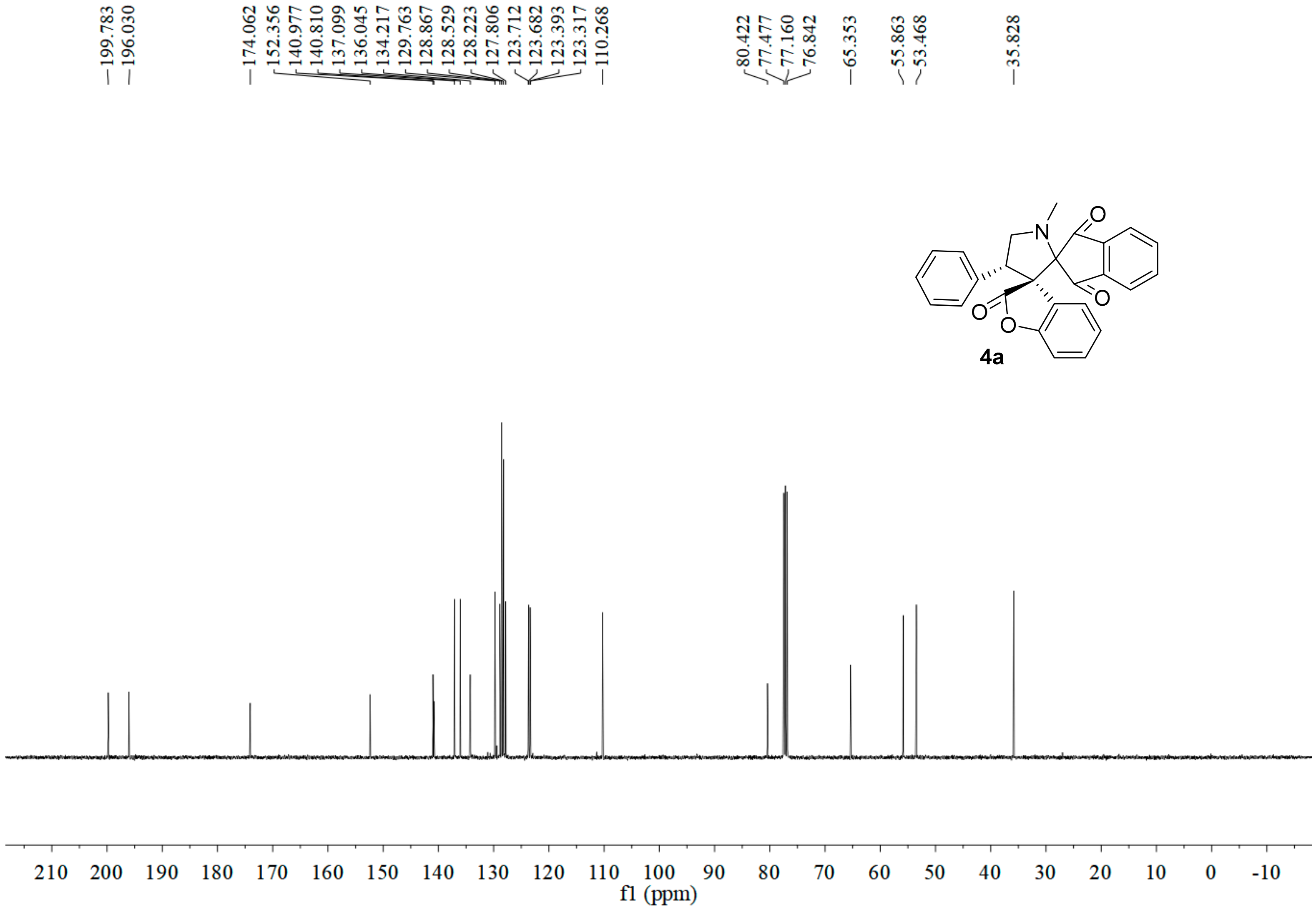

^{13}C NMR of Compound **4a** (100 MHz, $CDCl_3$)

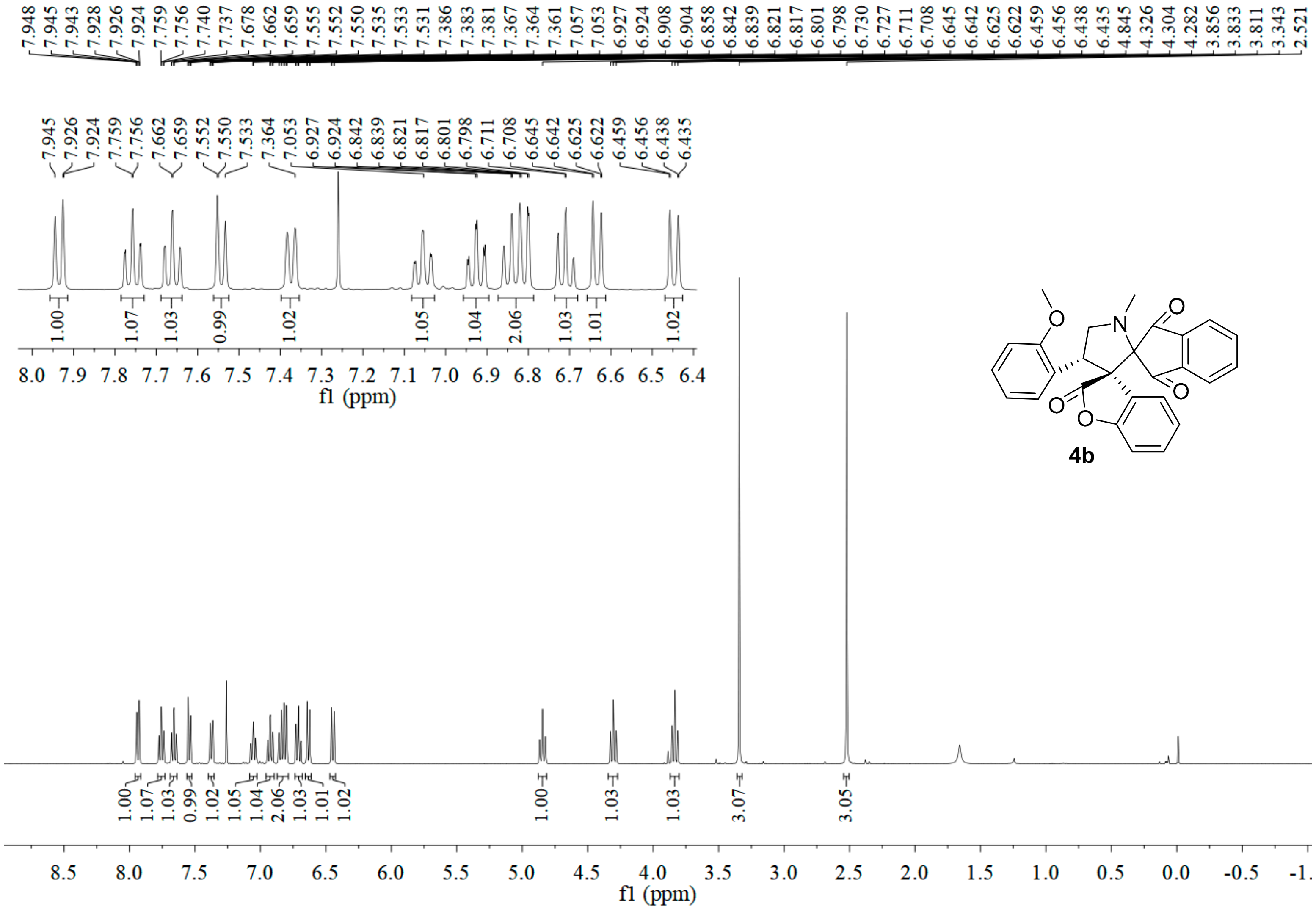

^{1}H NMR of Compound **4b** (400 MHz, $CDCl_3$)

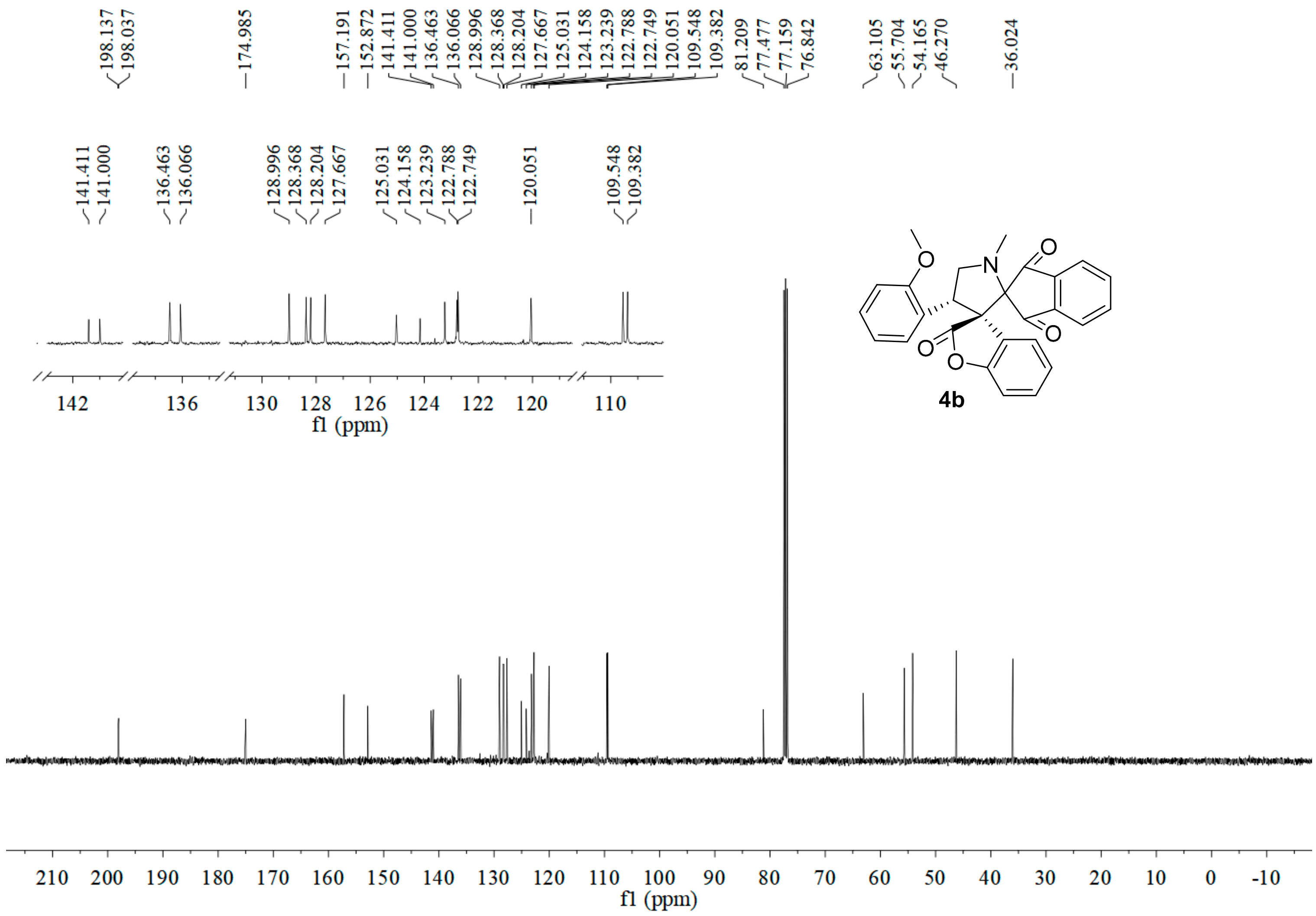

^{13}C NMR of Compound **4b** (100 MHz, $CDCl_3$)

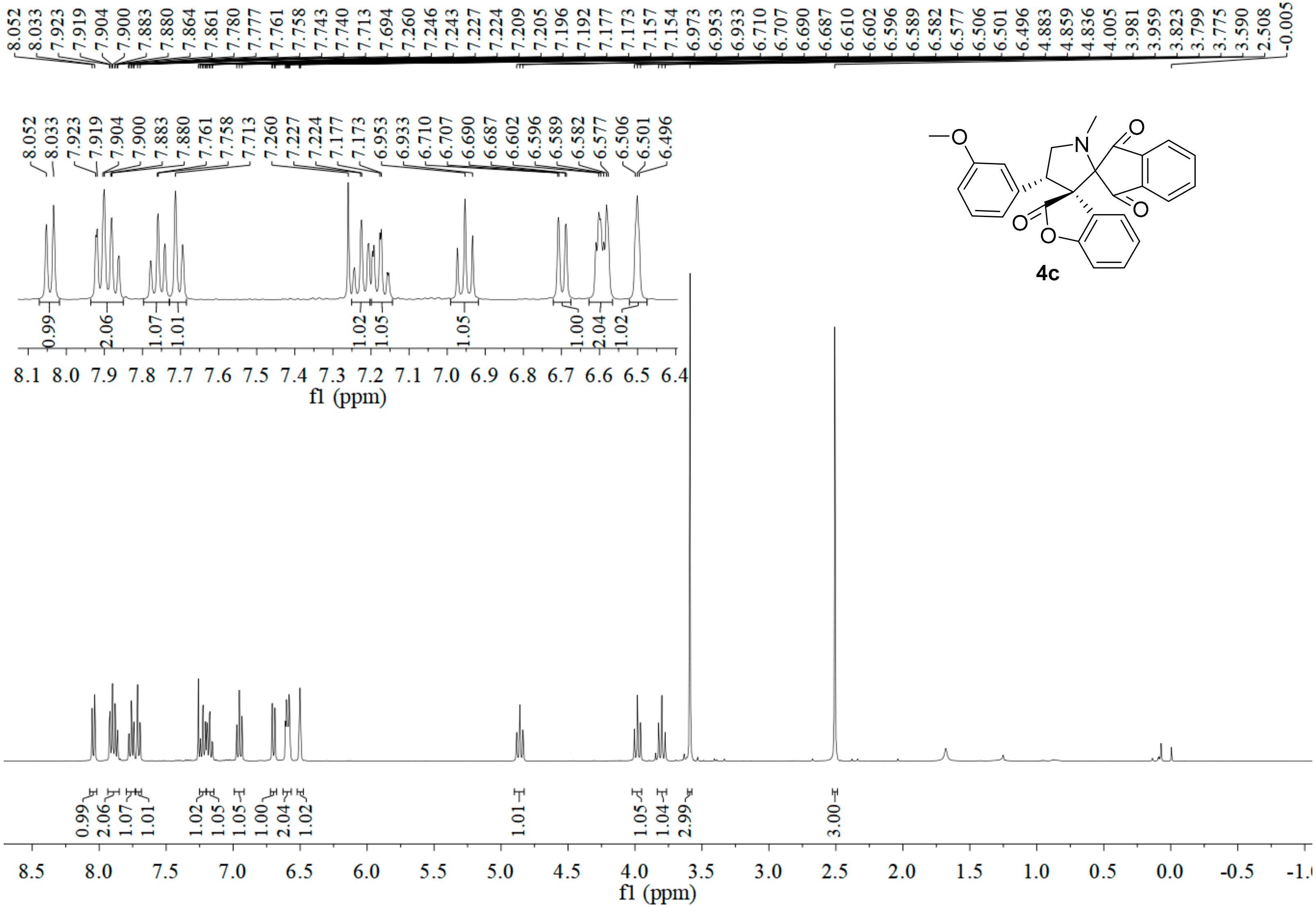

^{1}H NMR of Compound **4c** (400 MHz, $CDCl_3$)

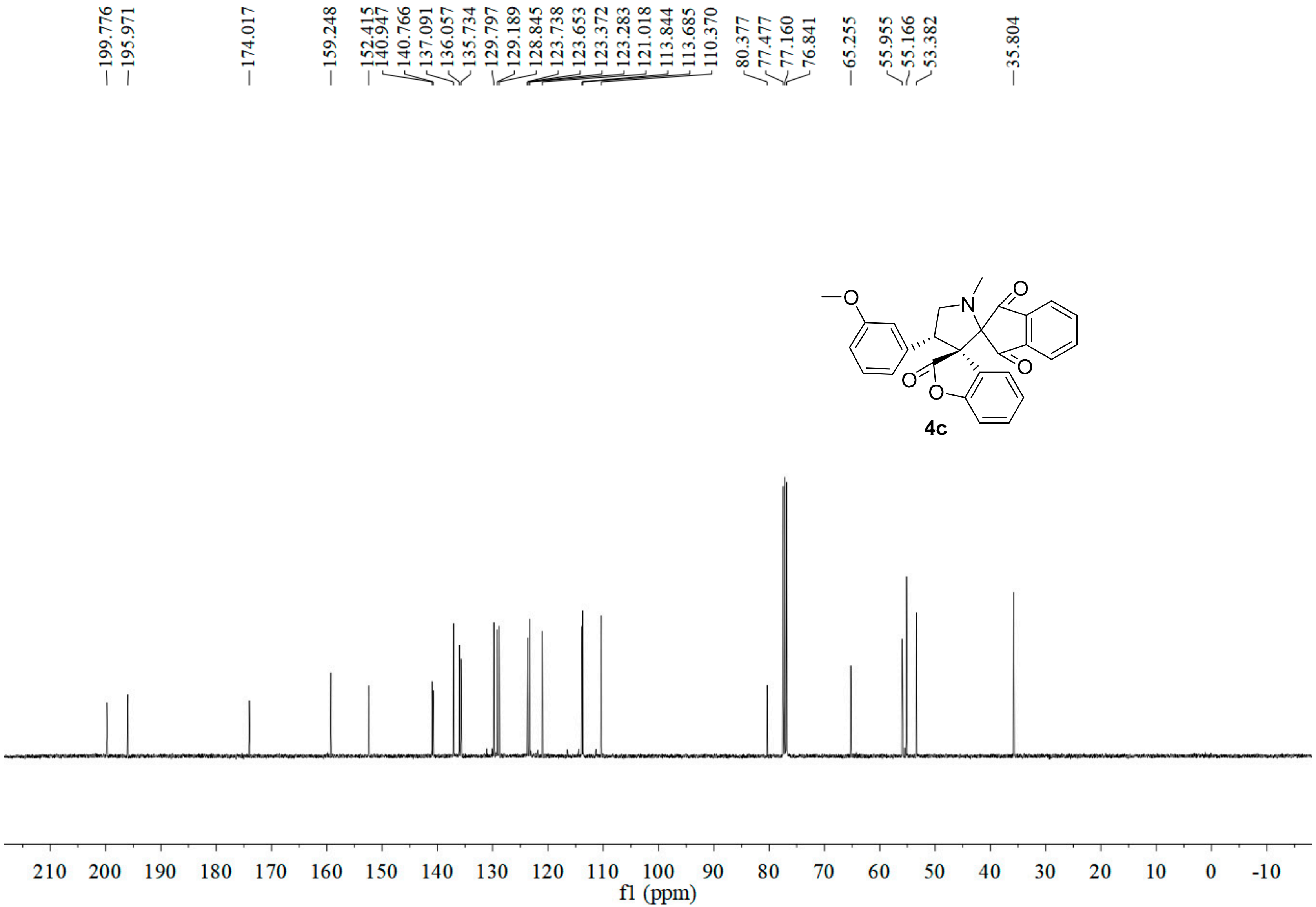

^{13}C NMR of Compound **4c** (100 MHz, $CDCl_3$)

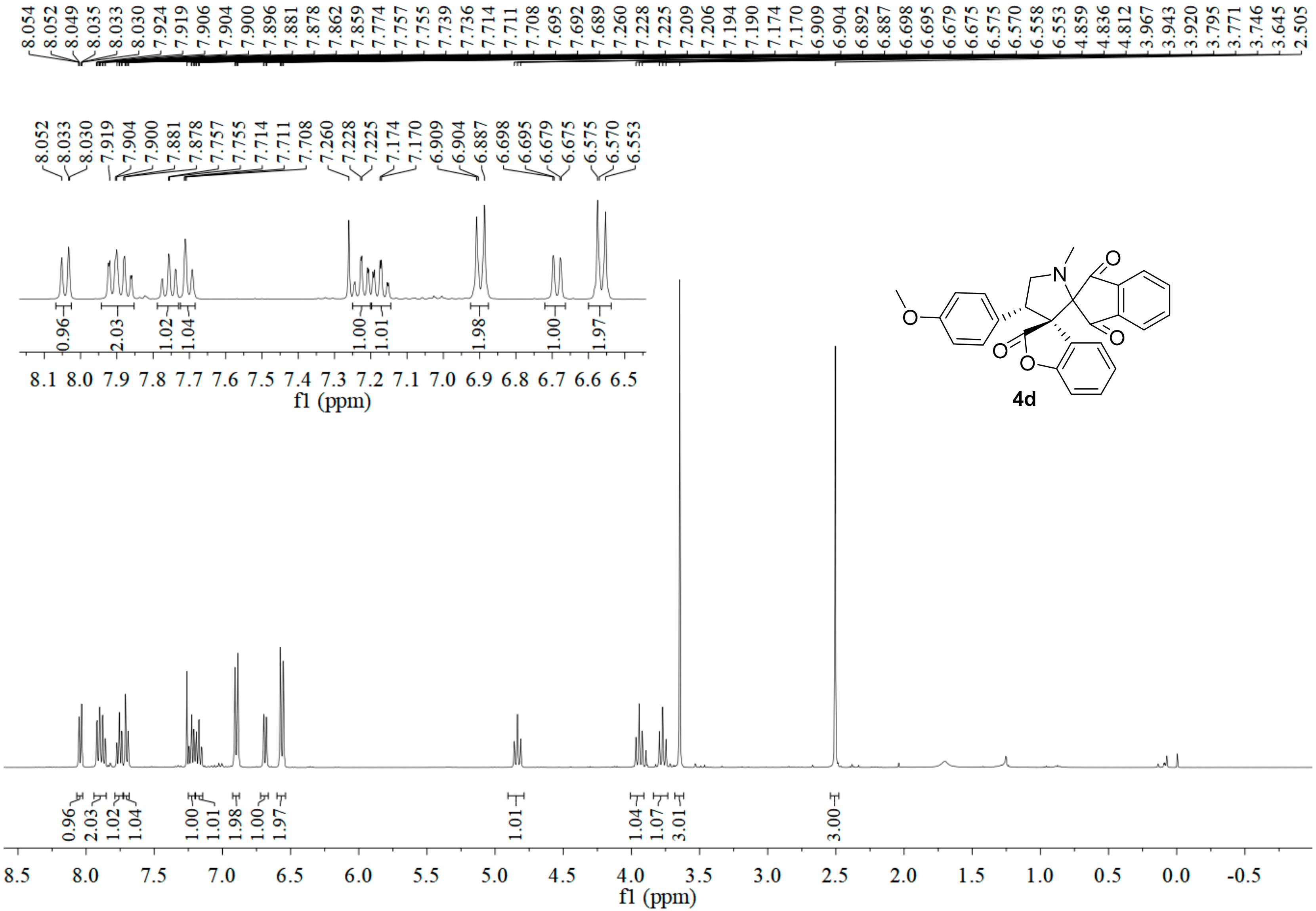

^{1}H NMR of Compound **4d** (400 MHz, $CDCl_3$)

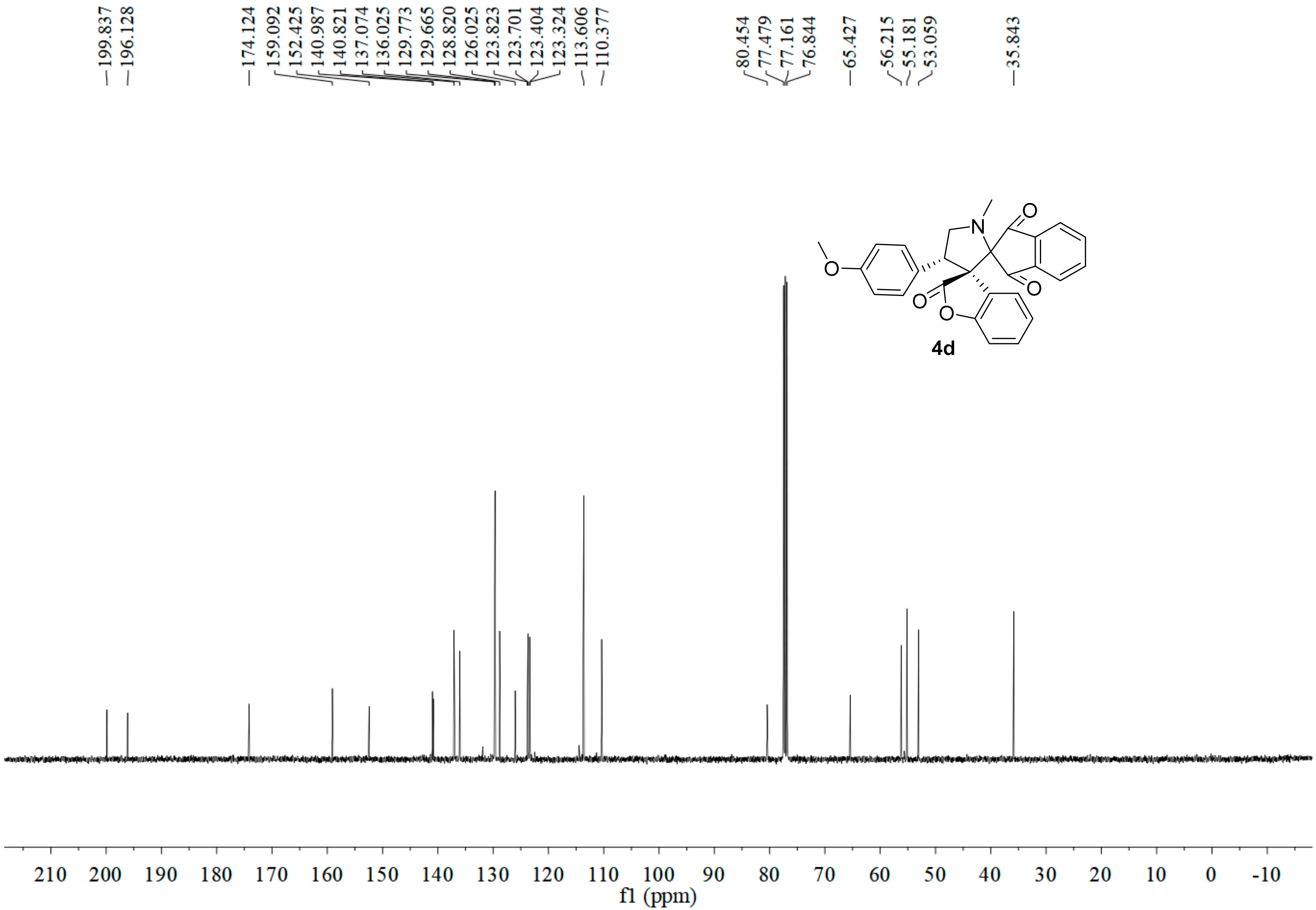

^{13}C NMR of Compound **4d** (100 MHz, $CDCl_3$)

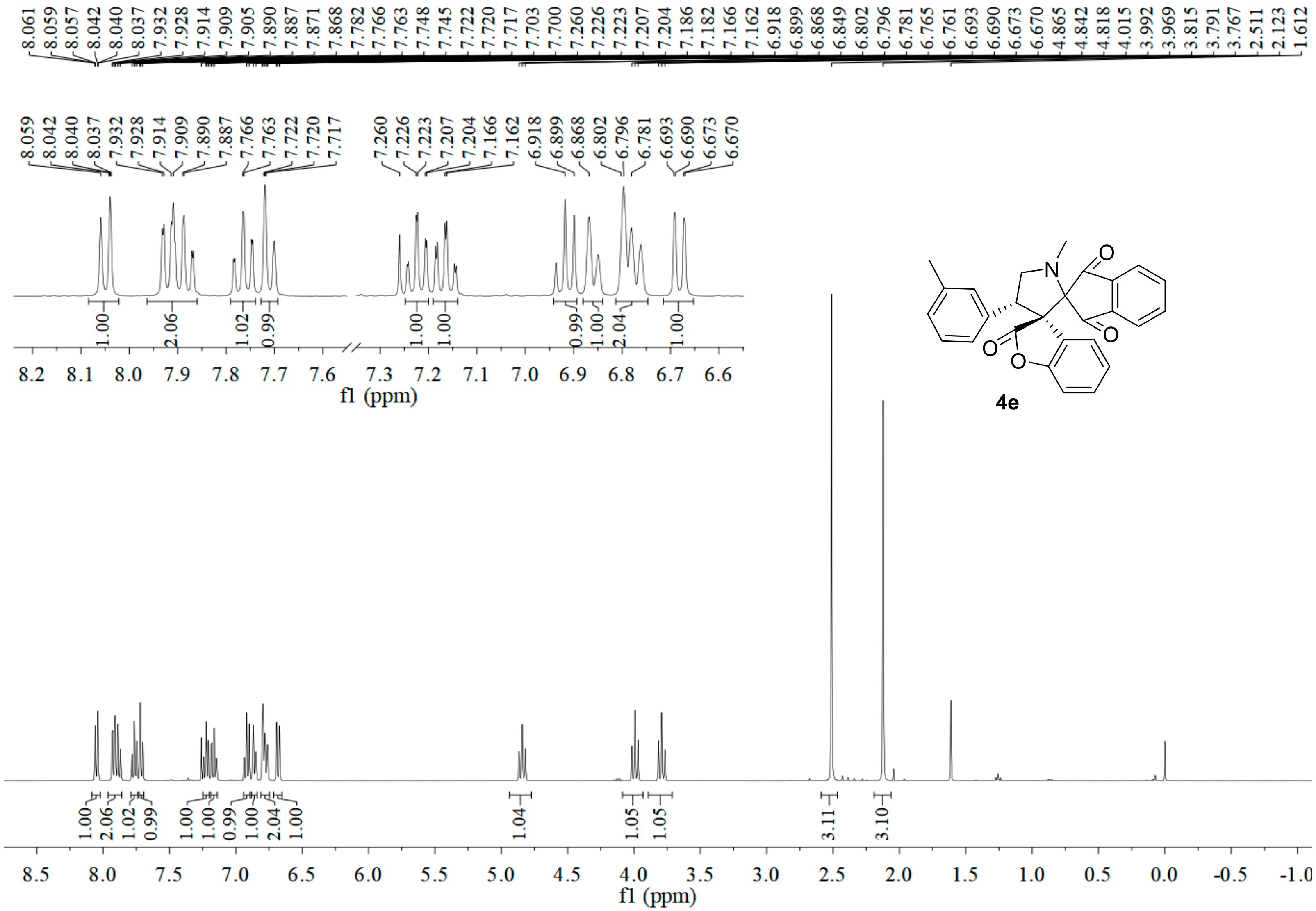

^{1}H NMR of Compound **4e** (400 MHz, $CDCl_3$)

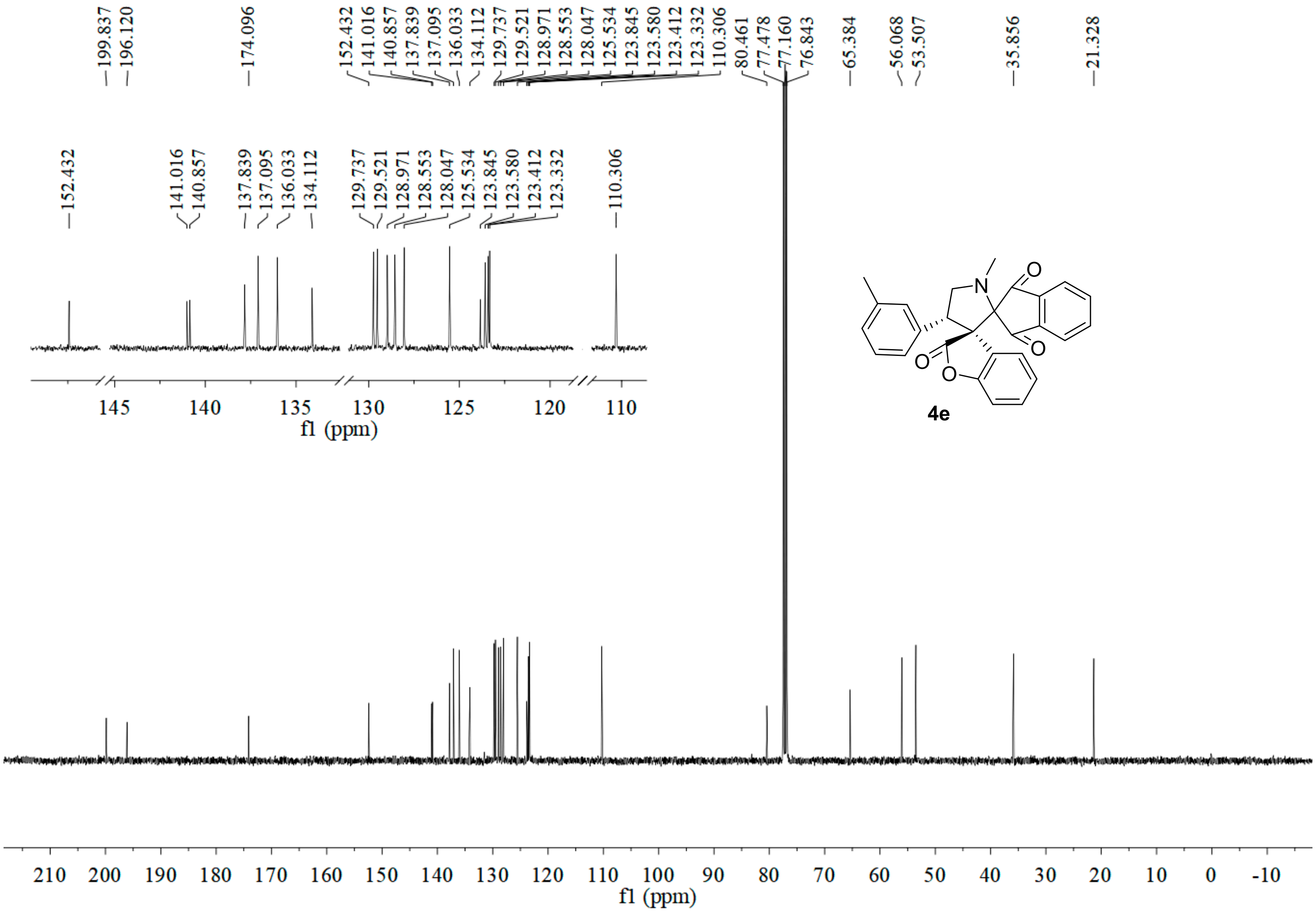

^{13}C NMR of Compound **4e** (100 MHz, $CDCl_3$)

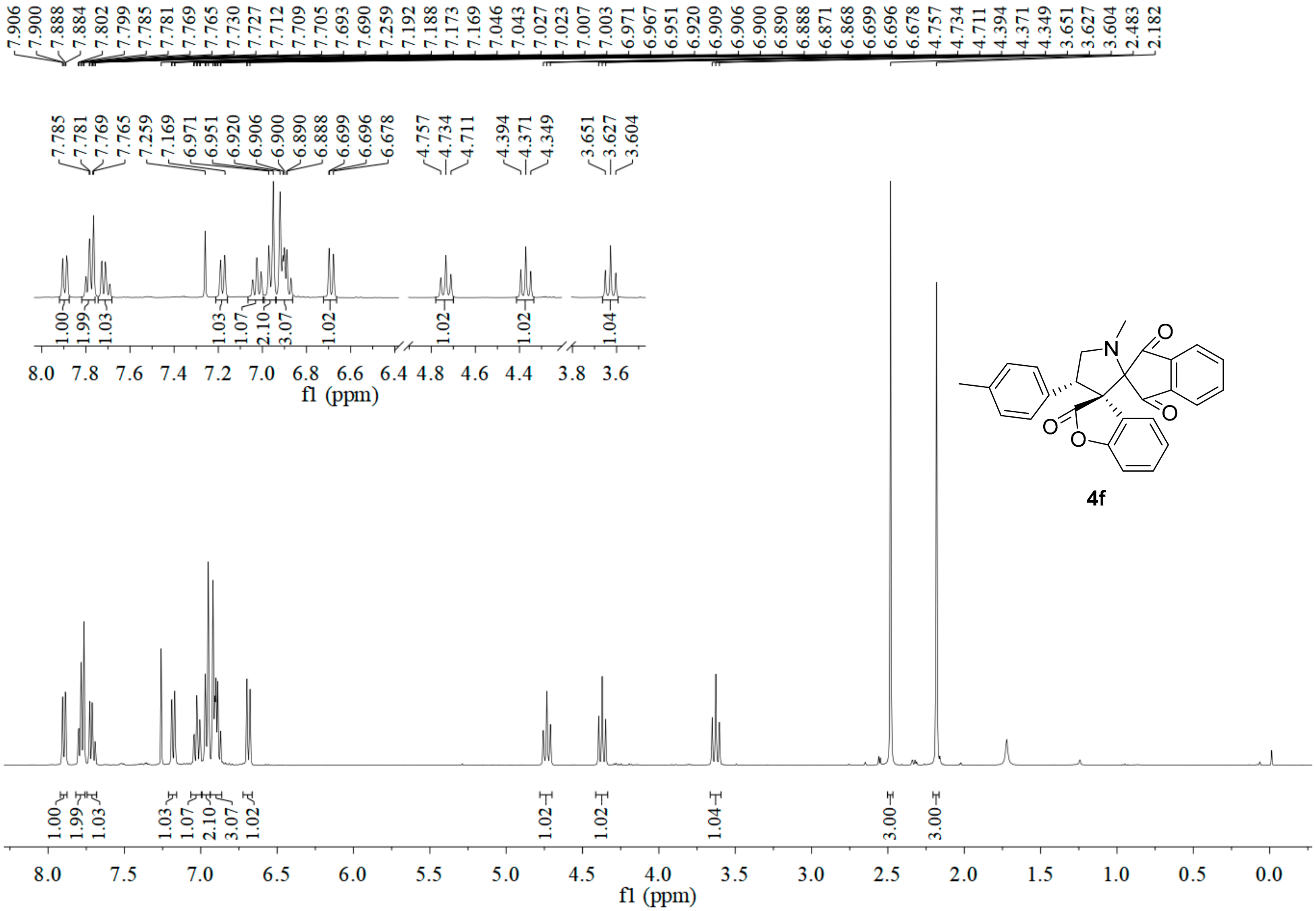

^{1}H NMR of Compound **4f** (400 MHz, $CDCl_3$)

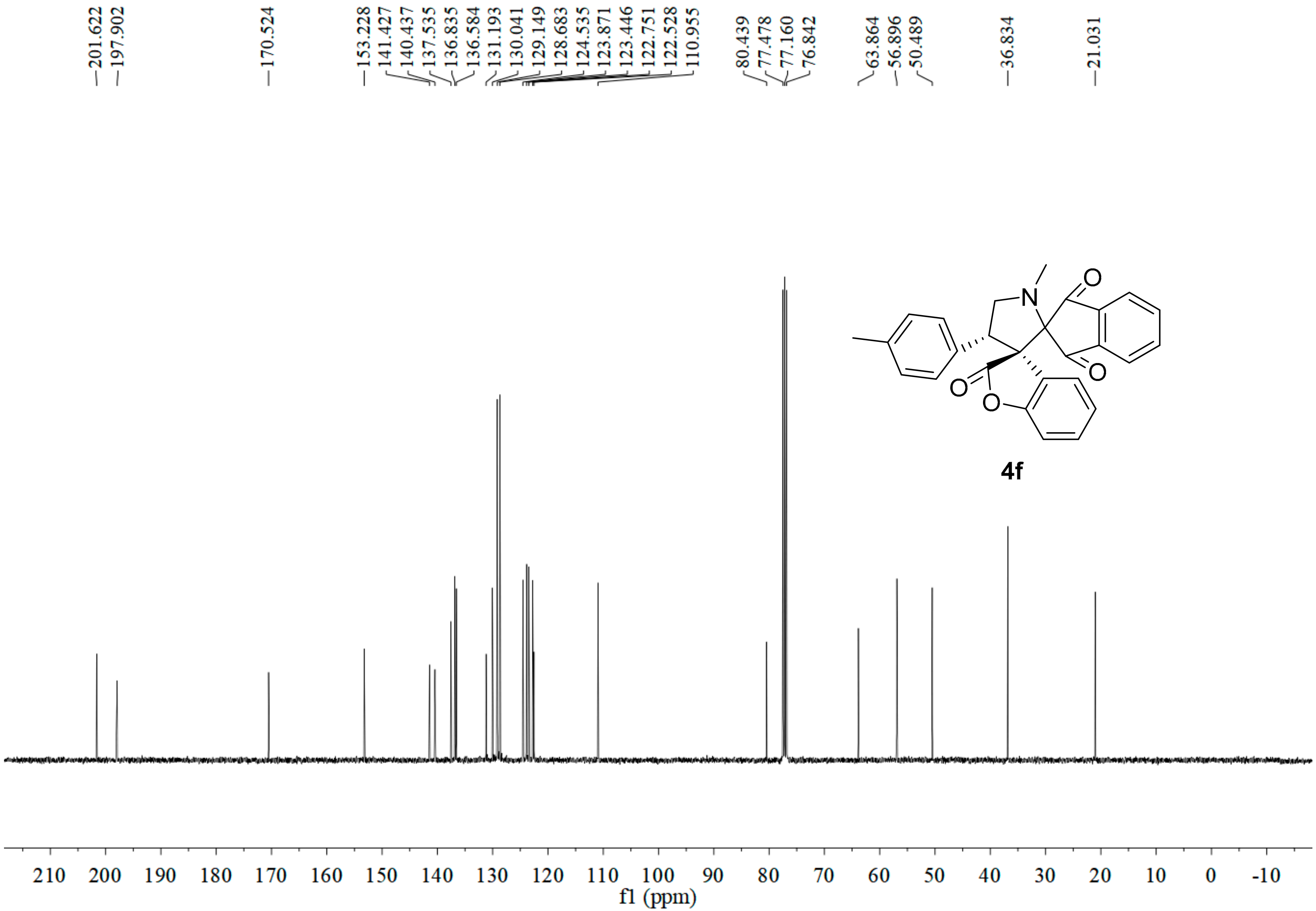

^{13}C NMR of Compound **4f** (100 MHz, $CDCl_3$)

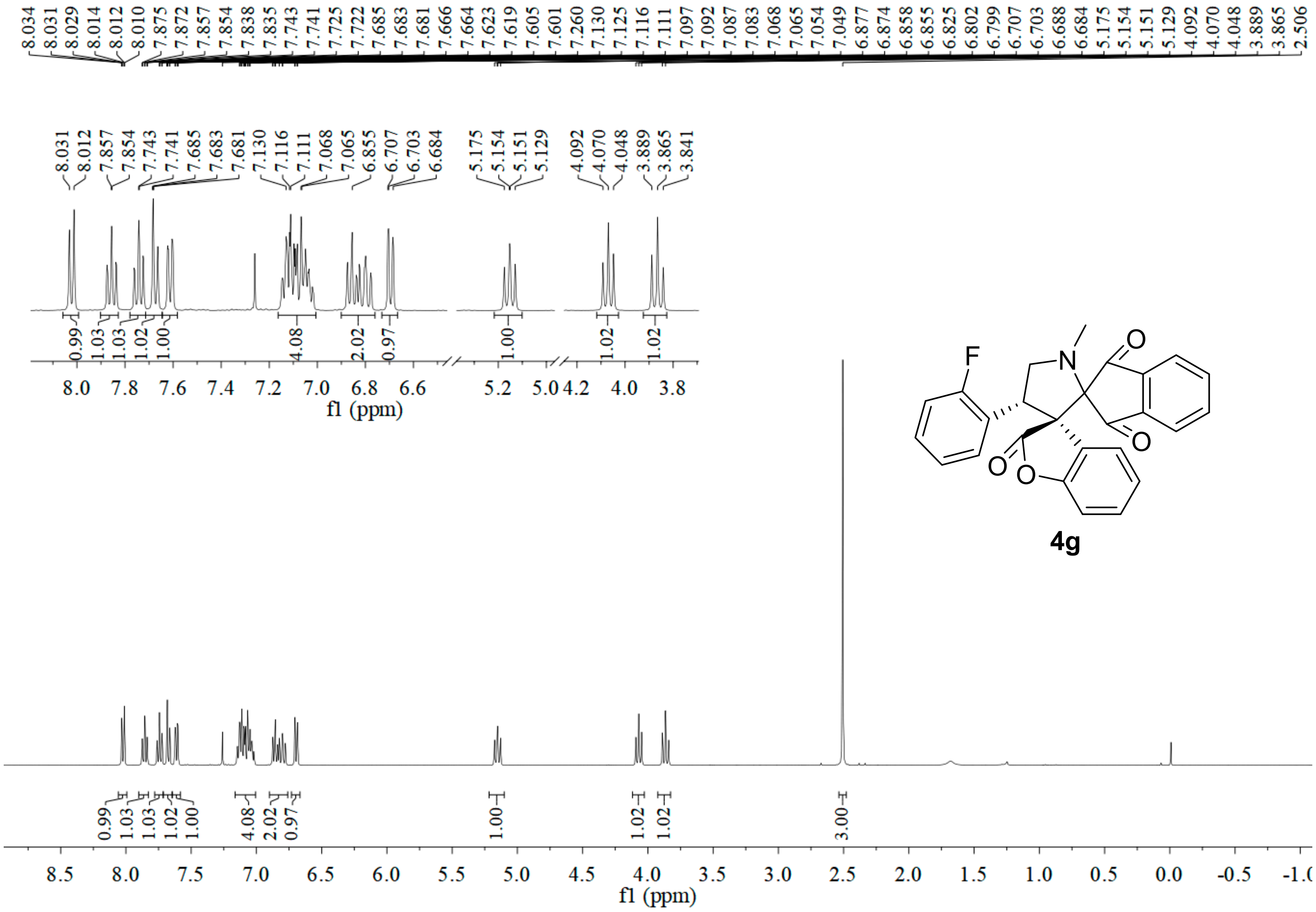

^{1}H NMR of Compound **4g** (400 MHz, $CDCl_3$)

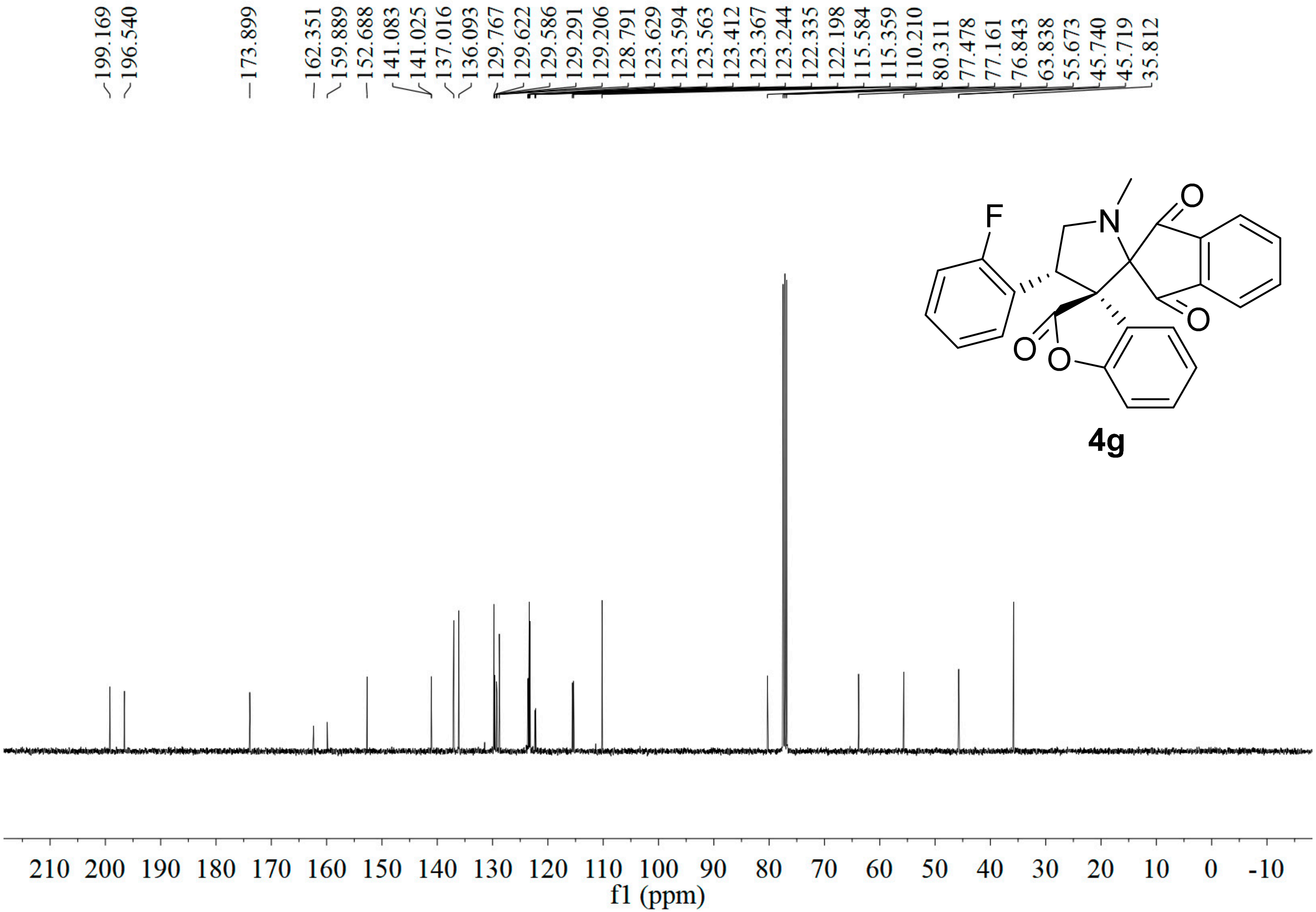

^{13}C NMR of Compound **4g** (100 MHz, $CDCl_3$)

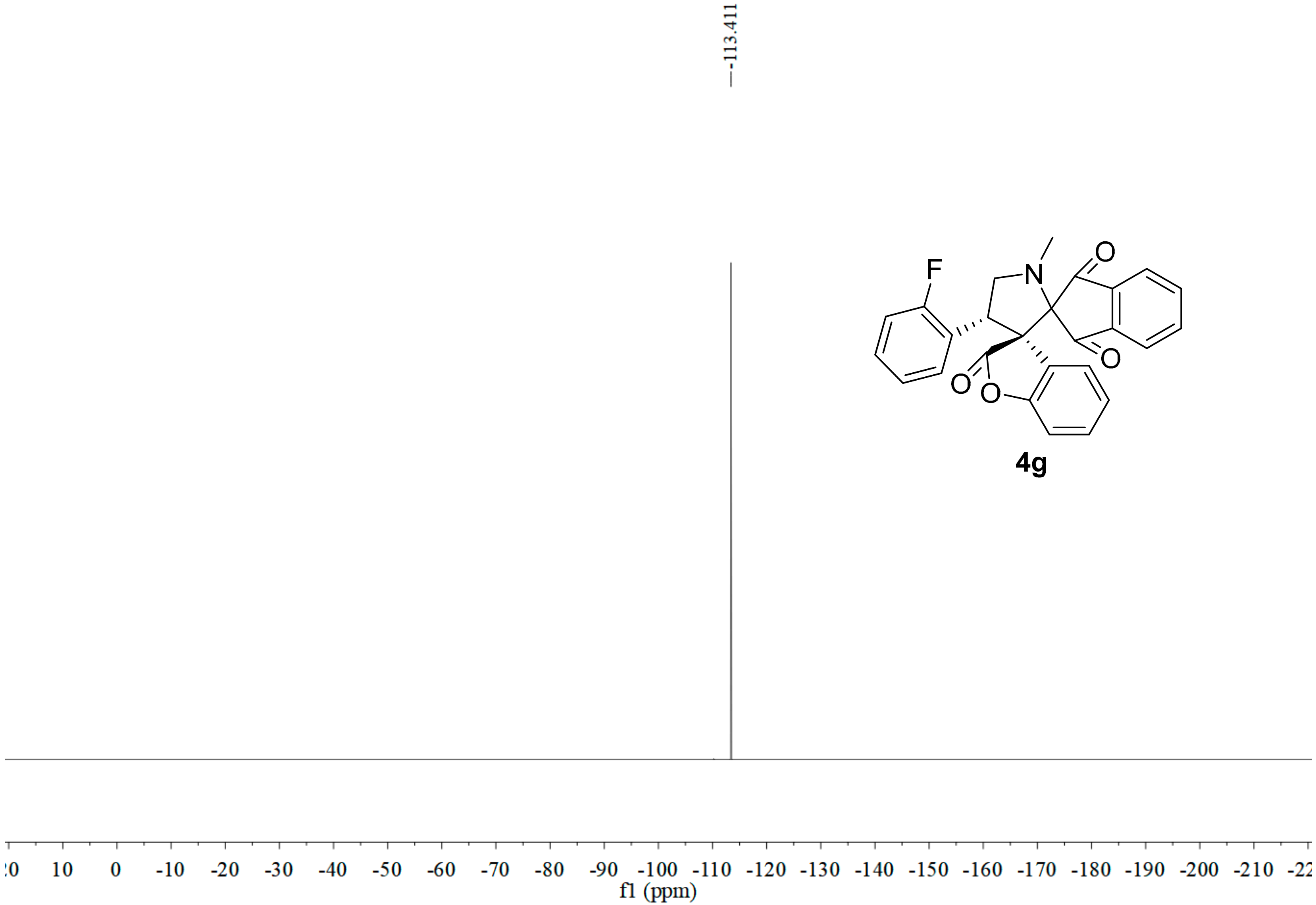

4g

^{19}F NMR of Compound **4g** (377 MHz, $CDCl_3$)

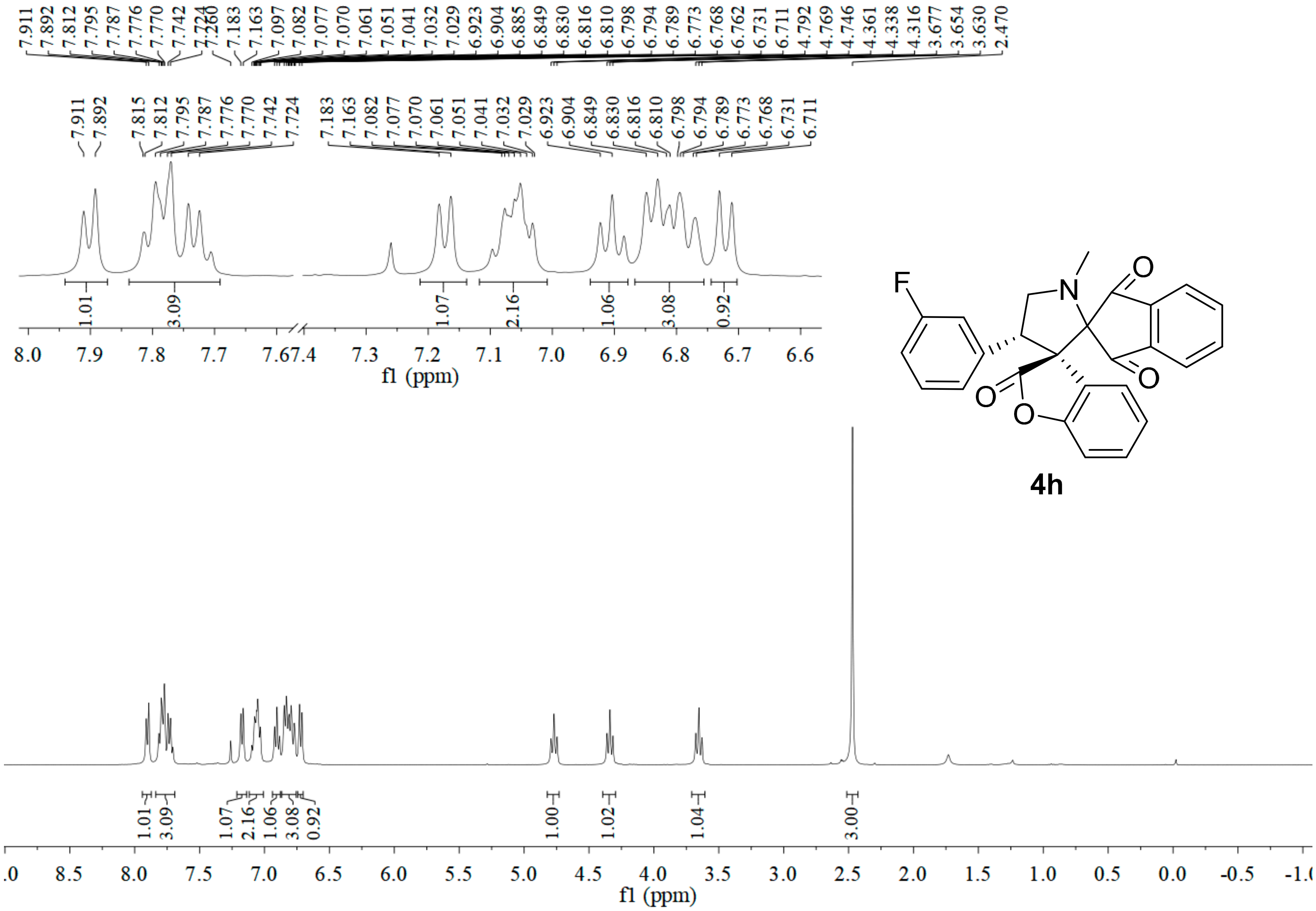

4h

^{1}H NMR of Compound **4h** (400 MHz, $CDCl_3$)

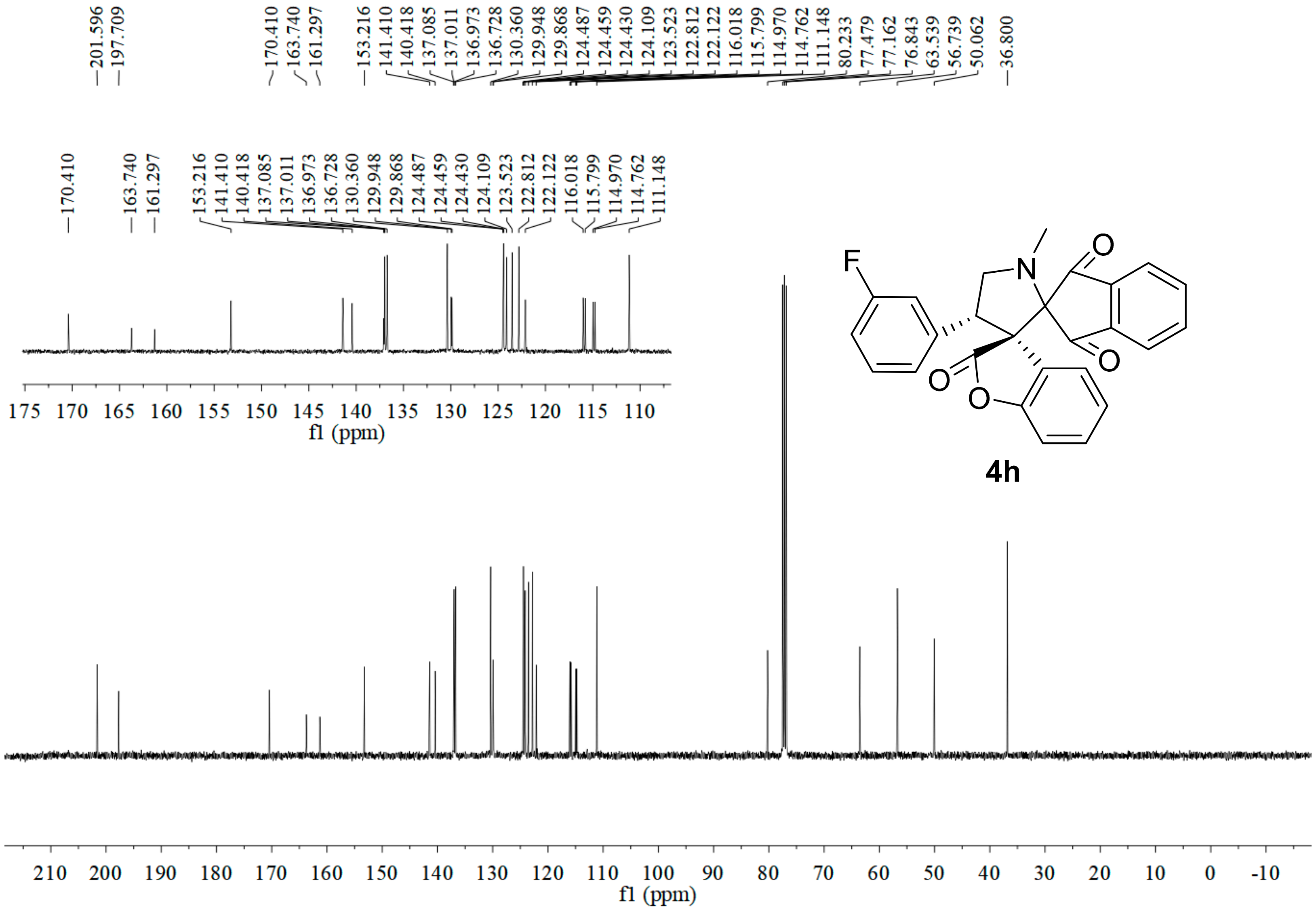

4h

^{13}C NMR of Compound **4h** (100 MHz, $CDCl_3$)

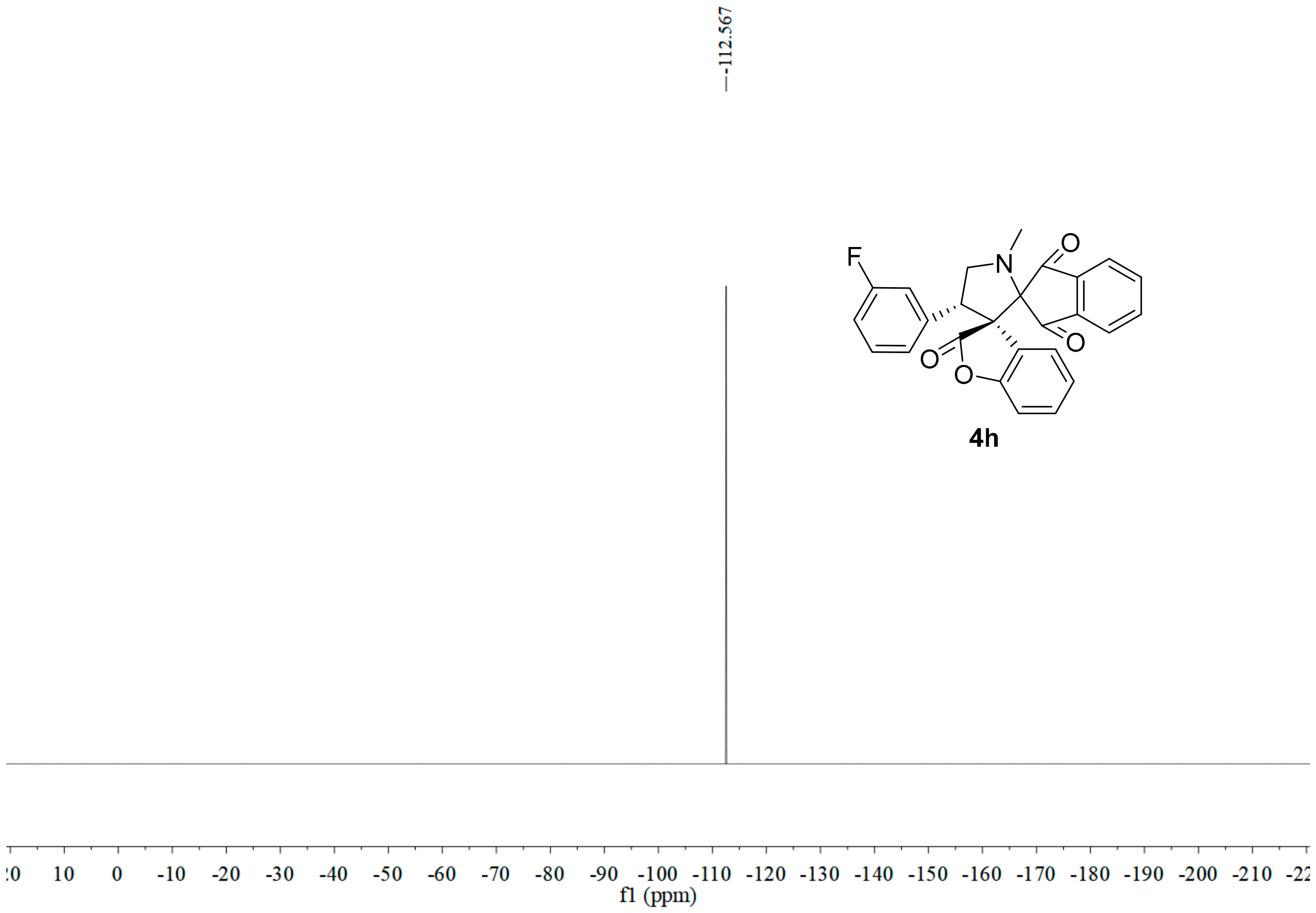

^{19}F NMR of Compound **4h** (377 MHz, $CDCl_3$)

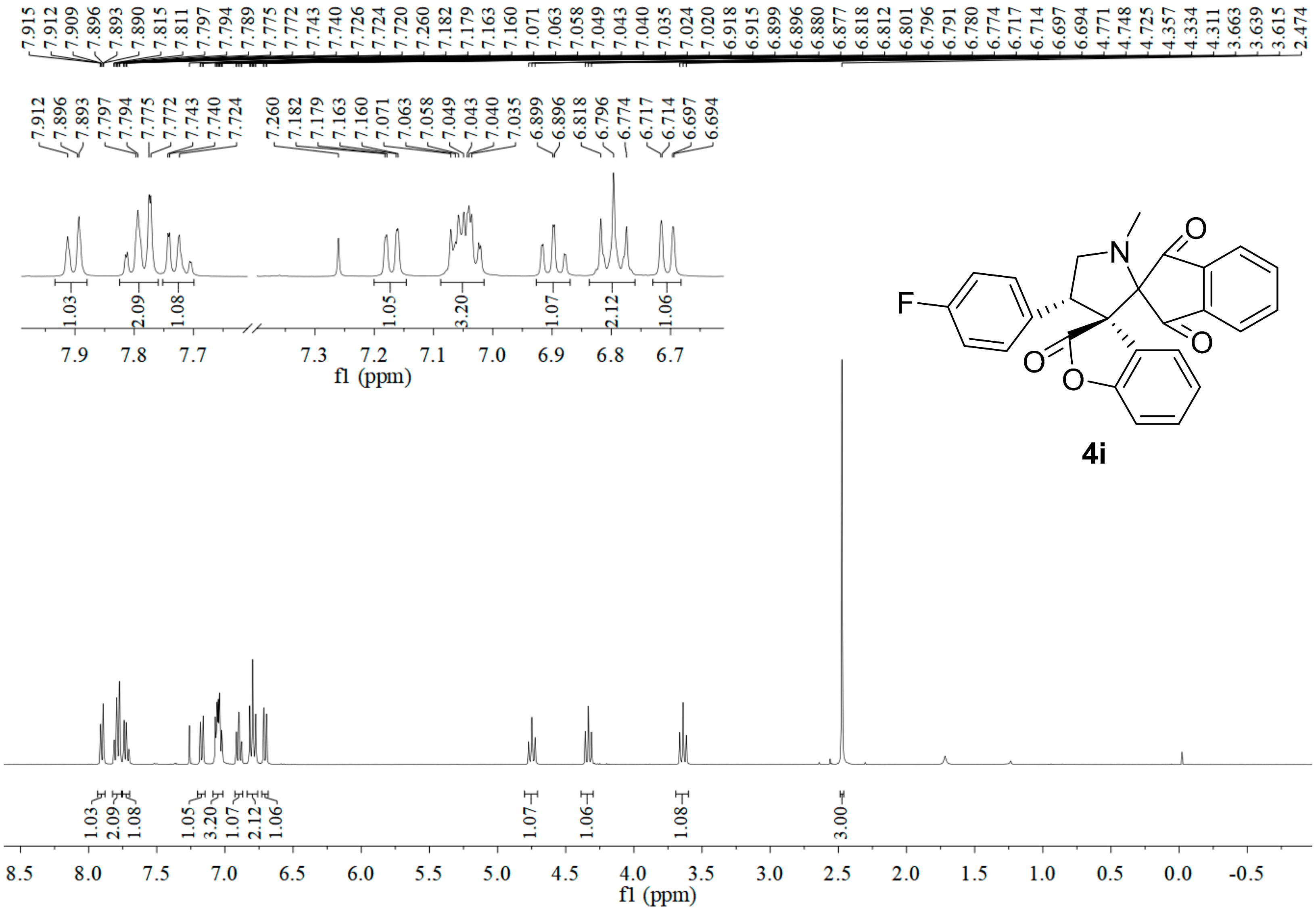

4i

^{1}H NMR of Compound **4i** (400 MHz, $CDCl_3$)

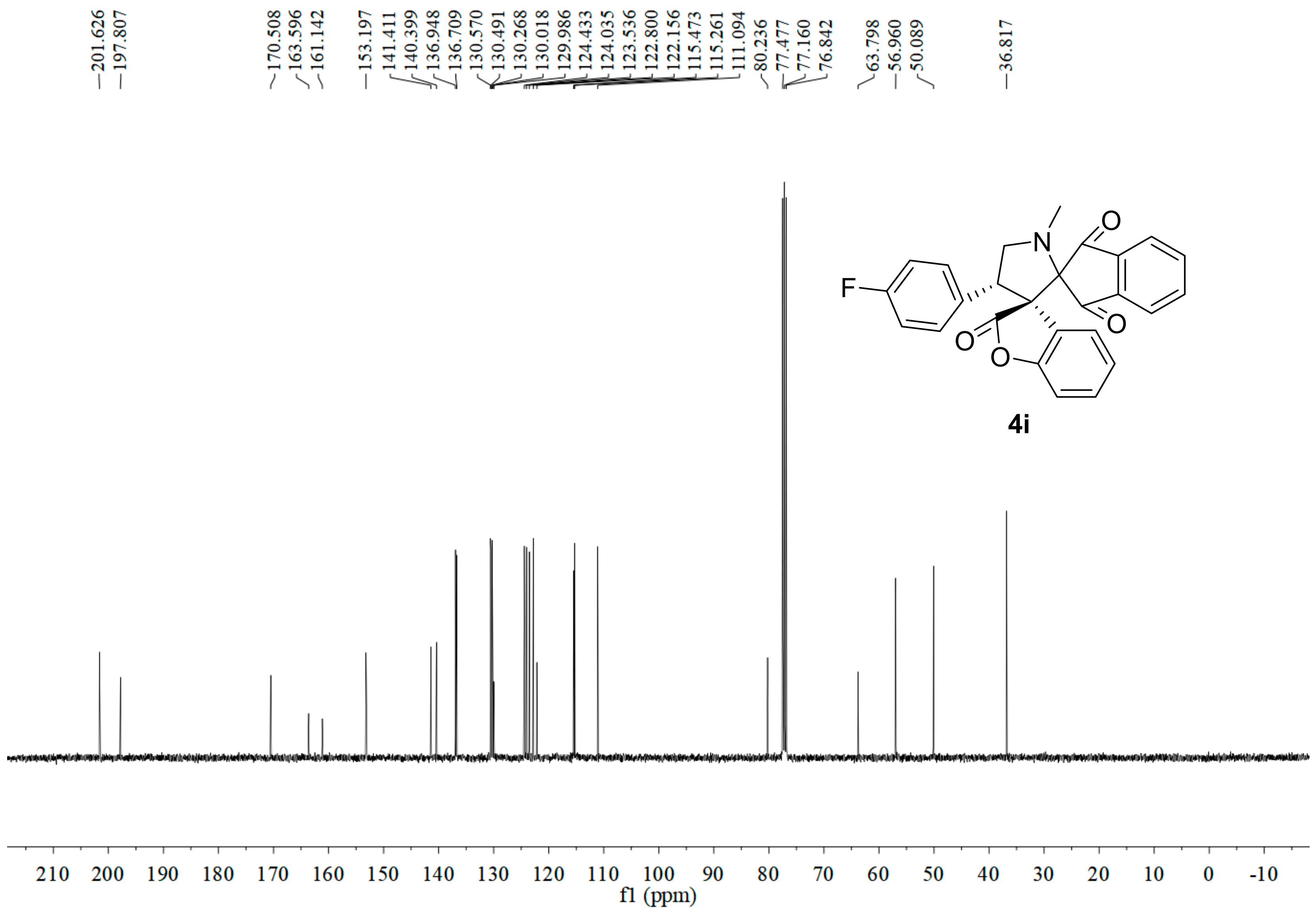

^{13}C NMR of Compound **4i** (100 MHz, $CDCl_3$)

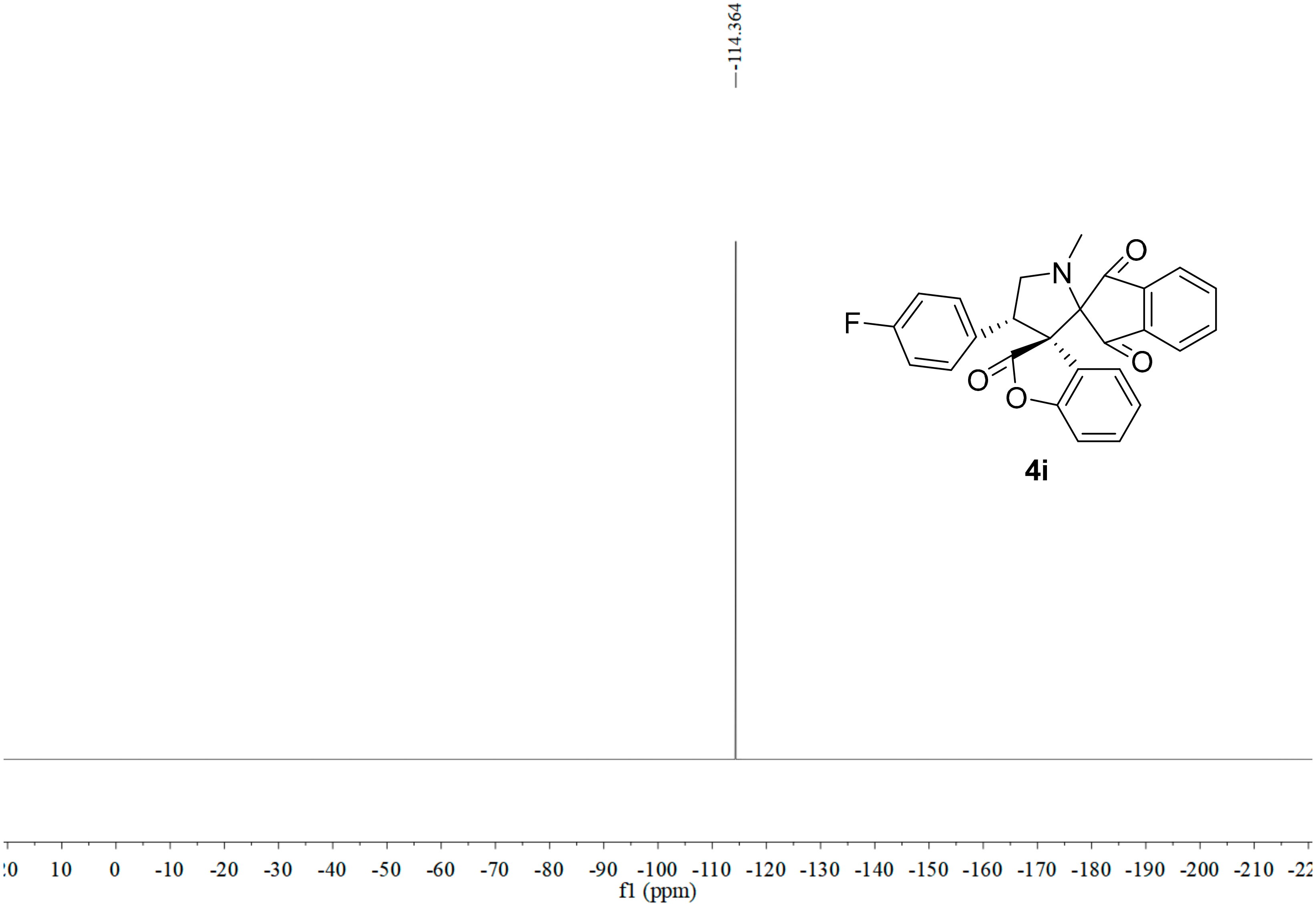

4i

^{19}F NMR of Compound **4i** (377 MHz, $CDCl_3$)

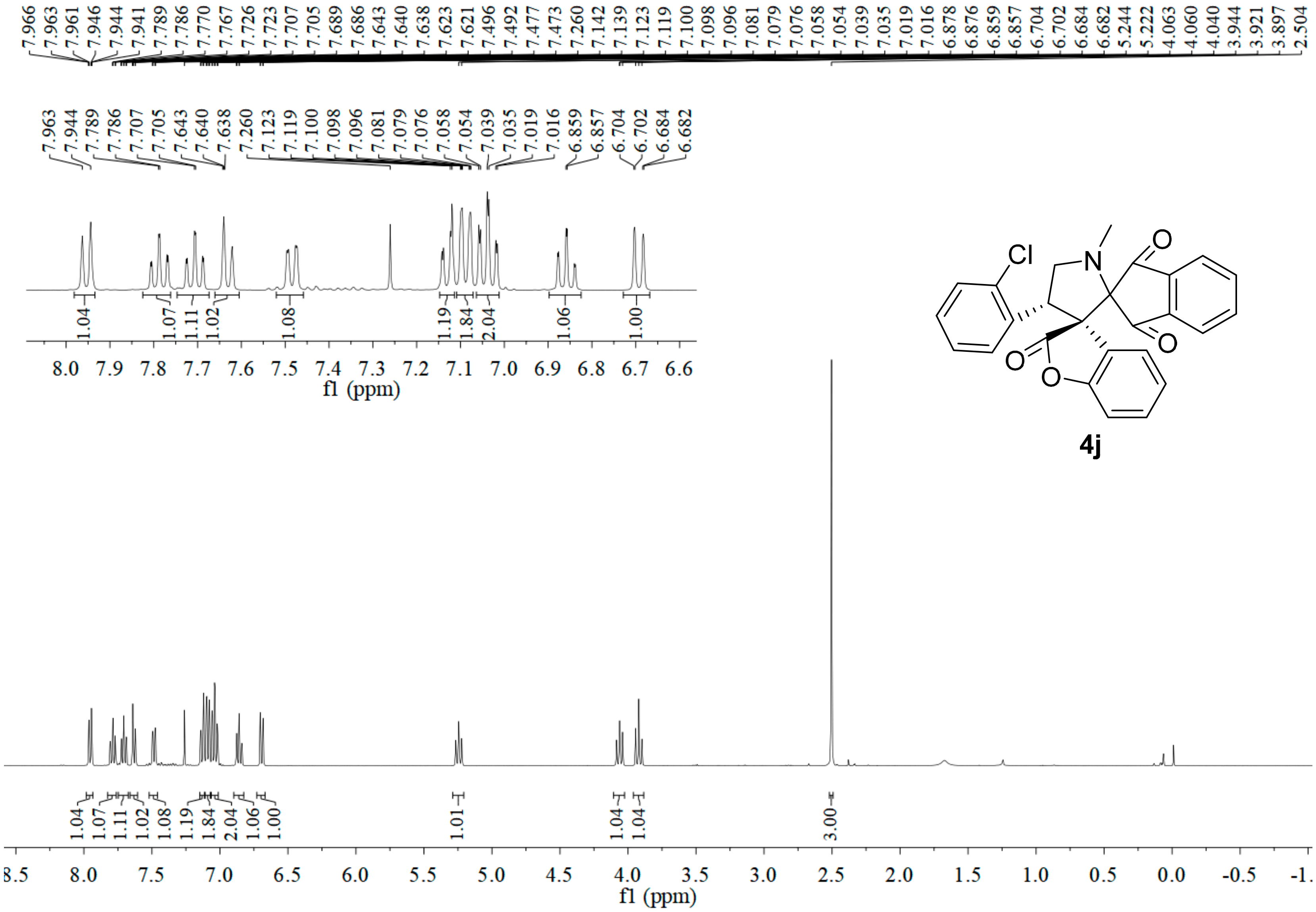

4j

^{1}H NMR of Compound **4j** (400 MHz, $CDCl_3$)

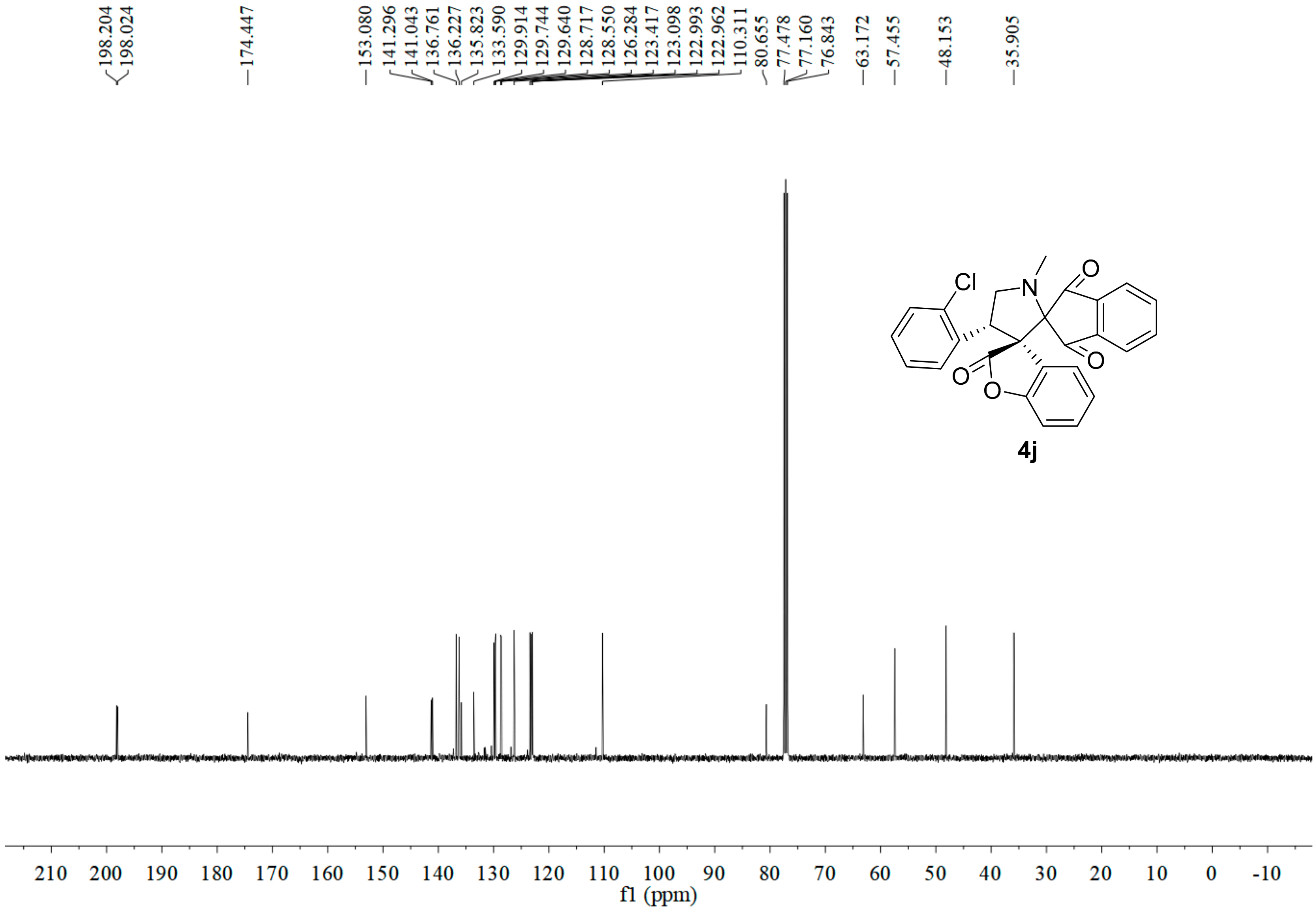

^{13}C NMR of Compound **4j** (100 MHz, $CDCl_3$)

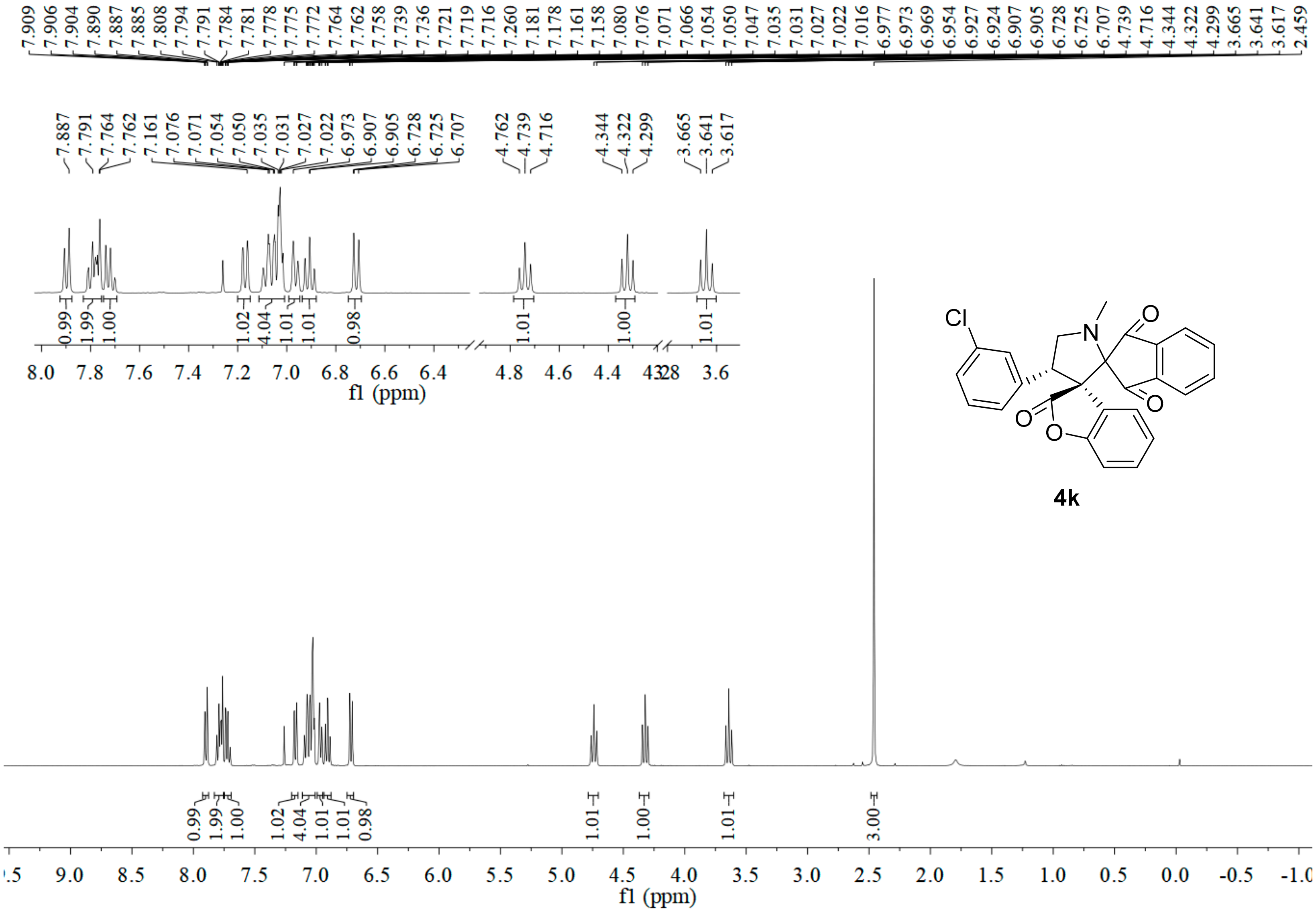

4k

^{1}H NMR of Compound **4k** (400 MHz, $CDCl_3$)

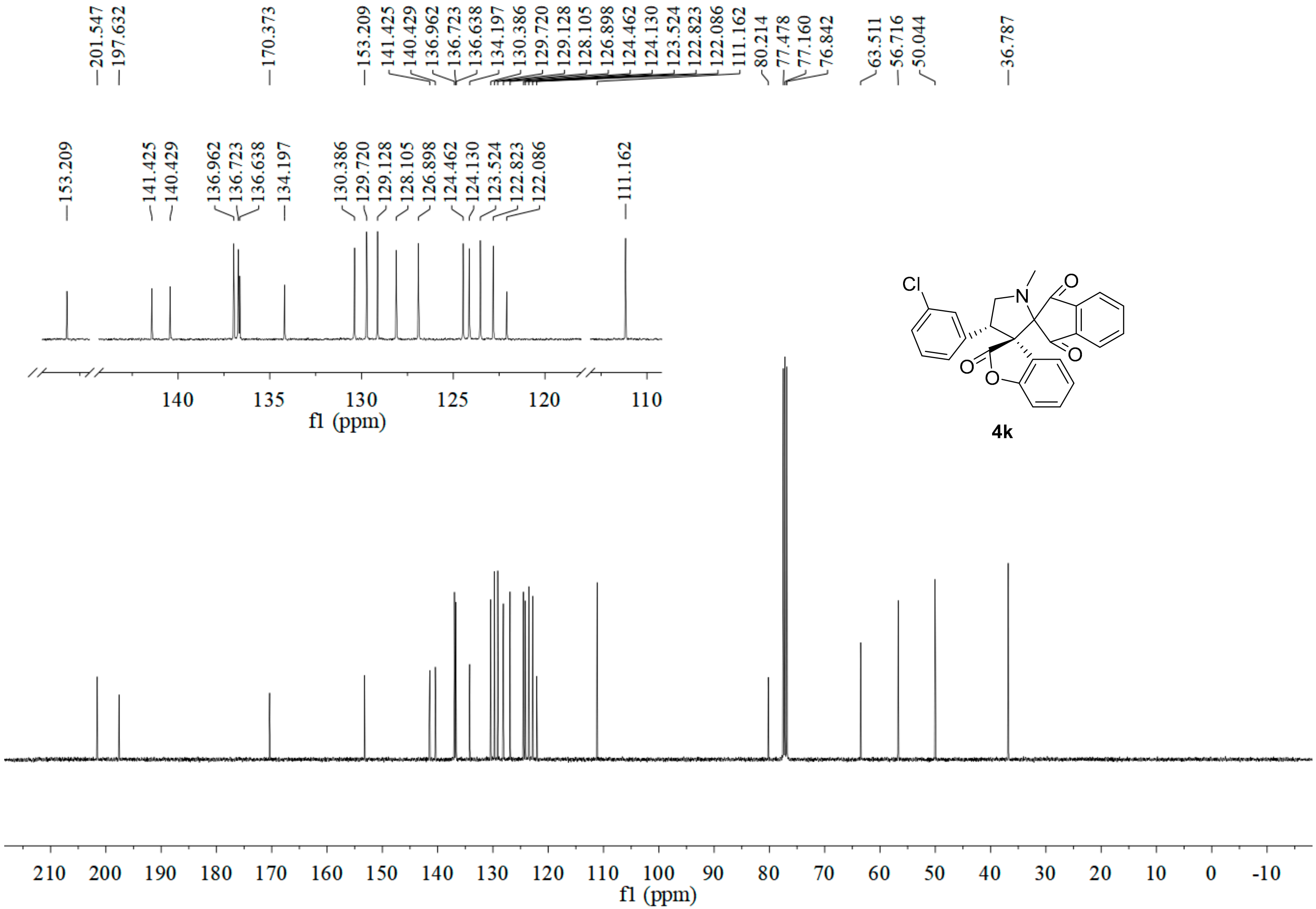

4k

^{13}C NMR of Compound **4k** (100 MHz, $CDCl_3$)

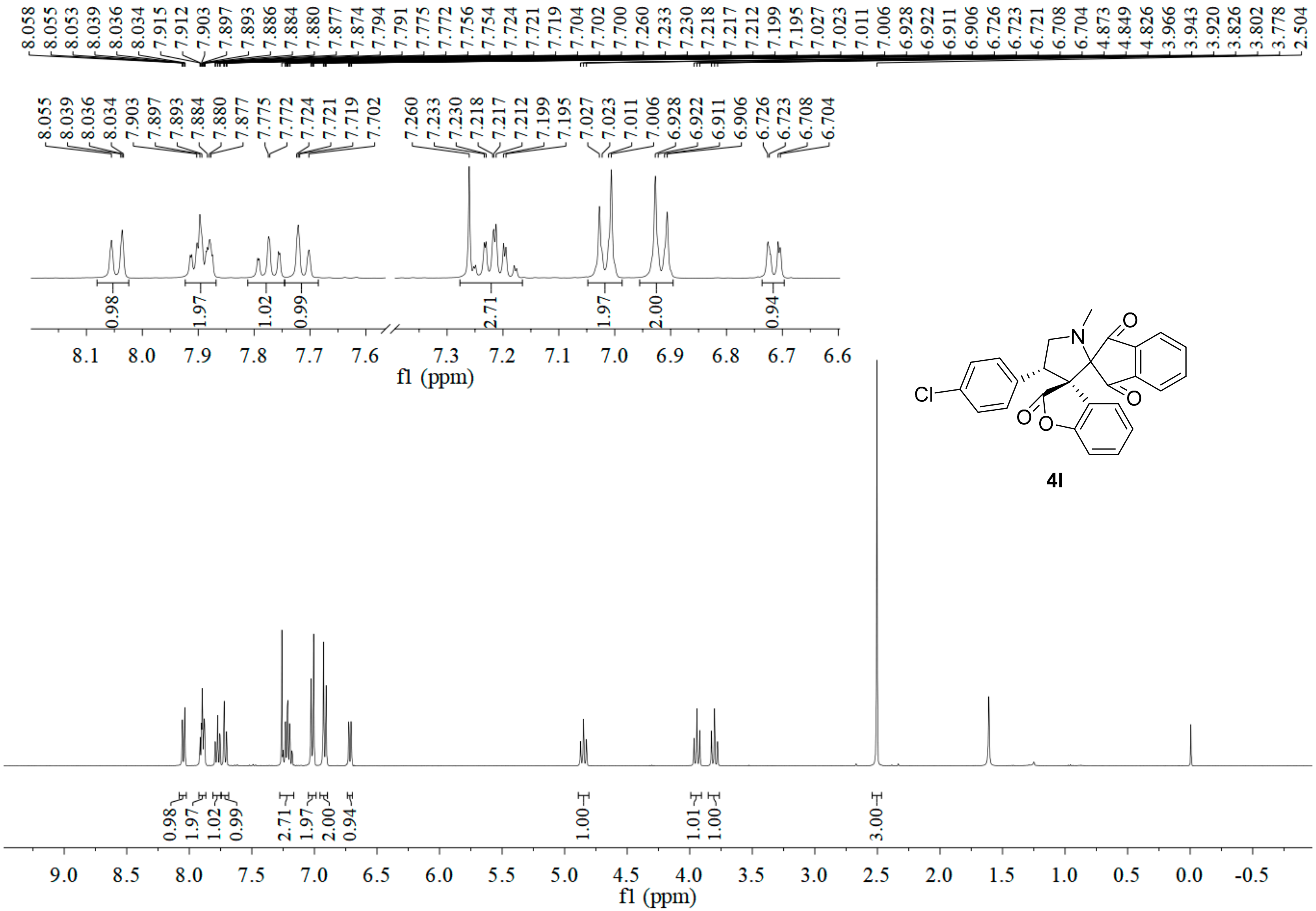

4l

^{1}H NMR of Compound **4l** (400 MHz, $CDCl_3$)

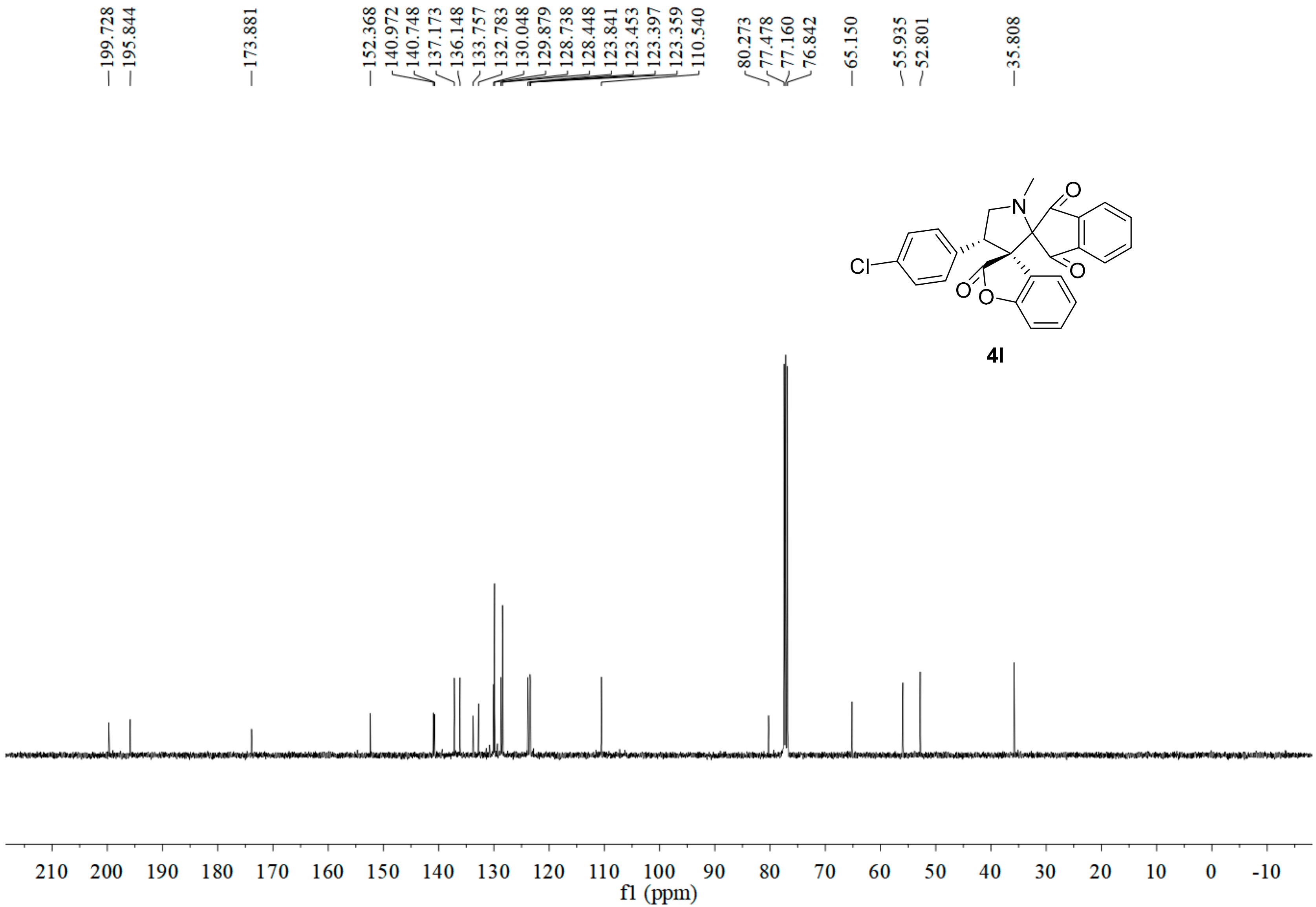

^{13}C NMR of Compound **4l** (100 MHz, $CDCl_3$)

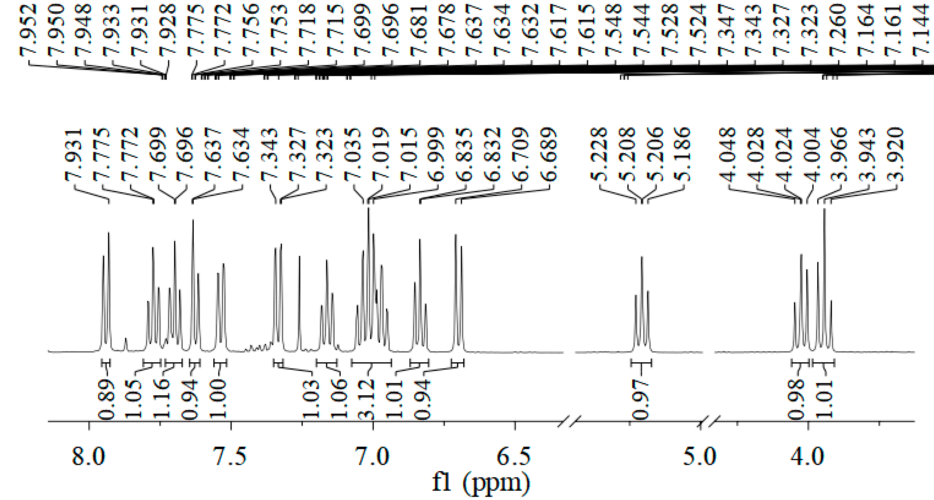

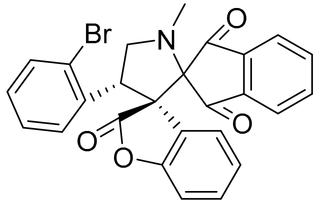

4m

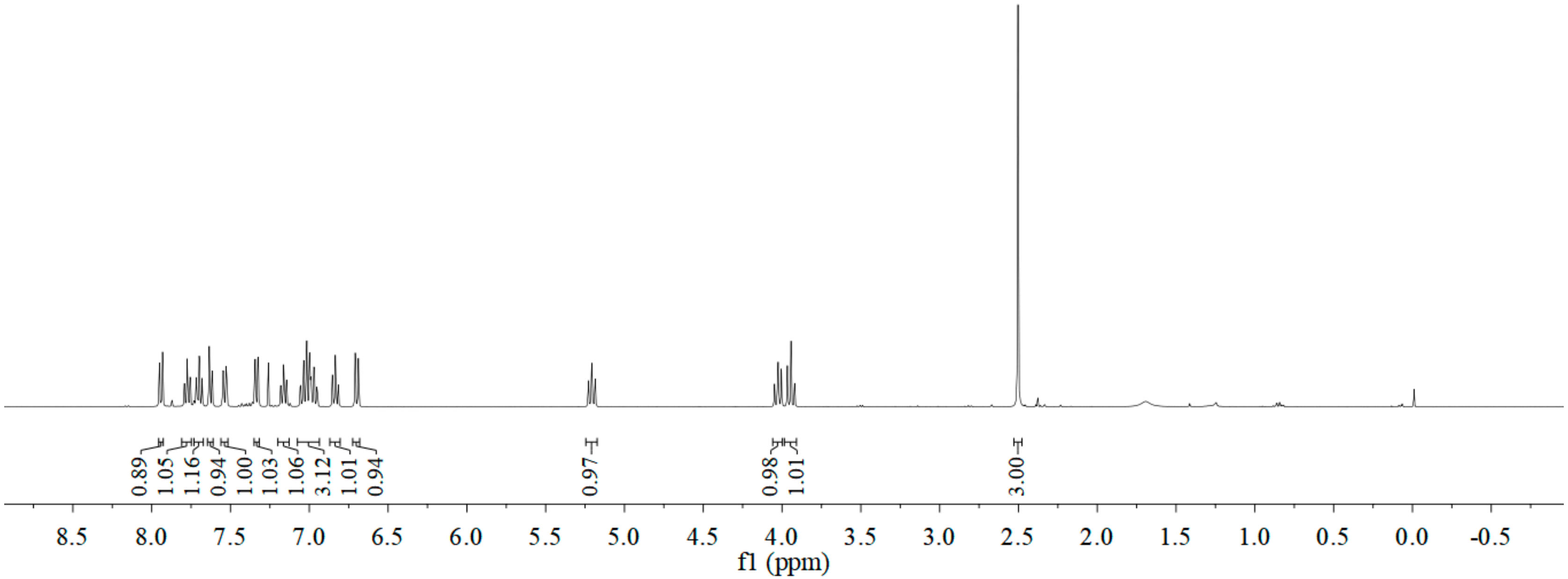

^{1}H NMR of Compound **4m** (400 MHz, $CDCl_3$)

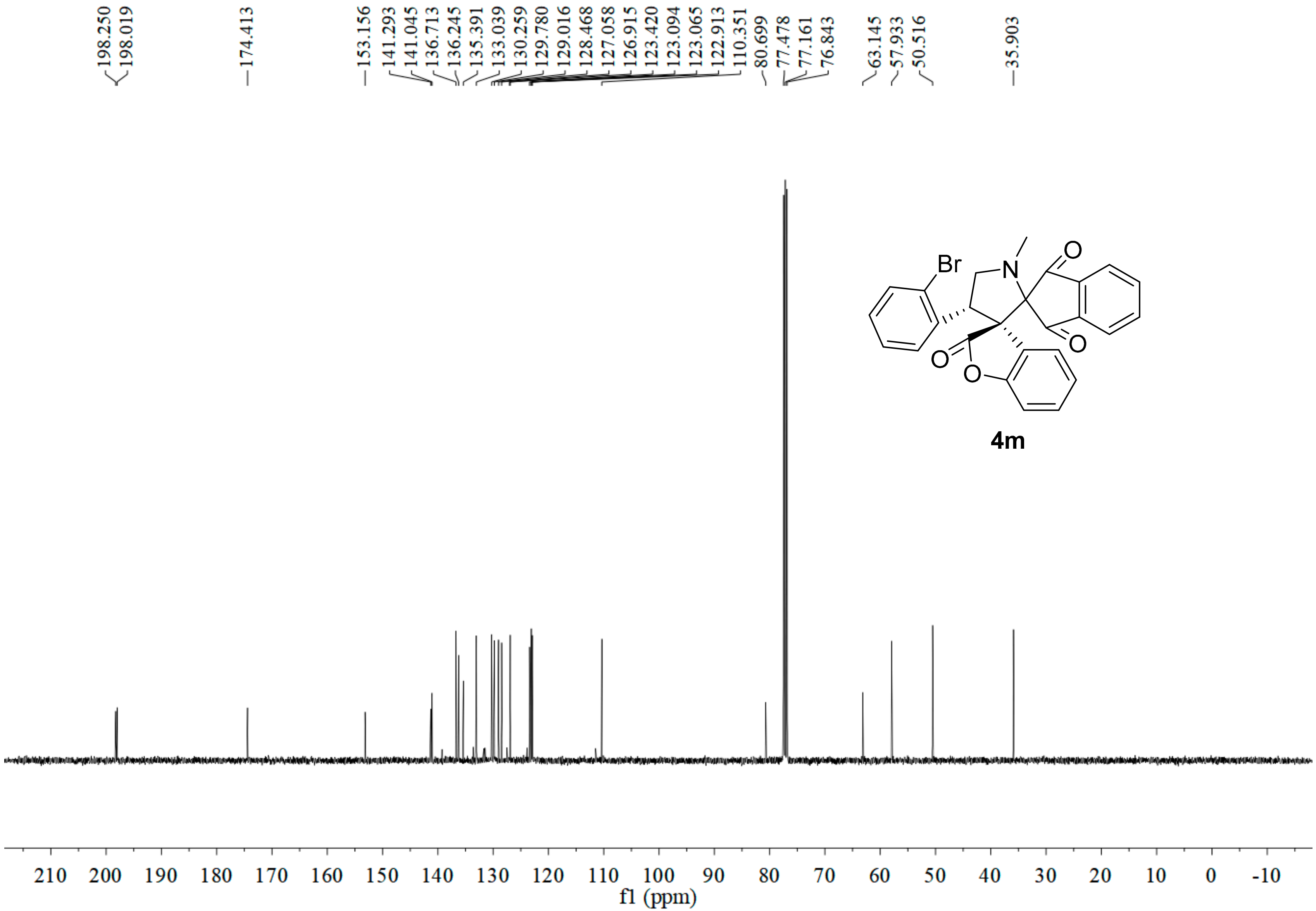

^{13}C NMR of Compound **4m** (100 MHz, $CDCl_3$)

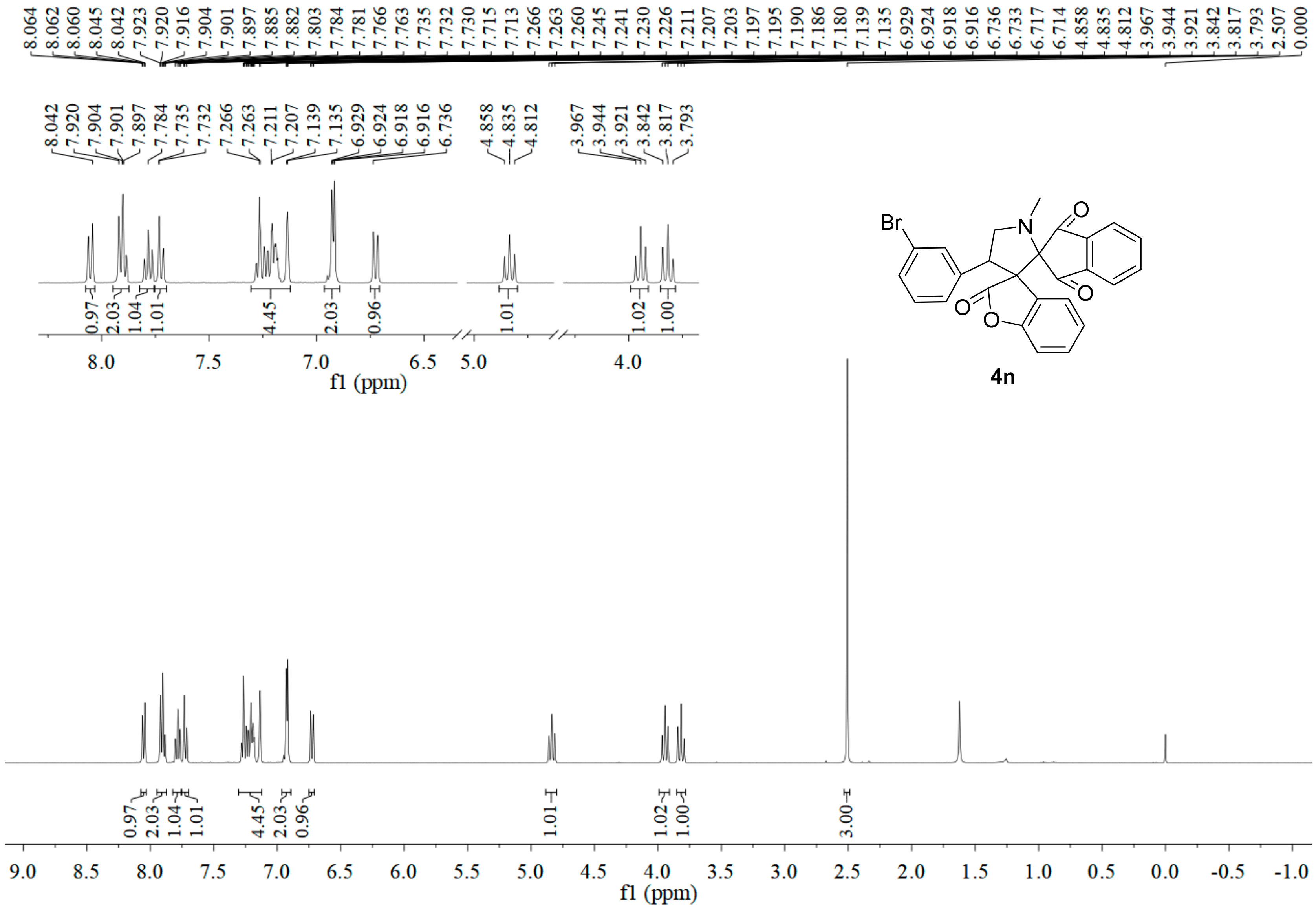

^{1}H NMR of Compound **4n** (400 MHz, $CDCl_3$)

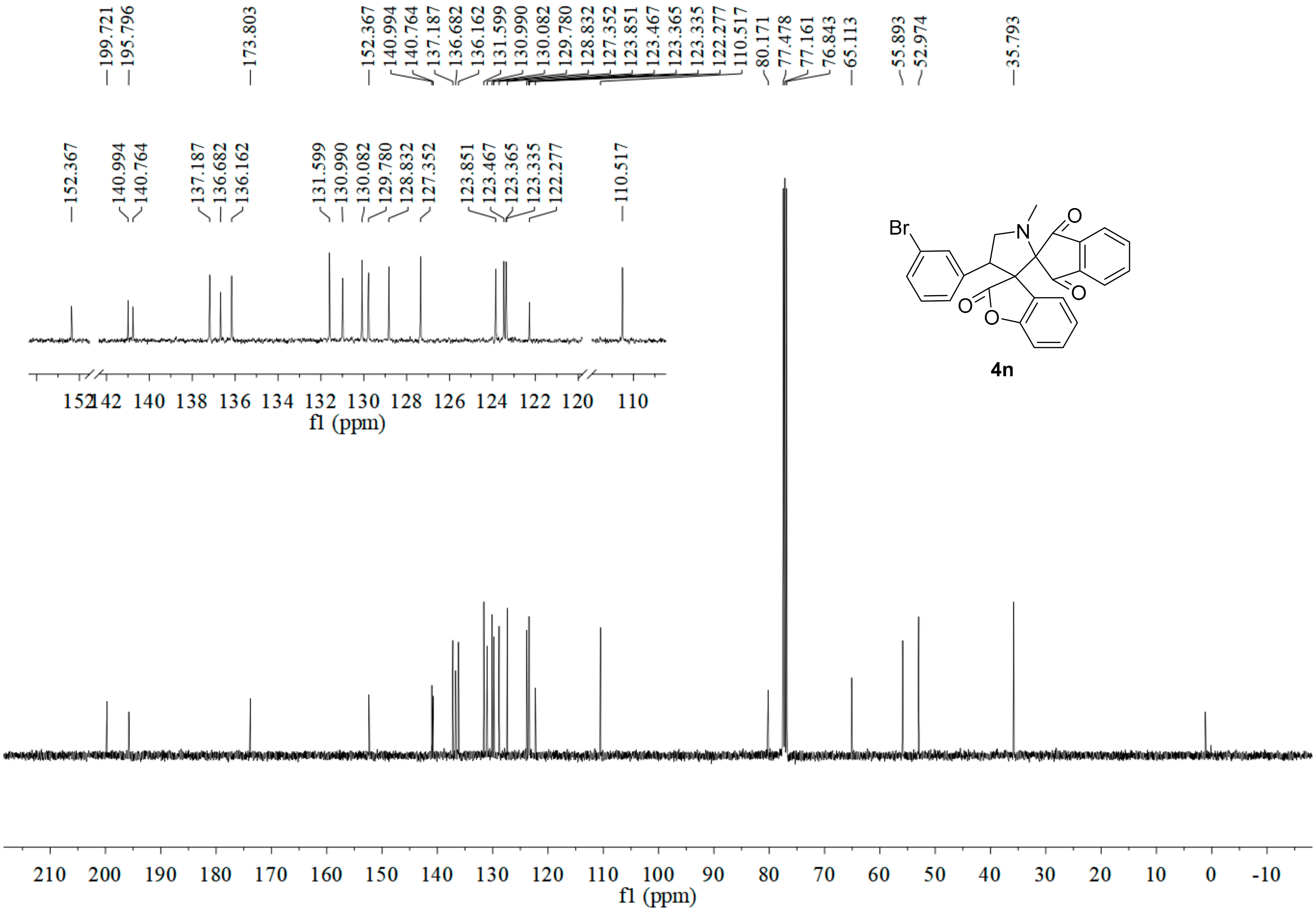

^{13}C NMR of Compound **4n** (100 MHz, $CDCl_3$)

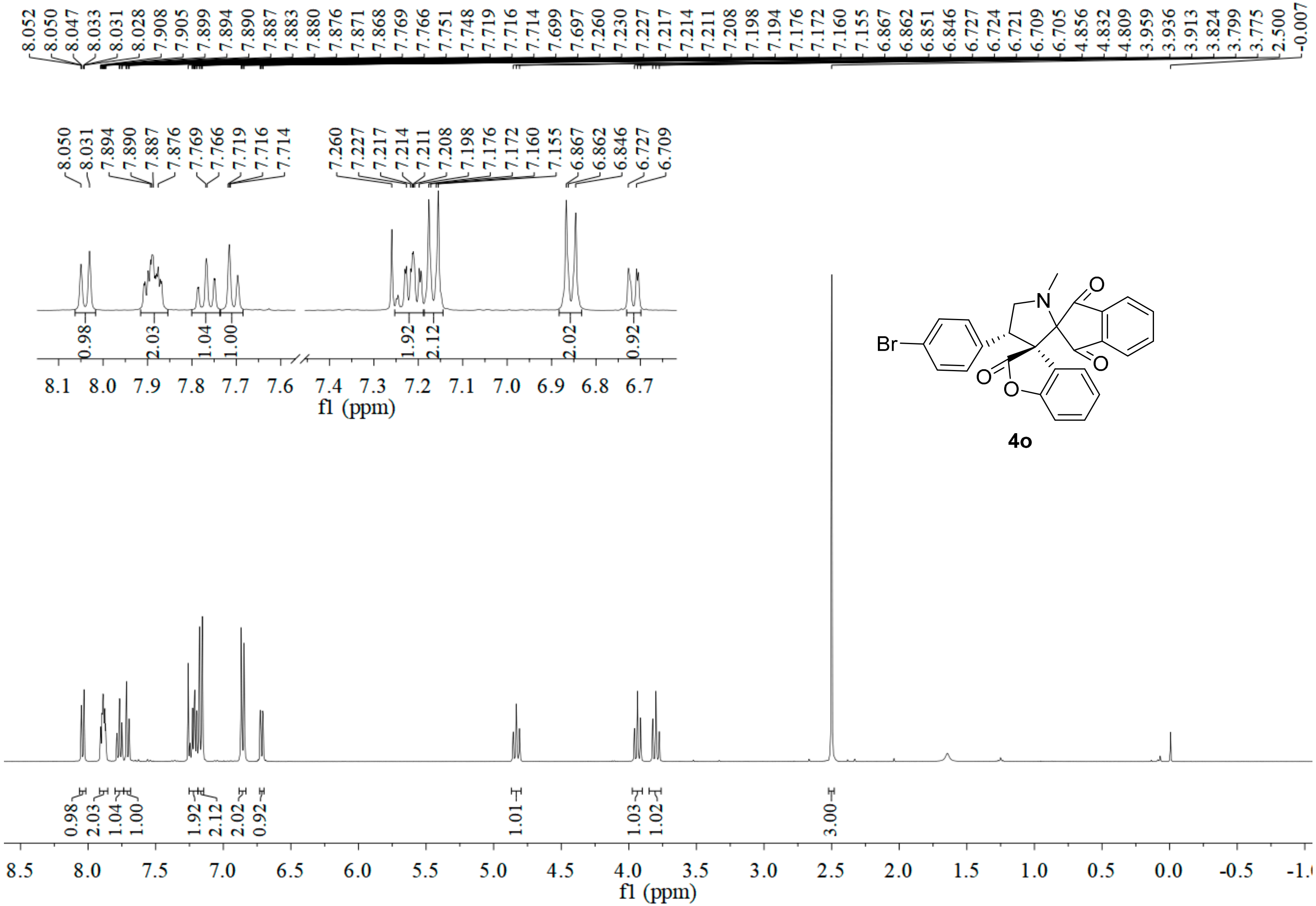

^{1}H NMR of Compound **4o** (400 MHz, $CDCl_3$)

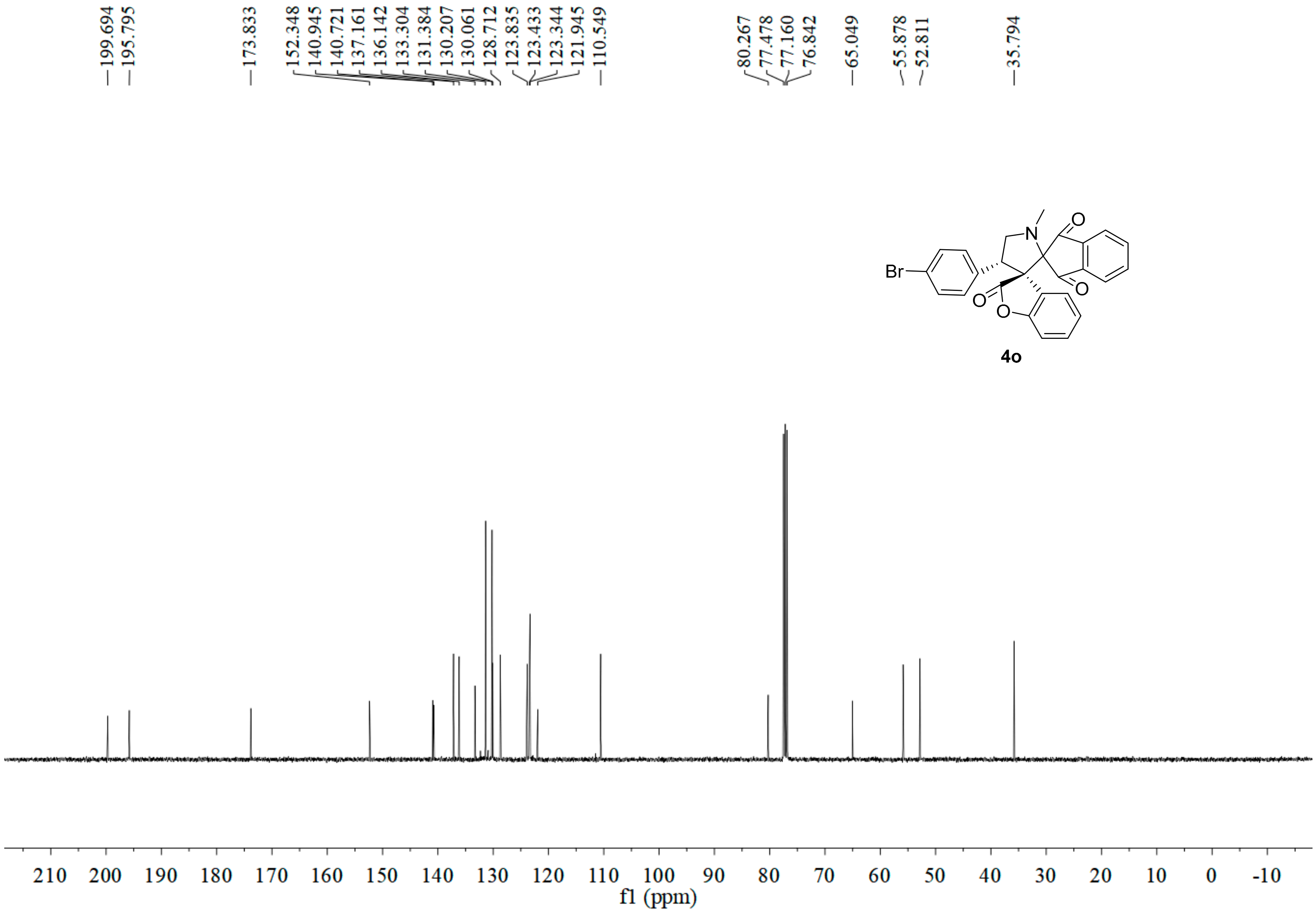

^{13}C NMR of Compound **4o** (100 MHz, CDCl$_3$)

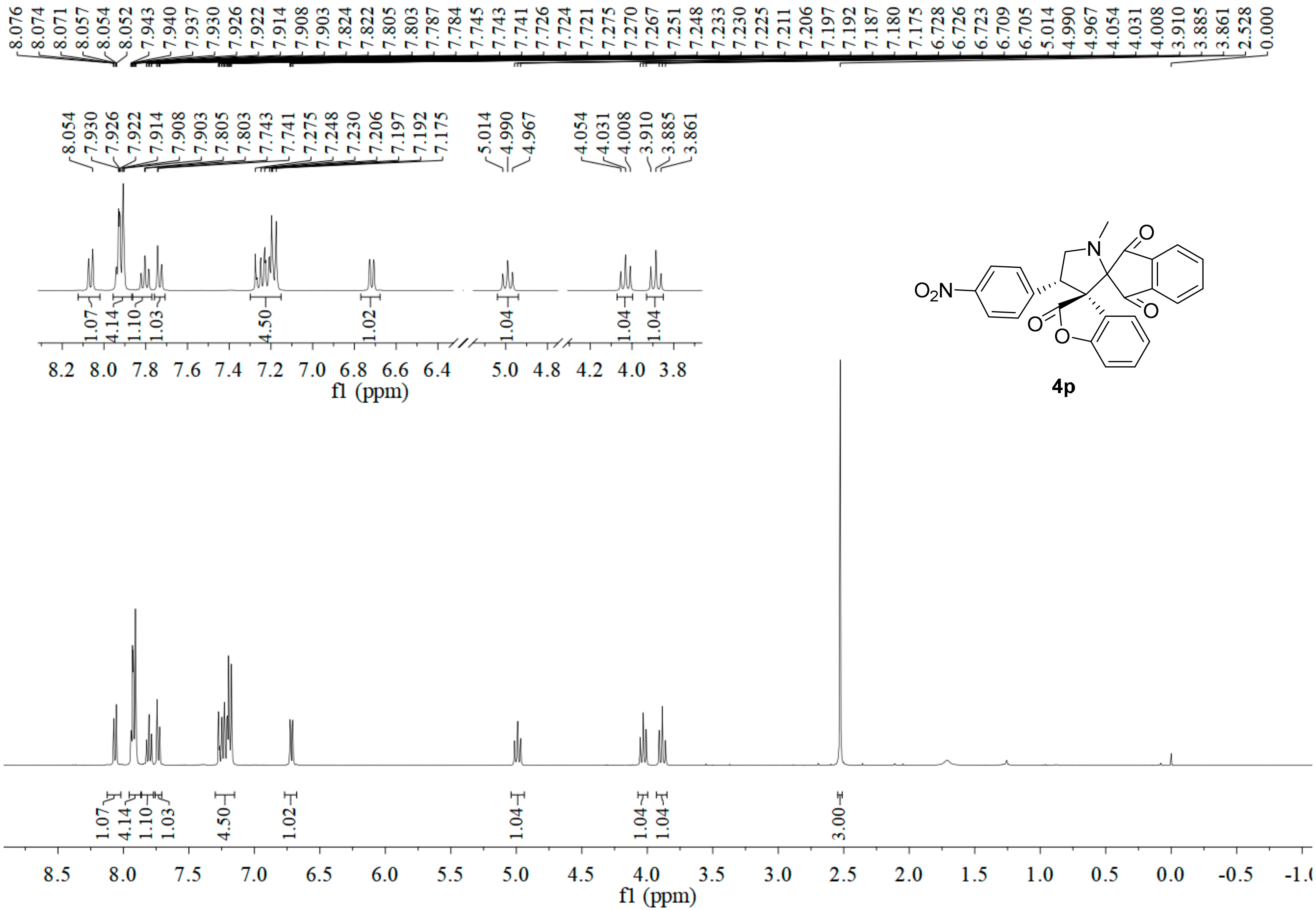

^{1}H NMR of Compound **4p** (400 MHz, $CDCl_3$)

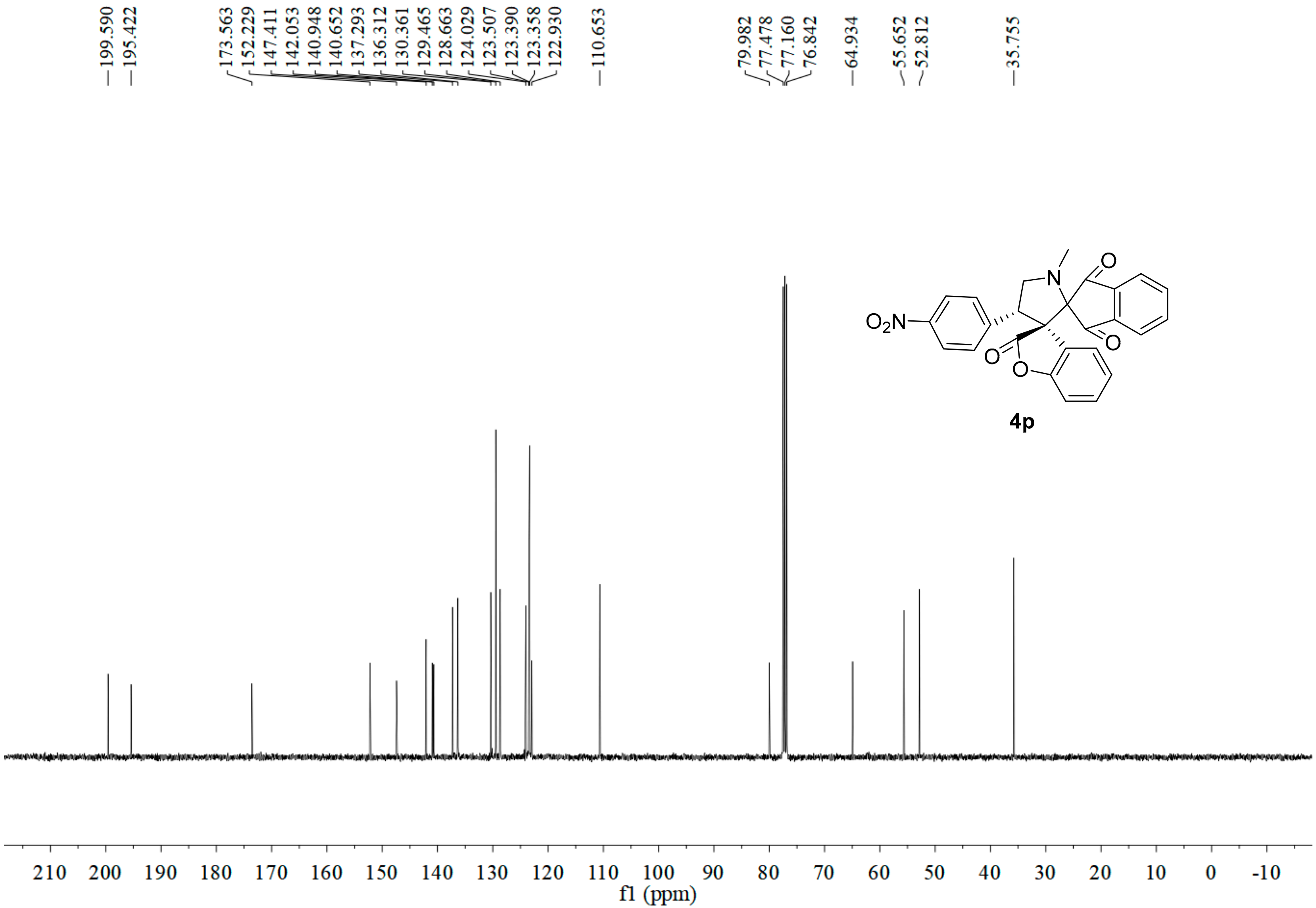

4p

^{13}C NMR of Compound **4p** (100 MHz, $CDCl_3$)

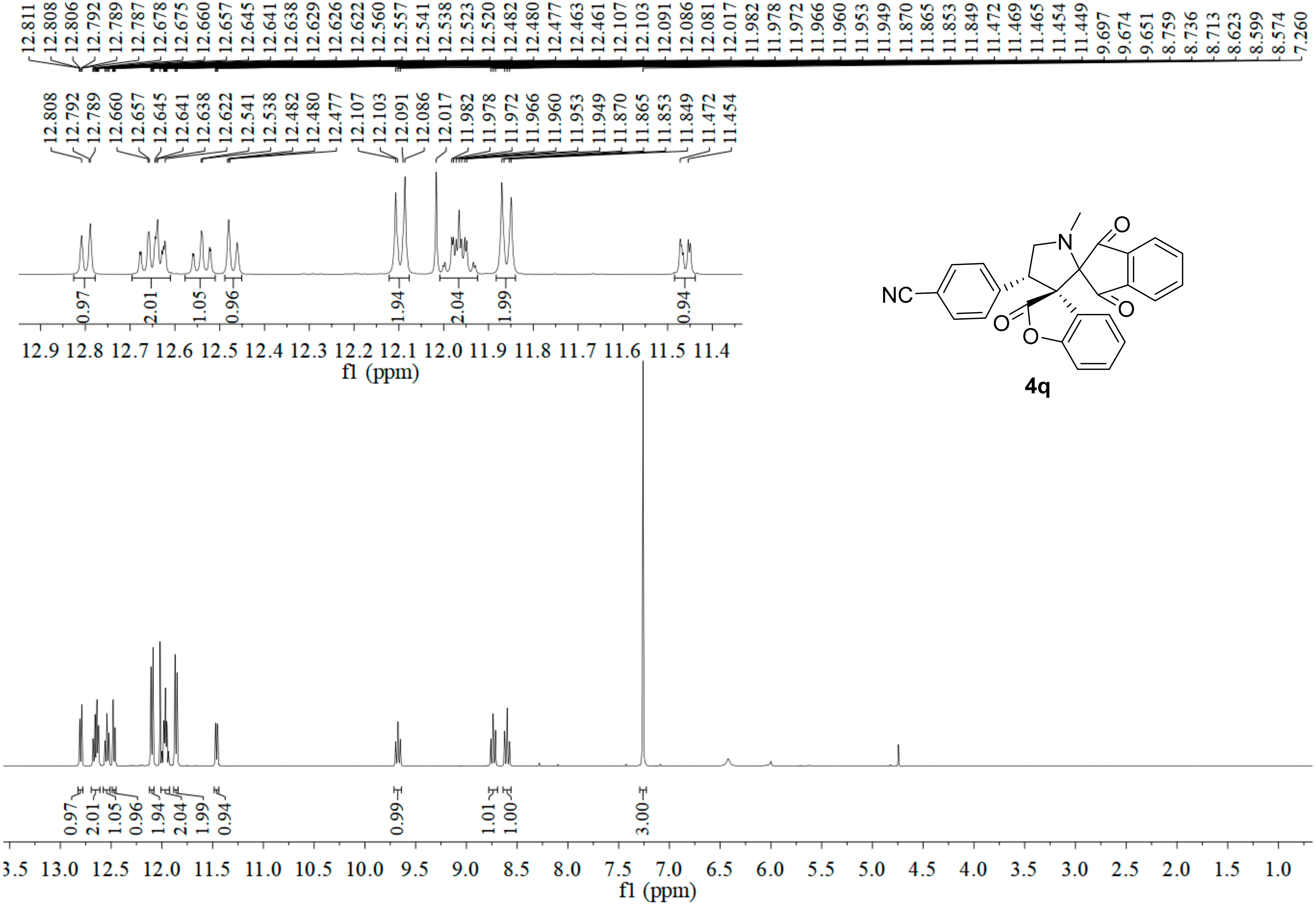

^{1}H NMR of Compound **4q** (400 MHz, $CDCl_3$)

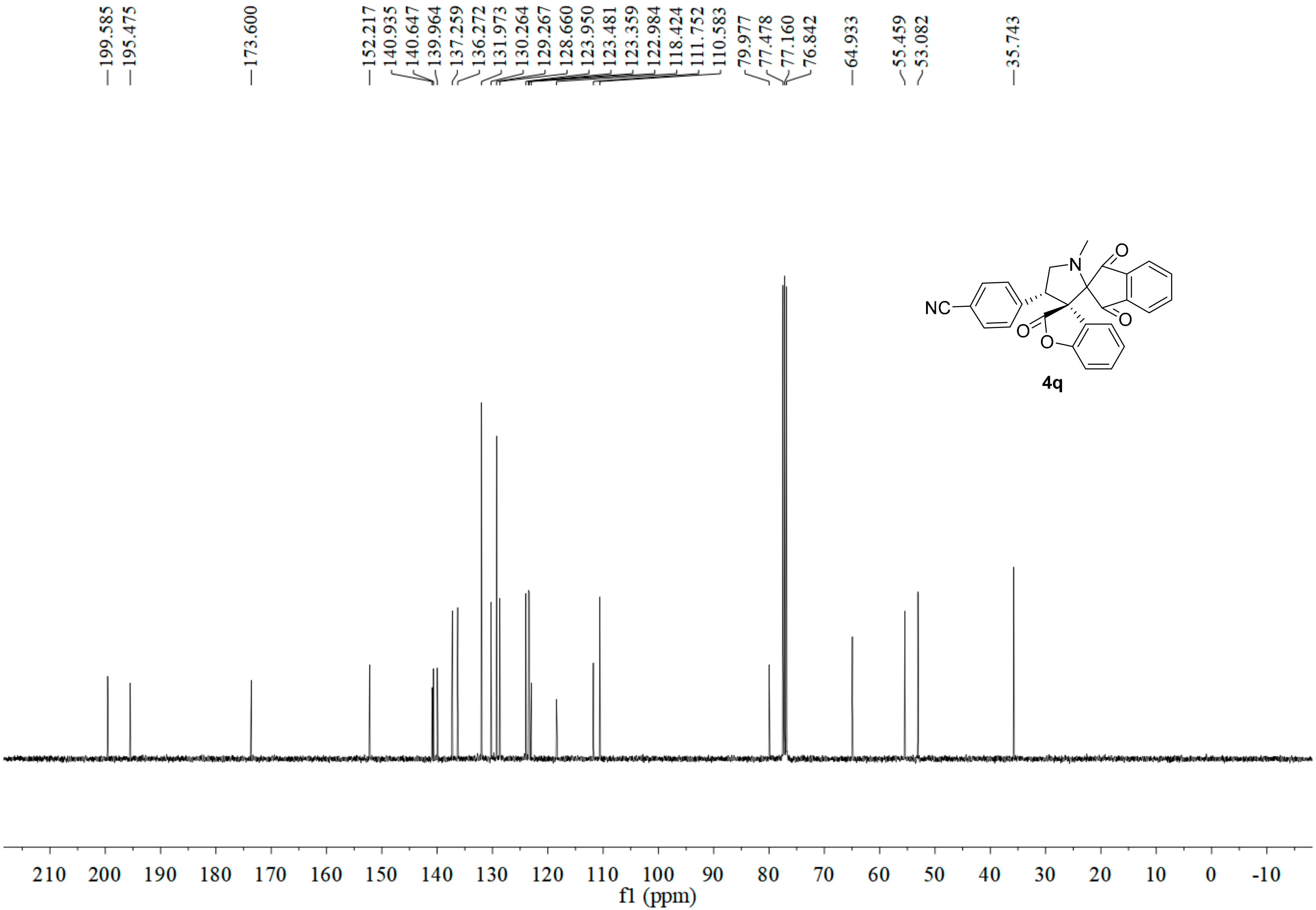

^{13}C NMR of Compound **4q** (100 MHz, $CDCl_3$)

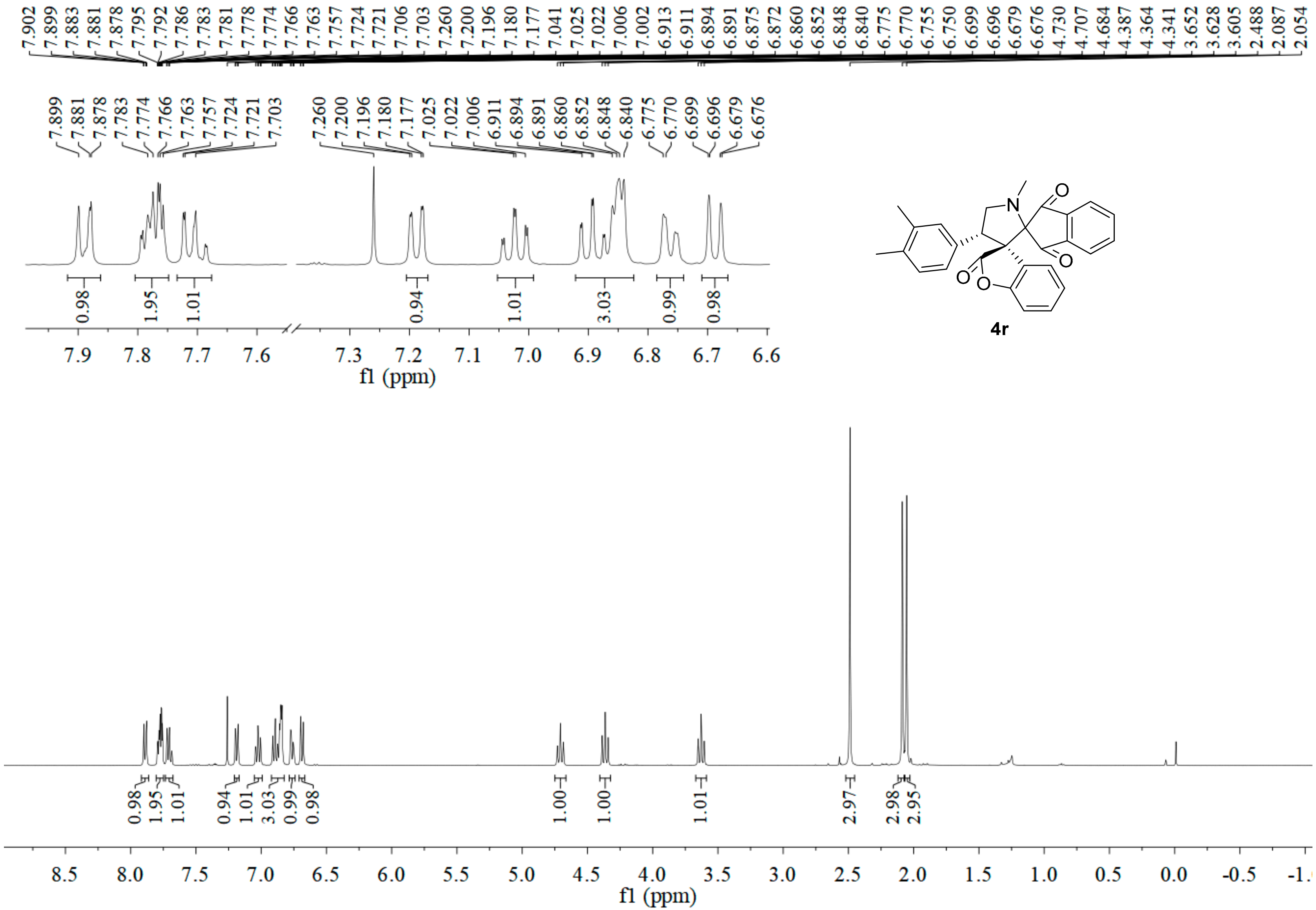

^{1}H NMR of Compound **4r** (400 MHz, $CDCl_3$)

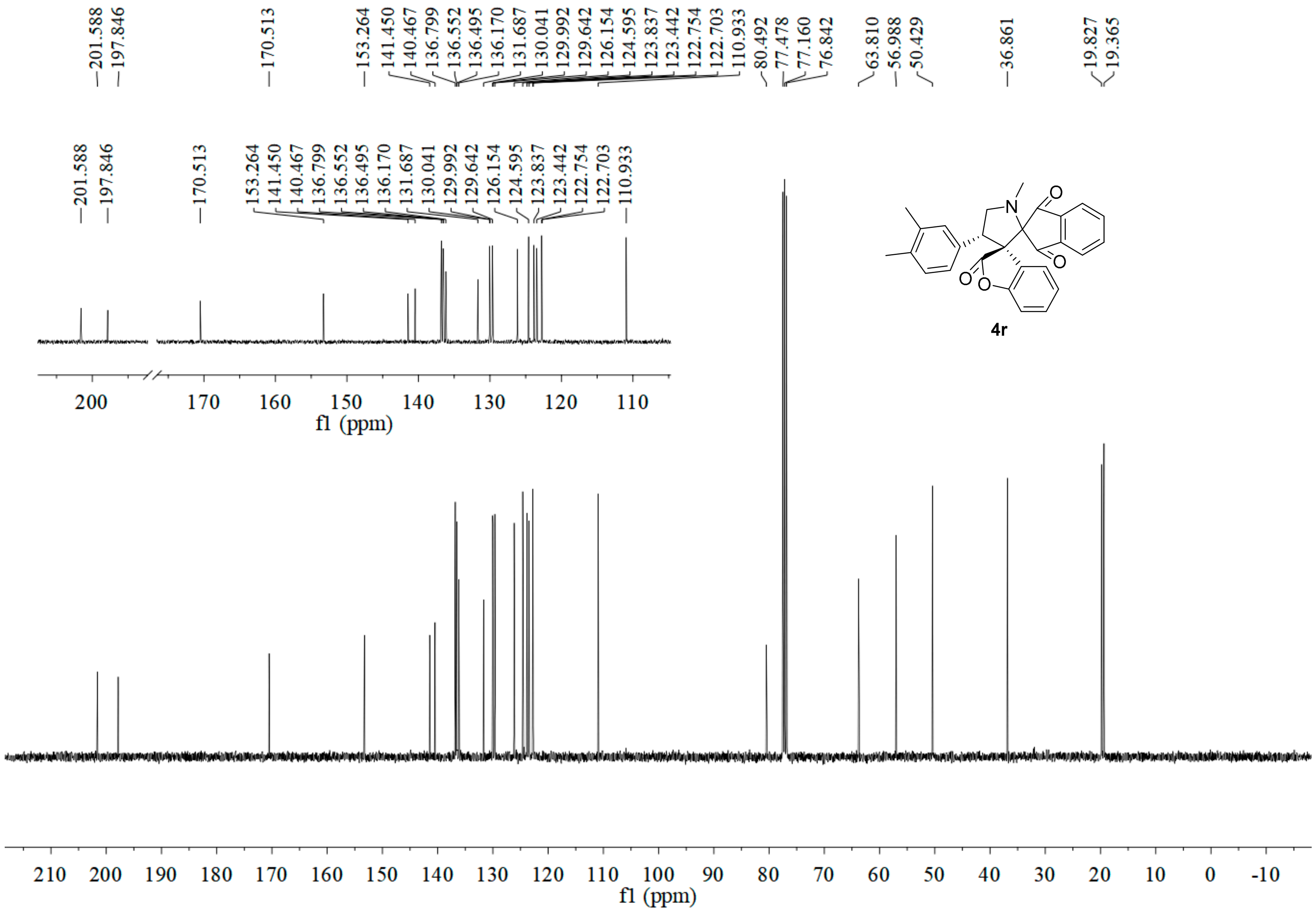

^{13}C NMR of Compound **4r** (100 MHz, $CDCl_3$)

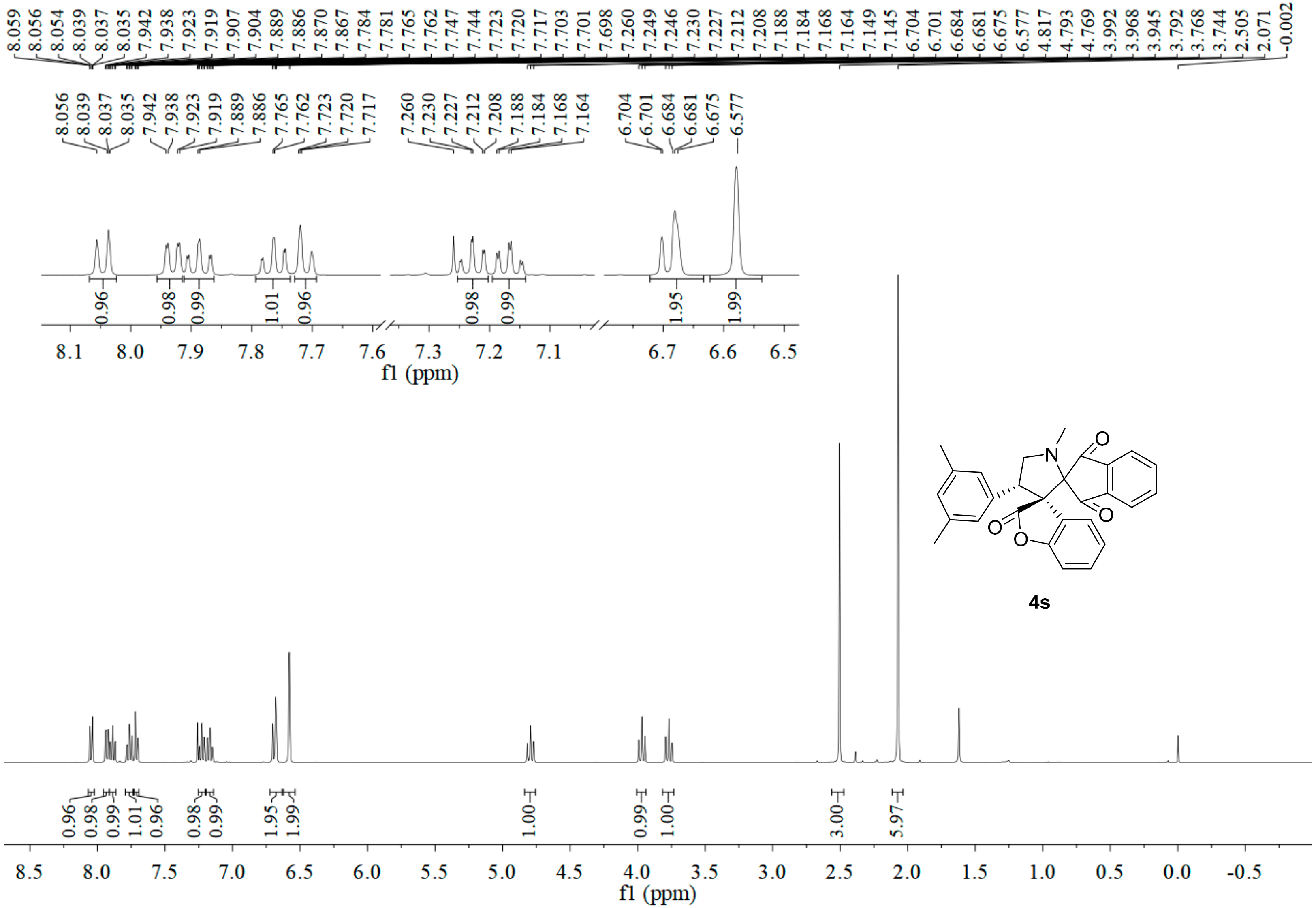

4s

^{1}H NMR of Compound **4s** (400 MHz, $CDCl_3$)

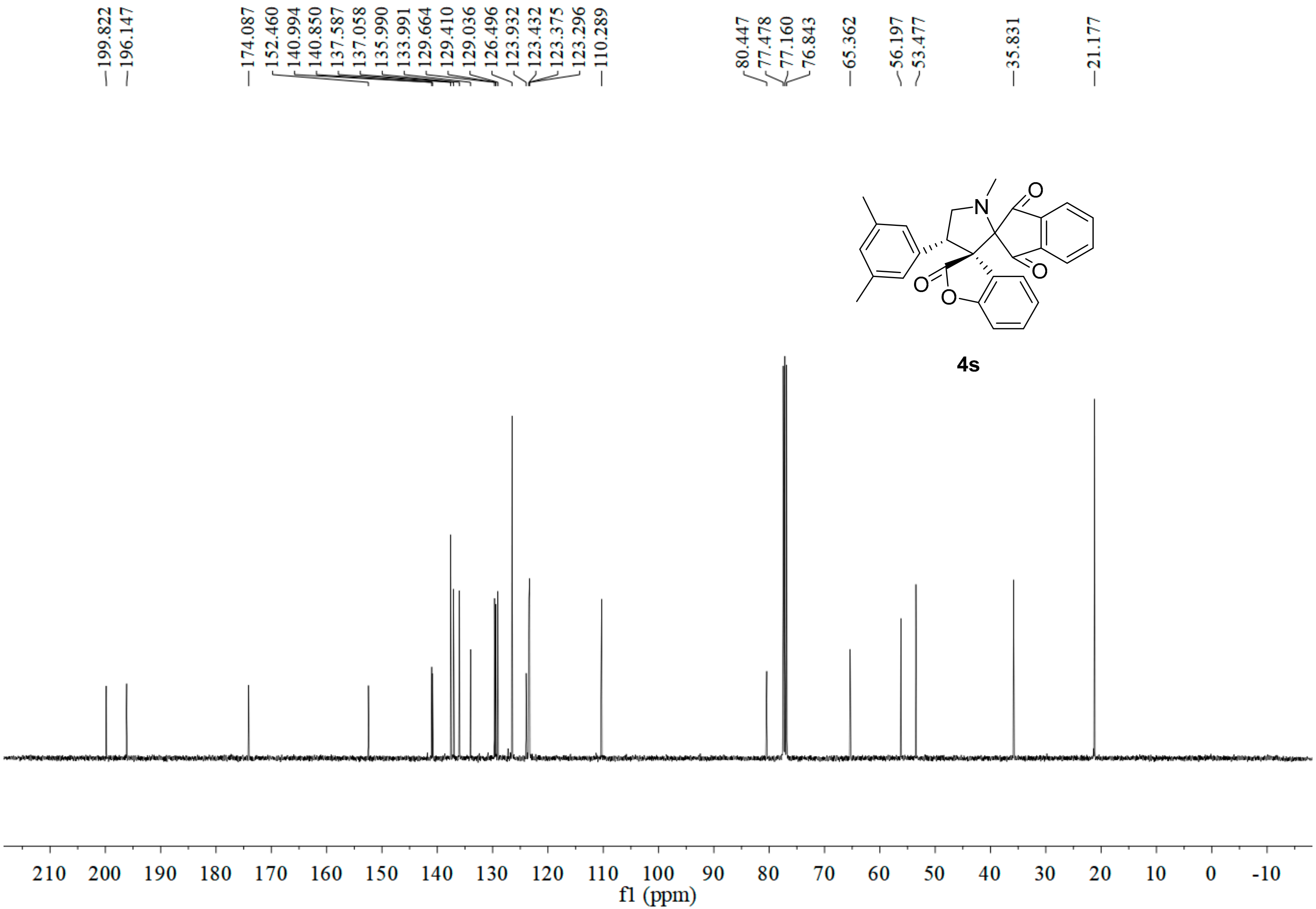

^{13}C NMR of Compound **4s** (100 MHz, $CDCl_3$)

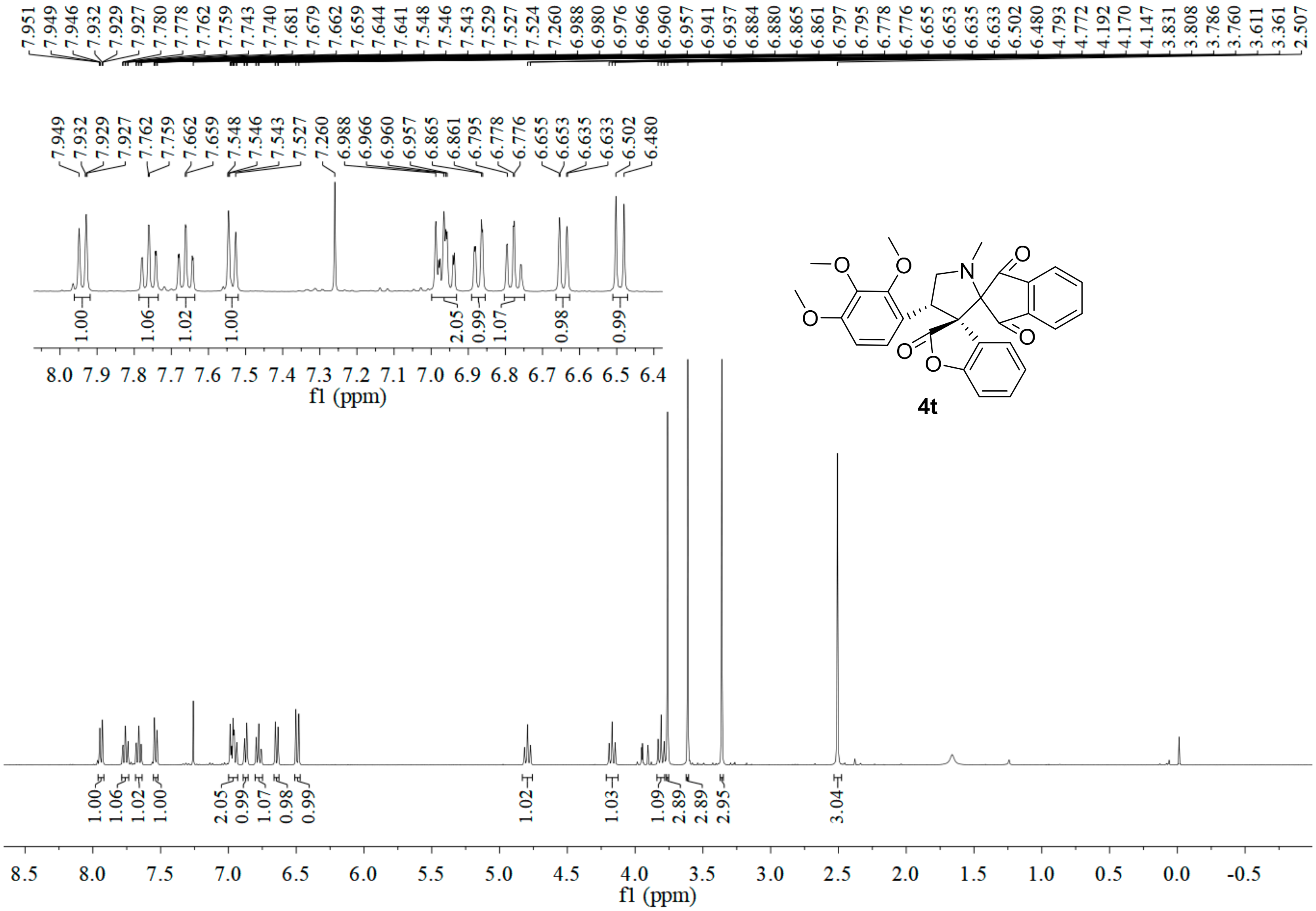

4t

^{1}H NMR of Compound **4t** (400 MHz, $CDCl_3$)

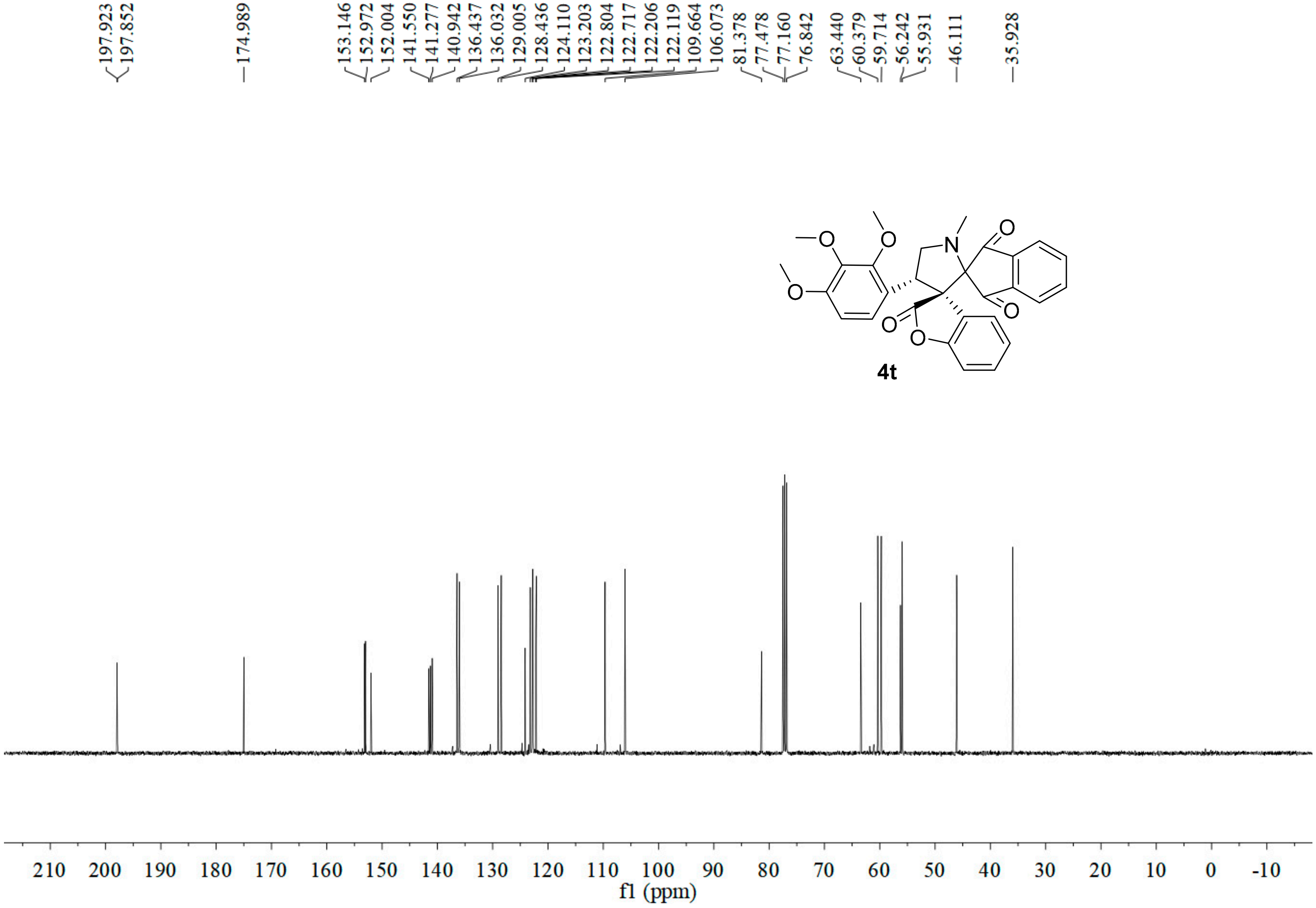

^{13}C NMR of Compound **4t** (100 MHz, $CDCl_3$)

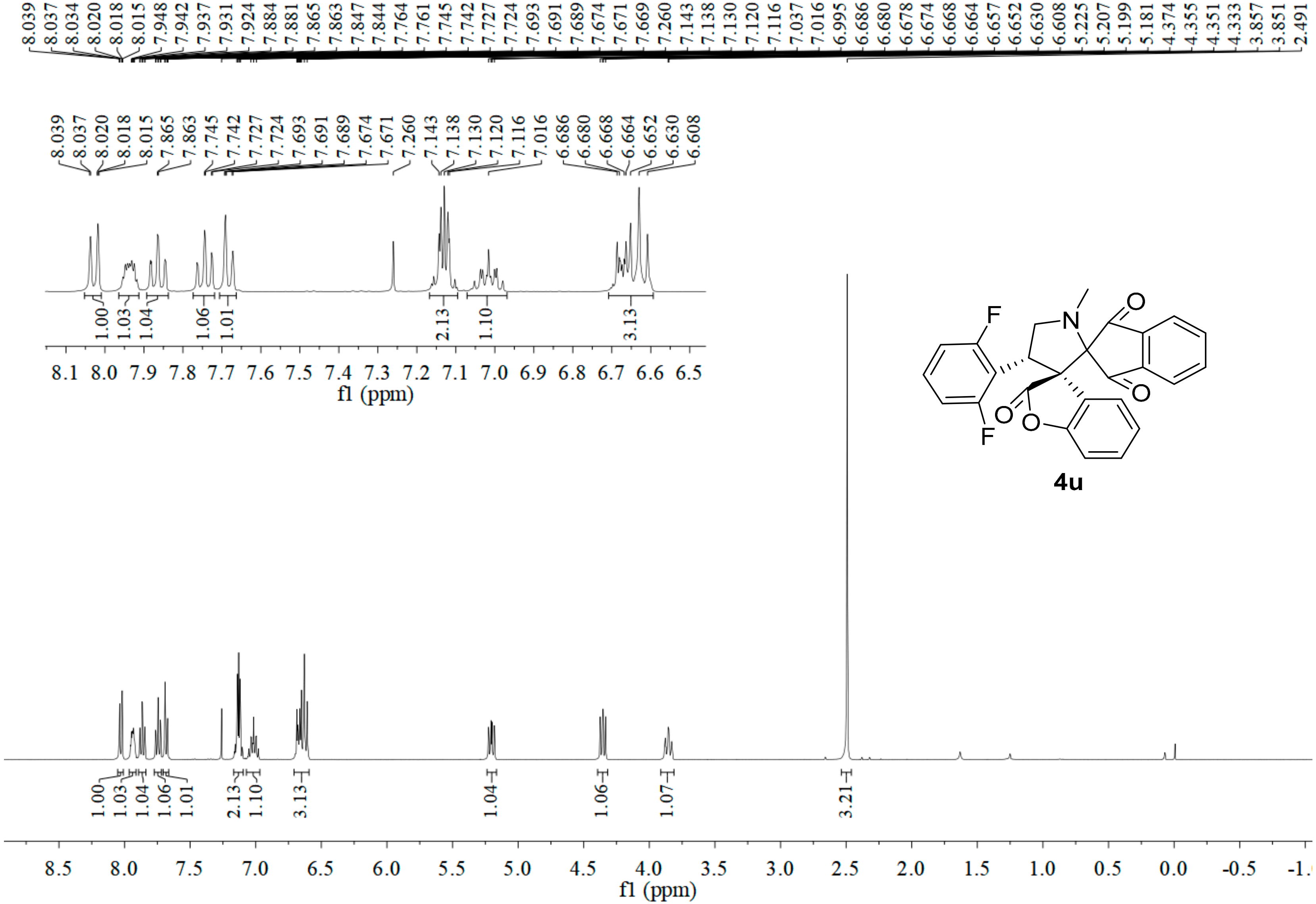

^{1}H NMR of Compound **4u** (400 MHz, $CDCl_3$)

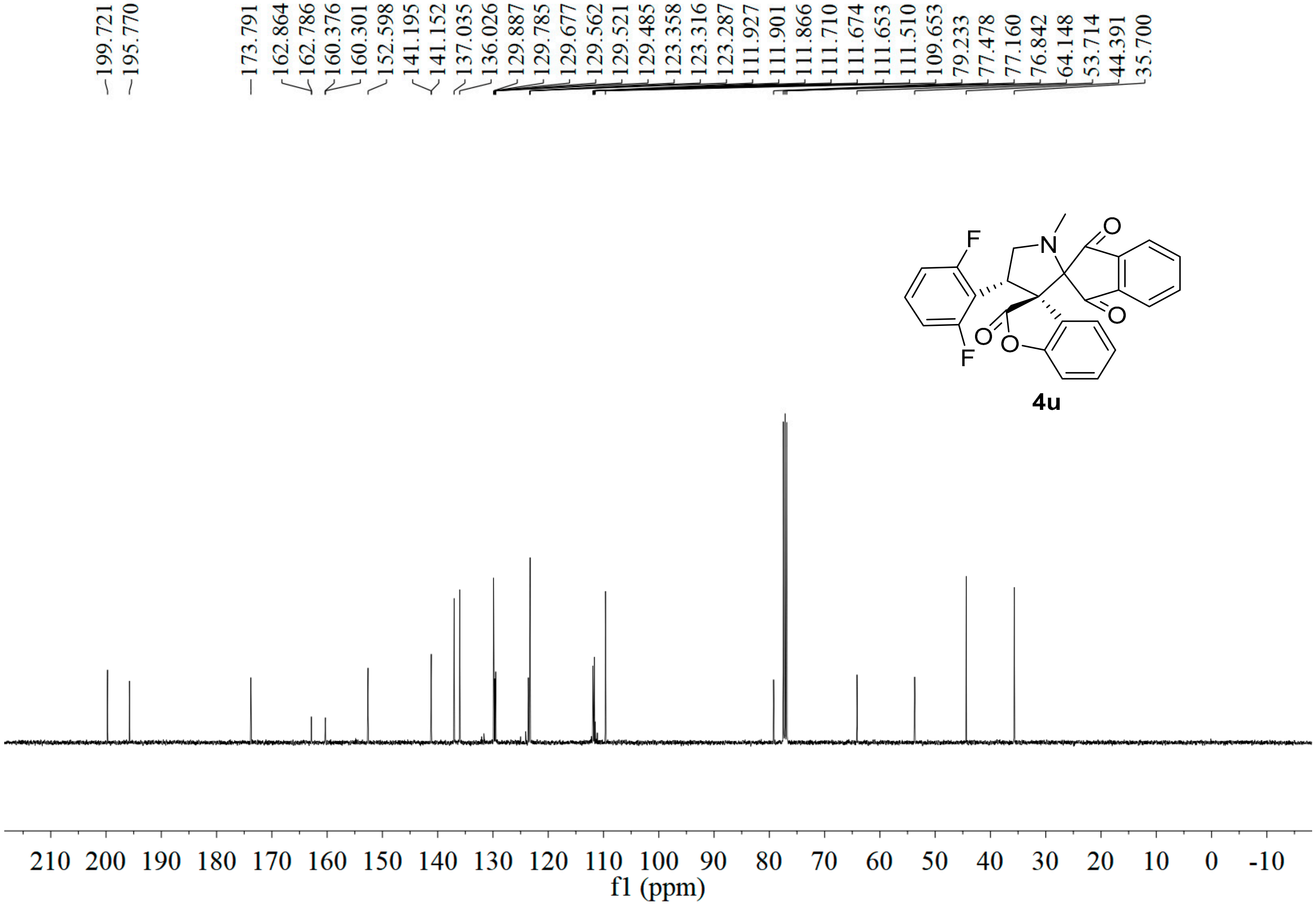

^{13}C NMR of Compound **4u** (100 MHz, $CDCl_3$)

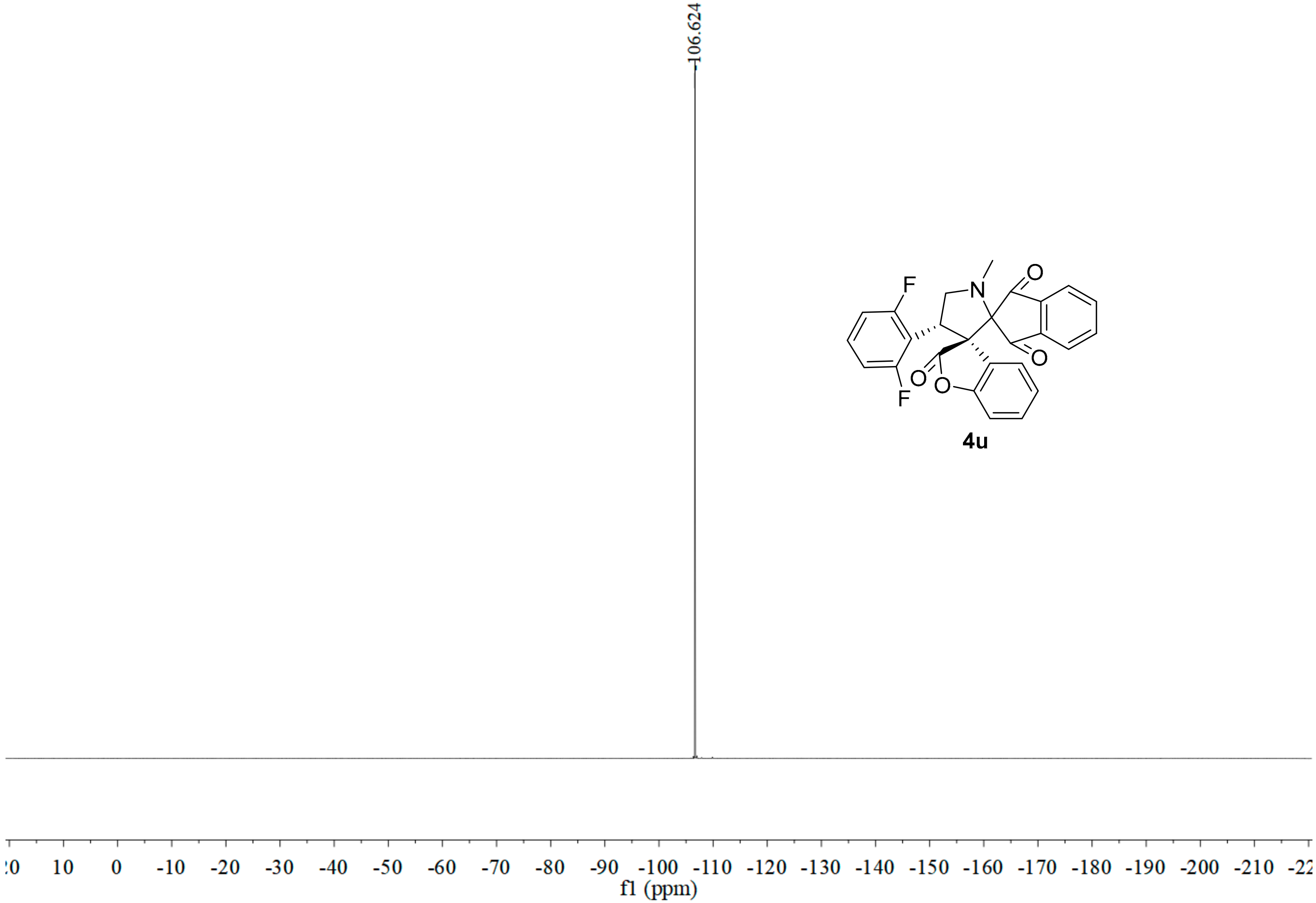

4u

^{19}F NMR of Compound **4u** (377 MHz, $CDCl_3$)

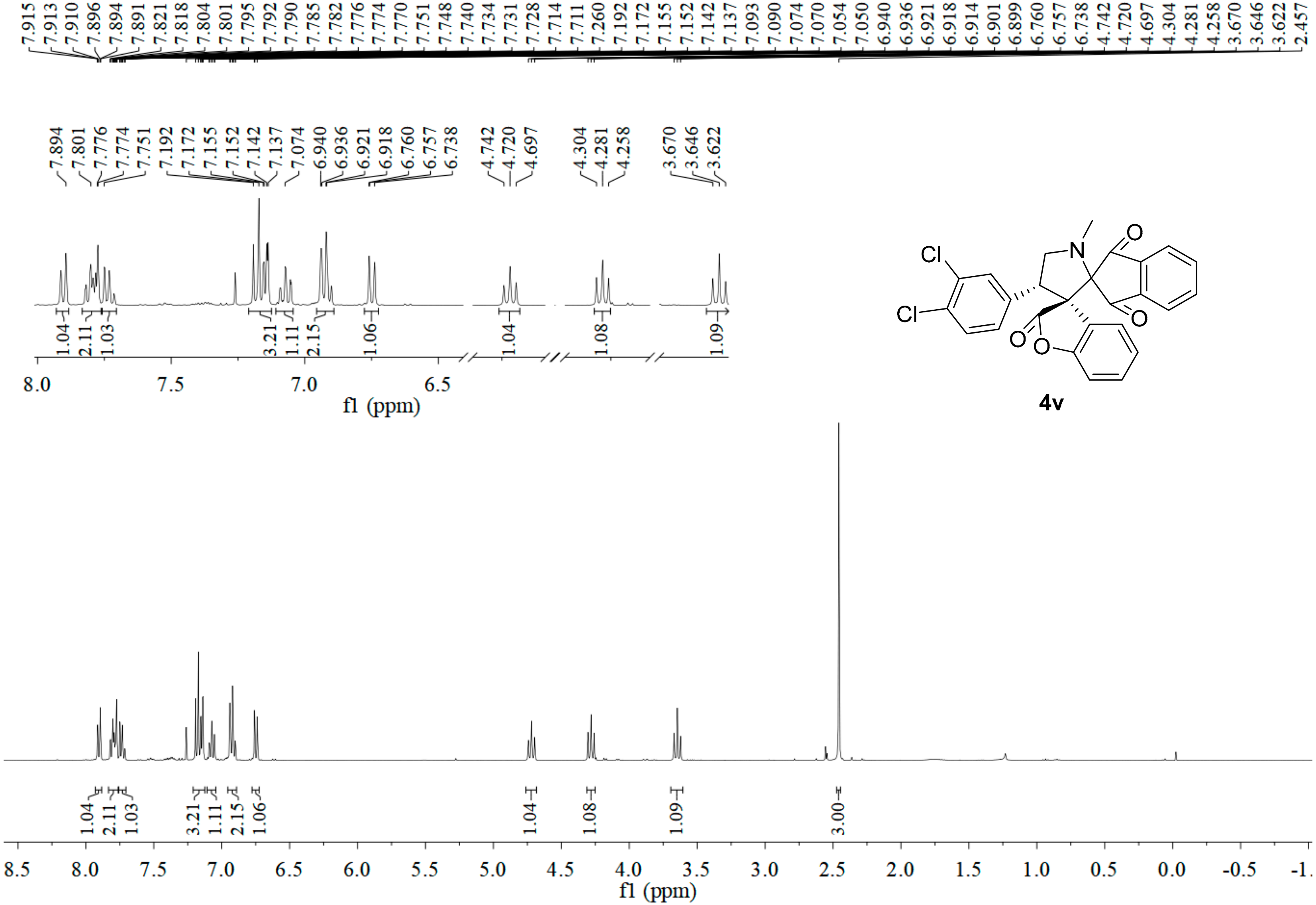

4v

^{1}H NMR of Compound **4v** (400 MHz, $CDCl_3$)

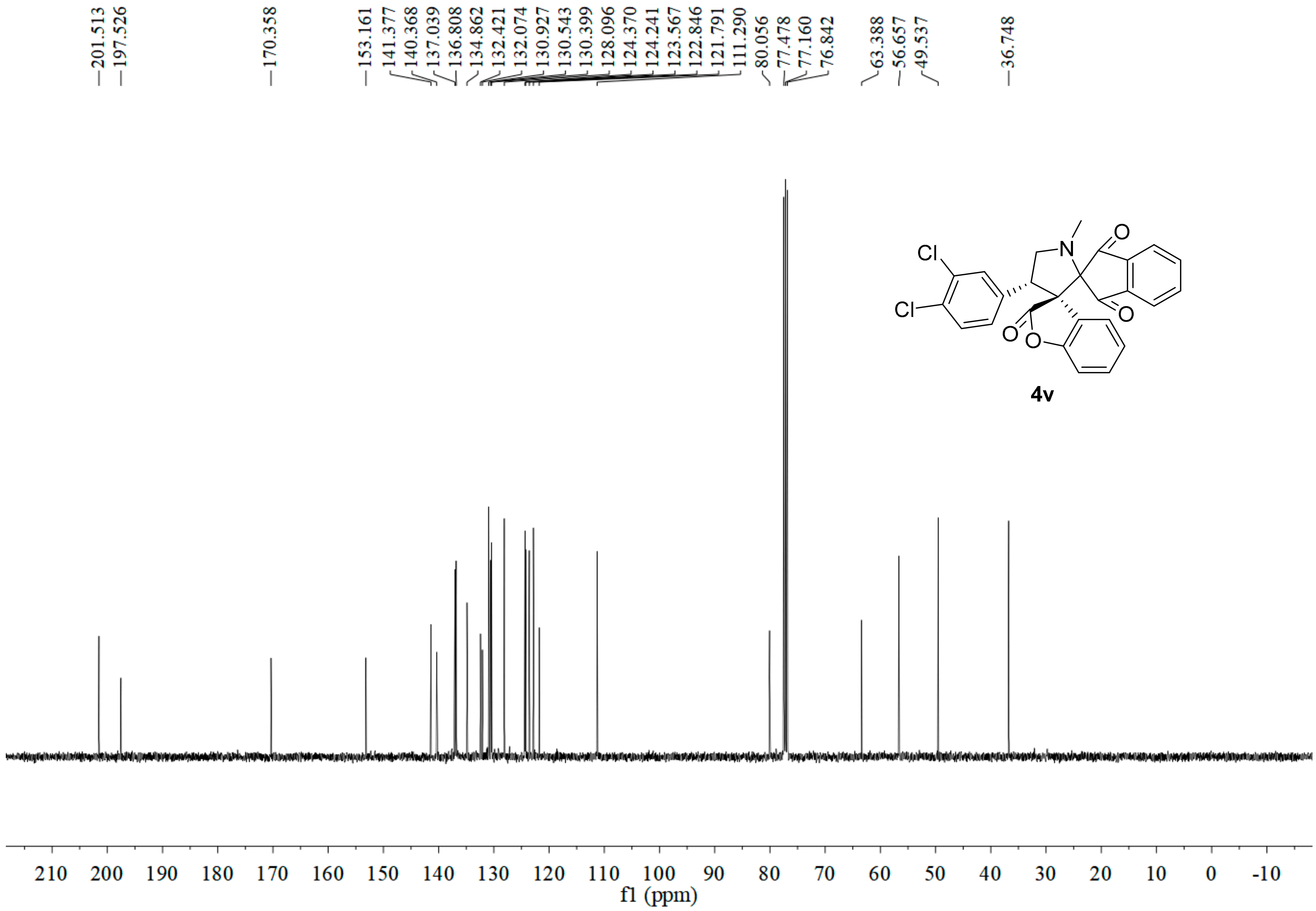

^{13}C NMR of Compound **4v** (100 MHz, $CDCl_3$)

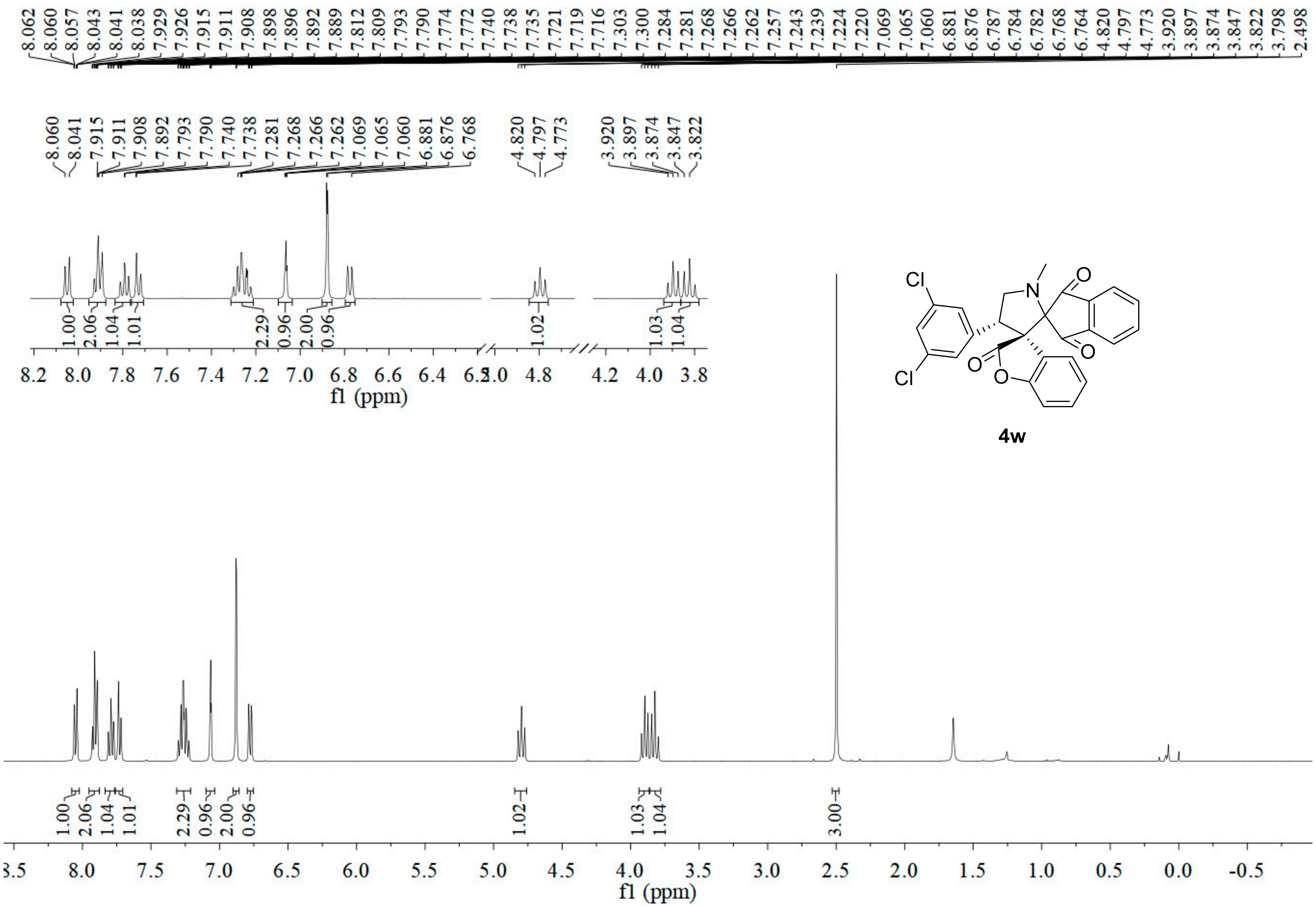

4w

^{1}H NMR of Compound **4w** (400 MHz, $CDCl_3$)

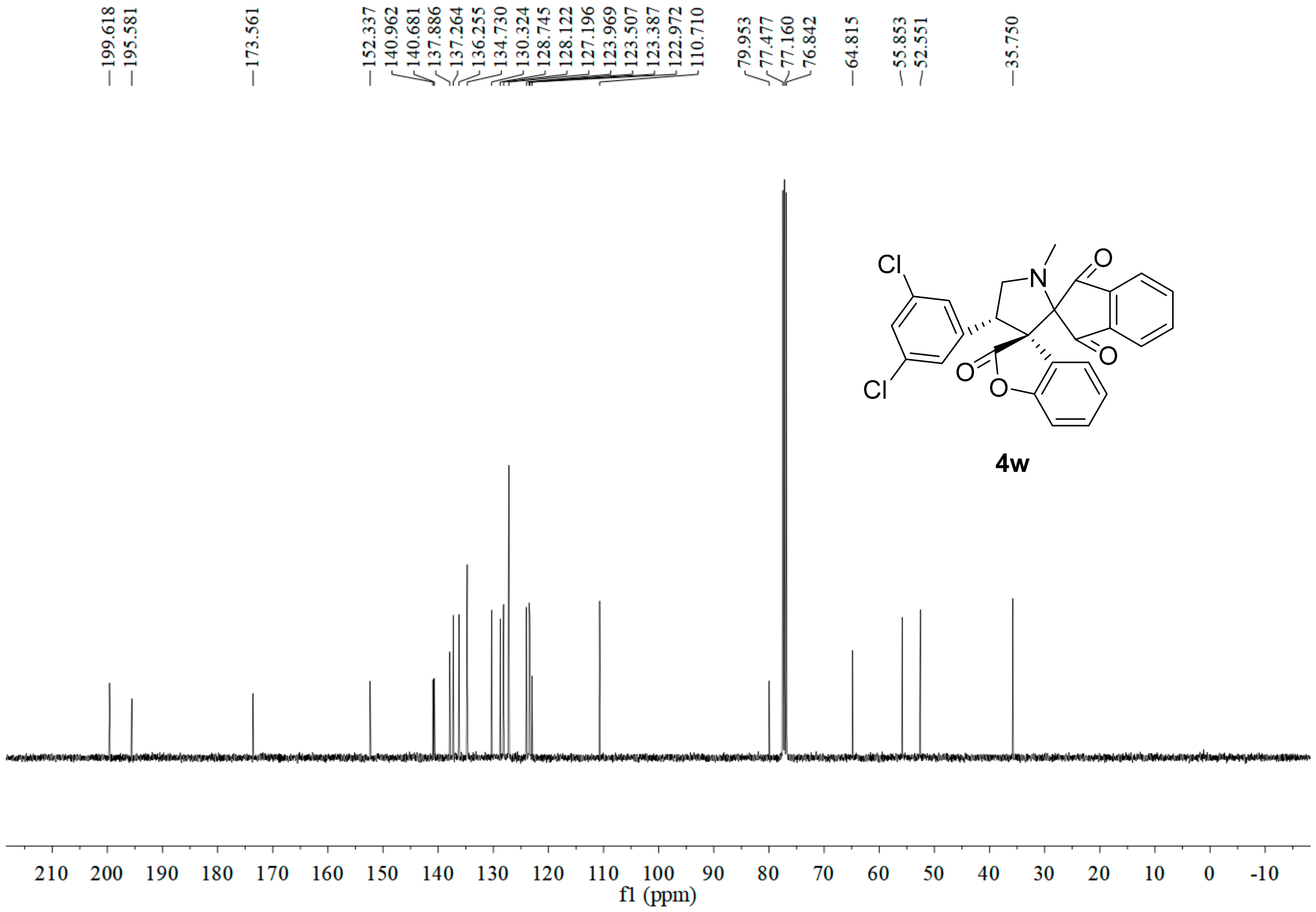

^{13}C NMR of Compound **4w** (100 MHz, $CDCl_3$)

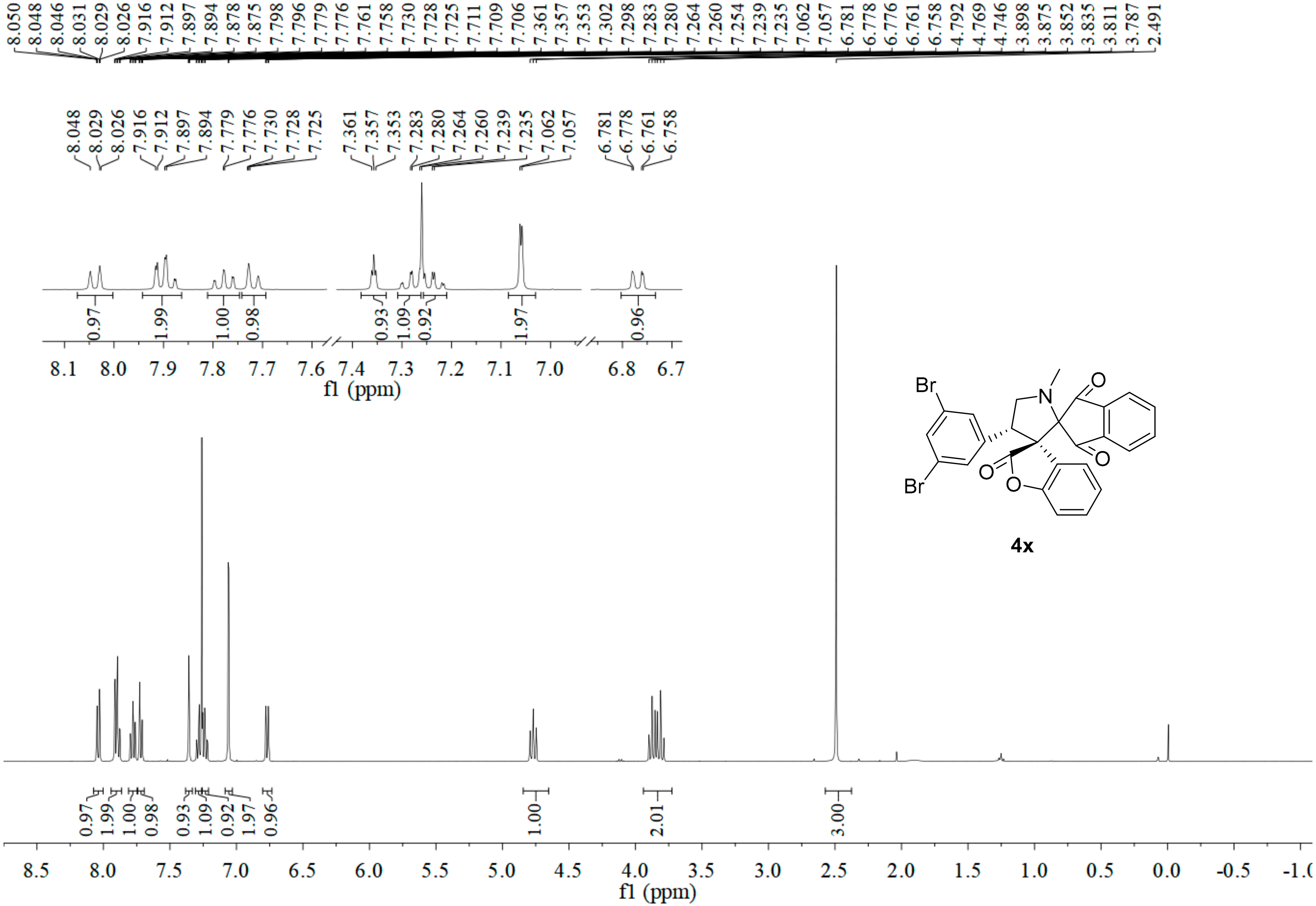

^{1}H NMR of Compound **4x** (400 MHz, $CDCl_3$)

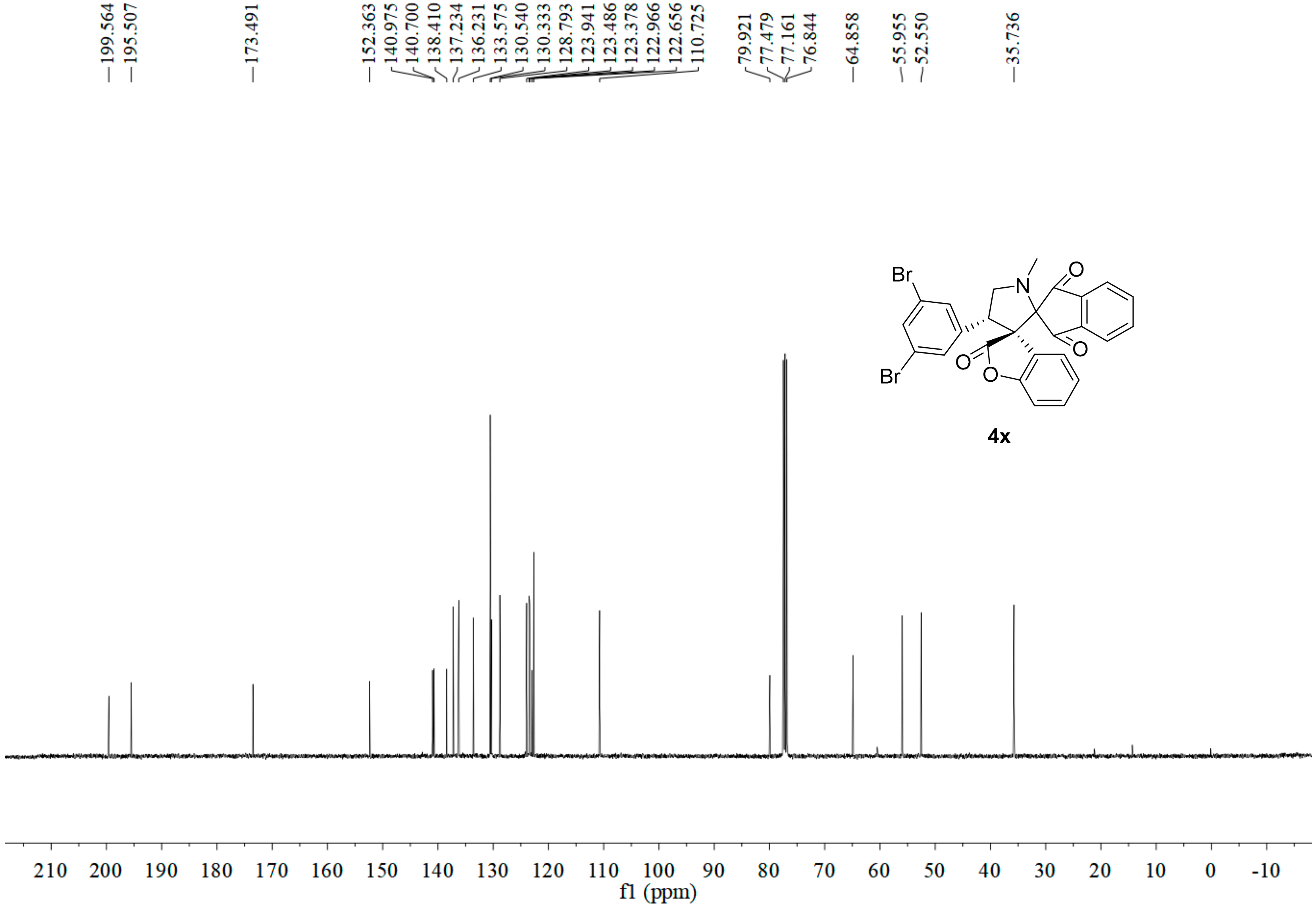

^{13}C NMR of Compound **4x** (100 MHz, $CDCl_3$)
